# Supplementary material for: Disease-modifying therapies and cost-of-illness progression among people newly diagnosed with multiple sclerosis: a national register-based cohort study covering treatment initiation with interferons, glatiramer acetate or natalizumab
Source: BMJ Open. 2023 May 16;13(5):e067516. doi: 10.1136/bmjopen-2022-067516 (PMC10193087; doi:10.1136/bmjopen-2022-067516)

Online Supplementary Material

Supplementary Table 1: Mean costs per patient per year, mean costs [95% confidence intervals] in € 2022 prices (crude observed means and confidence intervals, unadjusted), by treatment groups

	**Year 0 (year of MS diagnosis)**	**Year 1**	**Year 2**	**Year 3**	**Year 4**	**Year 5**	**Year 6**	**Year 7**	**Year 8**	**Year 9**	**Year 10**
**Interferon group**	**N=2696**	**N=2696**	**N=2644**	**N=2495**	**N=2219**	**N=1879**	**N=1556**	**N=1260**	**N=957**	**N=627**	**N=320**
Inpatient costs	1774 [1631-1918]	1079 [923-1236]	1327 [1147-1508]	1236 [1068-1405]	1148 [975-1321]	1241 [1022-1460]	1281 [1053-1509]	1082 [872-1291]	1439 [1004-1874]	1006 [707-1304]	1042 [608-1477]
Outpatient costs	1800 [1747-1853]	1368 [1313-1424]	1387 [1329-1446]	1417 [1352-1482]	1444 [1378-1510]	1528 [1453-1604]	1537 [1454-1619]	1626 [1510-1742]	1635 [1532-1738]	1726 [1594-1858]	1754 [1572-1935]
Co-payments	107 [106-109]	74 [72-75]	69 [67-71]	66 [64-68]	66 [64-68]	67 [65-69]	67 [64-69]	66 [64-69]	67 [64-70]	65 [61-69]	69 [63-74]
Drug costs	5904 [5733-6074]	11170 [10984-11357]	11029 [10811-11247]	10843 [10596-11090]	10686 [10412-10961]	10113 [9805-10421]	9850 [9504-10196]	9361 [8974-9748]	8624 [8176-9072]	8394 [7835-8954]	7927 [7120-8735]
**Total healthcare costs**	9585 [9345-9826]	13701 [13439-13962]	13813 [13512-14114]	13562 [13243-13882]	13345 [13001-13688]	12948 [12551-13346]	12734 [12303-13165]	12135 [11672-12598]	11765 [11145-12385]	11191 [10540-11842]	10792 [9892-11693]
SA costs	6927 [6469-7386]	7816 [7263-8369]	6586 [6078-7094]	5320 [4849-5790]	4649 [4167-5131]	4446 [3955-4937]	4113 [3595-4630]	3966 [3403-4529]	4410 [3688-5131]	3988 [3199-4777]	3896 [2837-4955]
DP costs	1890 [1555-2225]	2547 [2168-2926]	3887 [3433-4341]	5614 [5069-6159]	7386 [6730-8042]	8590 [7832-9348]	10068 [9172-10965]	11944 [10865-13023]	12781 [11501-14062]	14792 [13118-16466]	15204 [12888-17519]
**Total productivity losses**	8817 [8273-9362]	10356 [9709-11004]	10473 [9818-11128]	10934 [10244-11623]	12035 [11259-12811]	13036 [12175-13897]	14181 [13184-15177]	15910 [14747-17073]	17191 [15804-18578]	18780 [17023-20536]	19100 [16687-21512]
**Glatiramer Acetate group**	**N=441**	**N=441**	**N=430**	**N=406**	**N=348**	**N=292**	**N=225**	**N=170**	**N=116**	**N=66**	**N=31**
Inpatient costs	2217 [1733-2702]	1323 [862-1784]	1183 [775-1590]	1136 [731-1540]	1429 [956-1902]	1538 [911-2165]	880 [445-1315]	1401 [827-1974]	1324 [602-2047]	550 [134-967]	1417 [-911-3744]
Outpatient costs	2042 [1894-2189]	1506 [1361-1652]	1510 [1369-1651]	1566 [1407-1726]	1627 [1428-1826]	1716 [1505-1927]	1709 [1478-1940]	1734 [1475-1993]	1995 [1555-2435]	2165 [1573-2758]	2739 [1162-4316]
Co-payments	109 [104-113]	79 [74-84]	73 [69-78]	72 [67-76]	71 [65-77]	75 [69-80]	70 [64-76]	77 [69-86]	74 [64-84]	71 [60-82]	73 [57-90]
Drug costs	5670 [5227-6112]	10292 [9803-10780]	10257 [9708-10805]	9891 [9305-10476]	9211 [8553-9868]	9026 [8257-9796]	8846 [7966-9726]	9281 [8243-10320]	8390 [7164-9616]	8467 [6740-10195]	8282 [5644-10921]
**Total healthcare costs**	10037 [9341-10733]	13235 [12498-13972]	13023 [12252-13794]	12664 [11915-13414]	12338 [11483-13192]	12355 [11316-13393]	11505 [10464-12546]	12493 [11267-13719]	11783 [10289-13277]	11254 [9312-13196]	12511 [8603-16420]

SA costs	8118 [6848-9389]	8681 [7229-10132]	7190 [5834-8546]	6649 [5300-7998]	6105 [4718-7492]	5155 [3768-6541]	5154 [3627-6680]	5067 [3422-6712]	5191 [2897-7485]	4605 [2407-6803]	3815 [656-6975]
DP costs	3329 [2218-4441]	4127 [2896-5359]	5312 [3915-6709]	7615 [5945-9285]	9754 [7778-11730]	11788 [9474-14103]	13142 [10471-15812]	15090 [11772-18407]	18078 [13827-22329]	18791 [13241-24341]	19044 [11703-26384]
**Total productivity losses**	11447 [9871-13024]	12882 [11104-14661]	12502 [10690-14314]	14264 [12271-16257]	15859 [13620-18097]	16943 [14442-19444]	18295 [15397-21194]	20157 [16747-23566]	23269 [18820-27719]	23396 [17673-29118]	22859 [15118-30600]
**Natalizumab group**	**N=536**	**N=536**	**N=453**	**N=379**	**N=315**	**N=260**	**N=197**	**N=153**	**N=118**	**N=65**	**N=26**
Inpatient costs	4862 [4202-5521]	3230 [2519-3942]	2317 [1753-2881]	3043 [1683-4403]	2290 [1549-3031]	1725 [1074-2376]	1642 [920-2363]	1836 [1045-2628]	1087 [475-1699]	2951 [1095-4806]	3302 [166-6438]
Outpatient costs	2662 [2475-2848]	2580 [2372-2788]	2252 [2037-2467]	2161 [1918-2404]	2492 [2188-2795]	2254 [2007-2501]	2202 [1904-2501]	2397 [2006-2788]	2227 [1853-2601]	2831 [2238-3425]	3027 [1930-4124]
Co-payments	135 [130-140]	102 [97-107]	87 [83-91]	87 [82-92]	88 [82-94]	90 [83-96]	82 [75-89]	86 [78-94]	78 [70-86]	92 [77-107]	94 [71-117]
Drug costs	7568 [7064-8072]	16023 [15485-16560]	15930 [15291-16569]	14550 [13770-15330]	13512 [12619-14405]	13295 [12277-14312]	13368 [12214-14522]	11826 [10469-13184]	11336 [9816-12856]	11197 [8998-13396]	13386 [8164-18608]
**Total healthcare costs**	15226 [14283-16170]	22588 [21691-23484]	20586 [19724-21447]	19840 [18220-21461]	18382 [17204-19560]	17364 [16240-18489]	17293 [16013-18573]	16147 [14604-17689]	14728 [12909-16546]	17071 [14005-20137]	19809 [13284-26335]
SA costs	9683 [8492-10875]	11855 [10312-13399]	9867 [8358-11376]	7751 [6306-9197]	7484 [5784-9184]	7023 [5309-8737]	5292 [3639-6946]	5402 [3574-7229]	4788 [2807-6770]	5343 [2248-8438]	3317 [287-6348]
DP costs	2327 [1498-3157]	3392 [2388-4396]	5034 [3727-6341]	7629 [5942-9317]	9239 [7241-11237]	12062 [9629-14495]	14303 [11429-17177]	15873 [12321-19425]	17963 [13694-22233]	19921 [13856-25986]	20632 [10822-30443]
**Total productivity losses**	12010 [10646-13375]	15241 [13486-16996]	14901 [13047-16755]	15381 [13343-17418]	16723 [14354-19091]	19085 [16427-21743]	19596 [16559-22632]	21275 [17620-24930]	22752 [18472-27032]	25264 [19184-31343]	23950 [14408-33491]

Supplementary Figure 1: EDSS score progression from baseline (index year) to the end of follow-up, by IFN vs GA vs NAT treatment groups

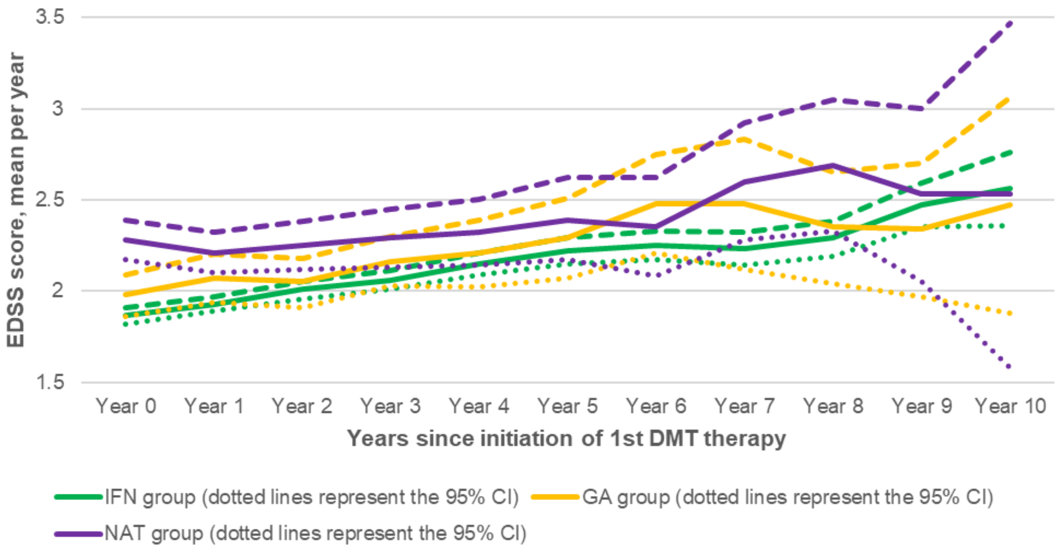

Supplementary Figure 2 (a-f): Progression (estimated mean from the regression) for all cost components, from baseline (year at MS diagnosis) to the end of follow-up, by treatment groups, adjusted for disability progression during follow-up

A) Inpatient costs

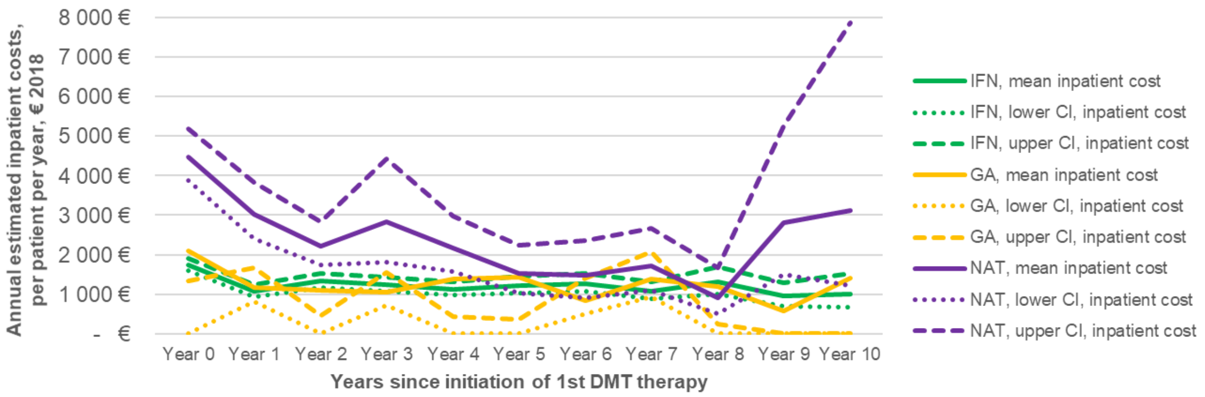

B) Outpatient costs

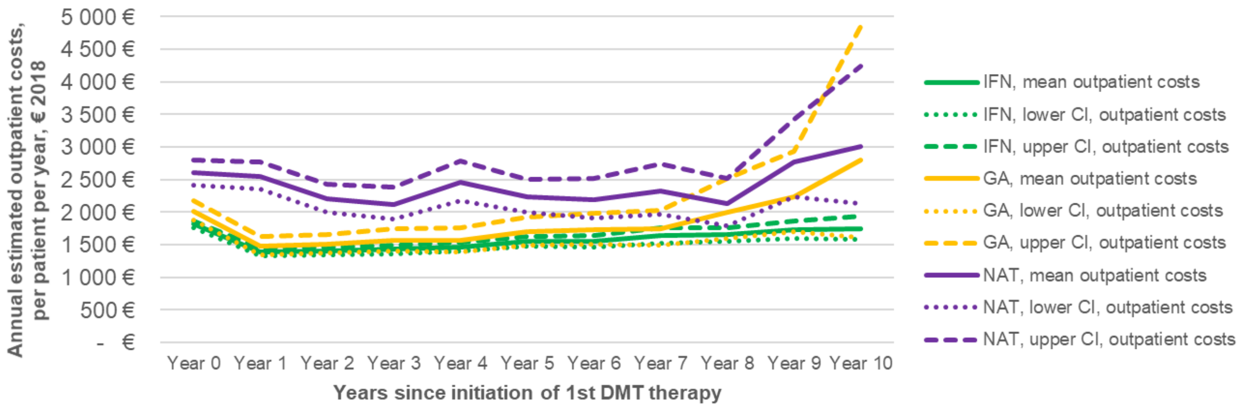

C) Co-payments

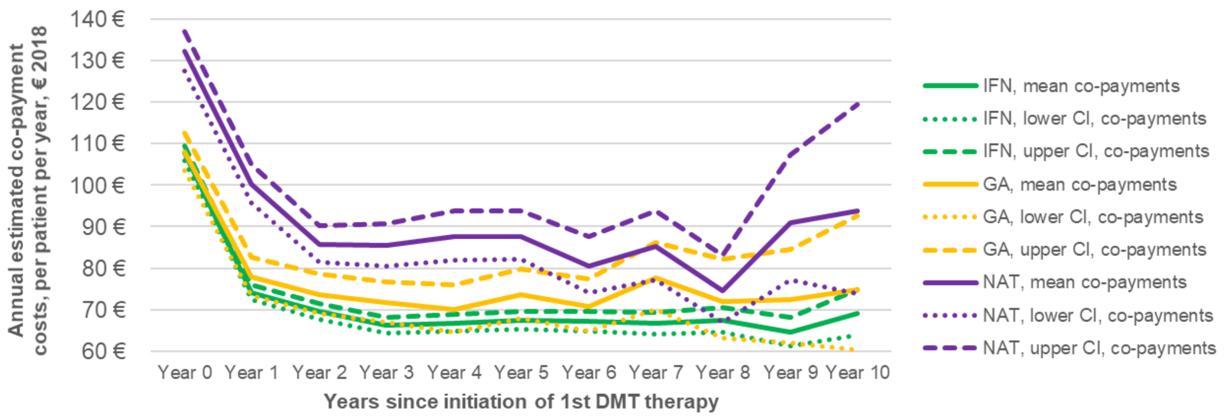

D) Drug costs

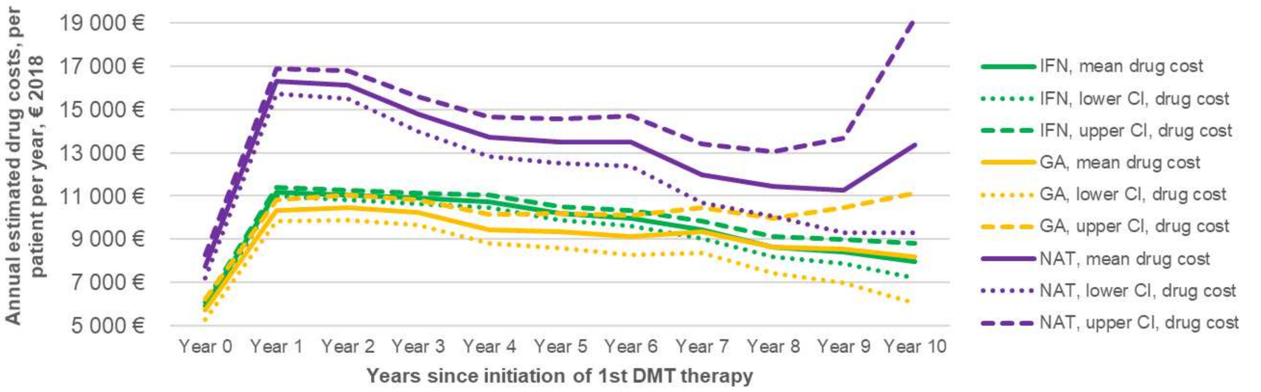

E) Sickness absence costs

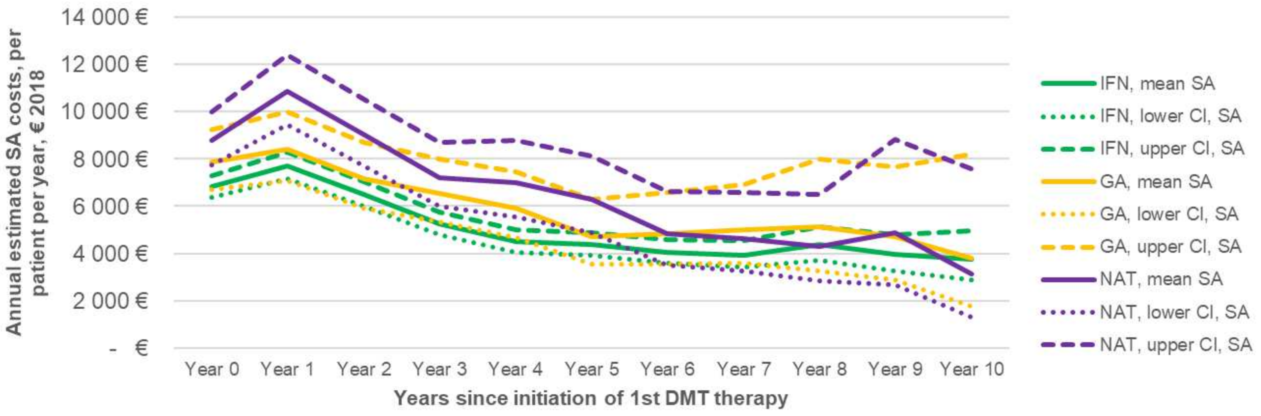

F) Disability pension costs

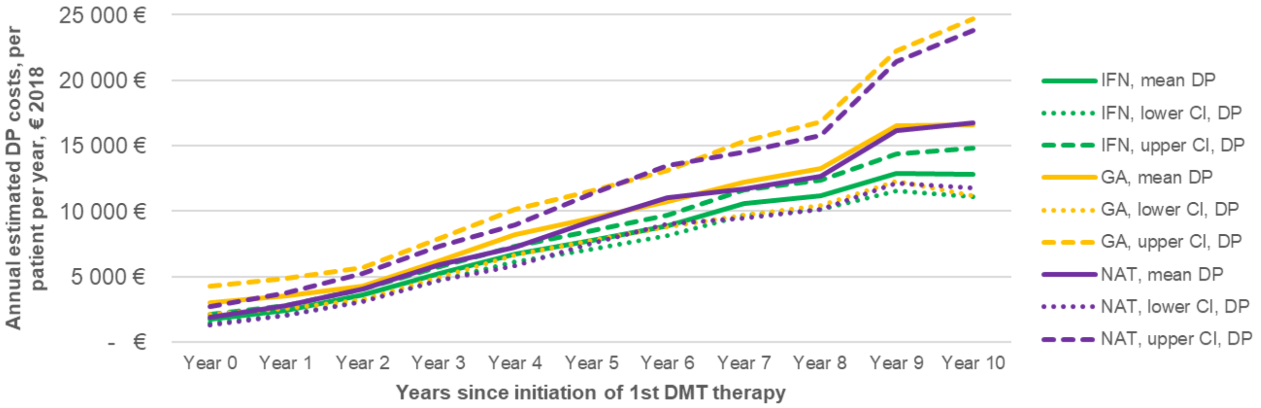

Supplement: Supplementary data [file bmjopen-2022-067516supp001.pdf]
