# Supplementary material for: Disease-modifying therapies and cost-of-illness progression among people newly diagnosed with multiple sclerosis: a national register-based cohort study covering treatment initiation with interferons, glatiramer acetate or natalizumab
Source: BMJ Open. 2023 May 16;13(5):e067516. doi: 10.1136/bmjopen-2022-067516 (PMC10193087; doi:10.1136/bmjopen-2022-067516)
Supplement: Author's manuscript [file bmjopen-2022-067516.draft_revisions.pdf]

BMJ Open is committed to open peer review. As part of this commitment we make the peer review history of every article we publish publicly available.

When an article is published we post the peer reviewers' comments and the authors' responses online. We also post the versions of the paper that were used during peer review. These are the versions that the peer review comments apply to.

The versions of the paper that follow are the versions that were submitted during the peer review process. They are not the versions of record or the final published versions. They should not be cited or distributed as the published version of this manuscript.

BMJ Open is an open access journal and the full, final, typeset and author-corrected version of record of the manuscript is available on our site with no access controls, subscription charges or pay-per-view fees (http://bmjopen.bmj.com).

If you have any questions on BMJ Open's open peer review process please email info.bmjopen@bmj.com

BMJ Open

Cost-of-illness progression among people newly diagnosed with multiple sclerosis: a prospective cohort study regarding treatment initiation with interferons, glatiramer acetate, or natalizumab

Journal:	*BMJ Open*
Manuscript ID	bmjopen-2022-067516
Article Type:	Original research
Date Submitted by the Author:	06-Sep-2022
Complete List of Authors:	Karampampa, Korinna; Karolinska Institutet, Department of Clinical Neuroscience<br>Gyllensten, Hanna; University of Gothenburg, Institute of Health and Care Sciences; University of Gothenburg Centre for Person-Centred Care,<br>Friberg, Emilie; Karolinska Institute<br>Murley, Chantelle; Karolinska Institutet Department of Clinical Neuroscience, Clinical Neuroscience<br>Kavaliunas, A; Karolinska Universitetssjukhuset<br>Hillert, Jan; Karolinska Institute, Clinical Neuroscience<br>Olsson, Tomas; Karolinska Institute<br>Alexanderson, Kristina; karolinska institutet, Department of Clinical Neuroscience
Keywords:	Multiple sclerosis < NEUROLOGY, HEALTH ECONOMICS, Health economics < HEALTH SERVICES ADMINISTRATION & MANAGEMENT

SCHOLARONE™
Manuscripts

I, the Submitting Author has the right to grant and does grant on behalf of all authors of the Work (as defined in the below author licence), an exclusive licence and/or a non-exclusive licence for contributions from authors who are: i) UK Crown employees; ii) where BMJ has agreed a CC-BY licence shall apply, and/or iii) in accordance with the terms applicable for US Federal Government officers or employees acting as part of their official duties; on a worldwide, perpetual, irrevocable, royalty-free basis to BMJ Publishing Group Ltd ("BMJ") its licensees and where the relevant Journal is co-owned by BMJ to the co-owners of the Journal, to publish the Work in this journal and any other BMJ products and to exploit all rights, as set out in our licence.

The Submitting Author accepts and understands that any supply made under these terms is made by BMJ to the Submitting Author unless you are acting as an employee on behalf of your employer or a postgraduate student of an affiliated institution which is paying any applicable article publishing charge ("APC") for Open Access articles. Where the Submitting Author wishes to make the Work available on an Open Access basis (and intends to pay the relevant APC), the terms of reuse of such Open Access shall be governed by a Creative Commons licence – details of these licences and which Creative Commons licence will apply to this Work are set out in our licence referred to above.

Other than as permitted in any relevant BMJ Author's Self Archiving Policies, I confirm this Work has not been accepted for publication elsewhere, is not being considered for publication elsewhere and does not duplicate material already published. I confirm all authors consent to publication of this Work and authorise the granting of this licence.

For peer review only

Manuscript, Version 20220815 final draft

Cost-of-illness progression among people newly diagnosed with multiple sclerosis: a prospective cohort study regarding treatment initiation with interferons, glatiramer acetate, or natalizumab

Authors: Korinna Karampampa[1], Hanna Gyllensten[2], Emilie Friberg[1], Chantelle Murley[1], , Andrius Kavaliunas[1], Jan Hillert[1],Tomas Olsson[1], Kristina Alexanderson[1]

Affiliations:

[1]Department of Clinical Neuroscience, Karolinska Institutet, SE-171 77 Stockholm, Sweden

[2]Institute of Health and Care Science, University of Gothenburg, Gothenburg, Sweden

Corresponding author:

Korinna Karampampa, MSc, PhD

Division of Insurance Medicine, Department of Clinical Neuroscience

Karolinska Institutet, SE-171 77 Stockholm, Sweden

E-mail: korinna.karampampa@ki.se

Phone: +46 704 33 74 15

FAX: +46 (0)8 524 832 05

For peer review only

ABSTRACT

Objectives: Since Disease modifying therapies (DMTs) can slow disease progression in multiple sclerosis (MS), the objective of this study was to assess their impact on the cost of illness (COI) progression in MS.

Setting: An observational study utilizing data from nationwide registers in Sweden.

Participants: People with MS (PwMS) in Sweden first diagnosed in 2006-2015, when aged 20-55, receiving first-line therapy with interferons (IFN), glatiramer acetate (GA), or natalizumab (NAT). They were followed in nationwide registers until 2016.

Primary and secondary outcome measures: Outcomes (in Euros, €) were: 1) healthcare costs: specialized outpatient and inpatient care, and out-of-pocket expenditure, DMTs and prescribed drugs, and 2) productivity losses: sickness absence and disability pension. Descriptive statistics and Poisson regression were computed, adjusting for disability progression using the Expanded Disability Status Scale.

Results: 3673 new PwMS were identified who were treated with IFN (N=2696), GA (N=441), or NAT (N=536). Healthcare costs were similar for the INF and GA, while higher for NAT (p-value<0.05), due to higher DMT and outpatient costs. IFN had lower productivity losses vs. NAT and GA (p-value<0.05), driven by lower sickness absence days Disability pension costs were lower for NAT vs. GA.

Conclusions: COI serves as an objective measure to explore the importance of DMTs in maintaining low levels of progression of MS over time.

For peer review only - http://bmjopen.bmj.com/site/about/guidelines.xhtml

For peer review only

1 Introduction

Multiple sclerosis (MS) is a highly disabling chronic neurological disease[1], usually diagnosed when aged 20-40 years[1]. Sweden has one of the highest prevalence of MS in Europe, estimated at 189 cases over 100,000 individuals[2].

In 2010 in Sweden, the annual cost of illness (COI) of MS, from a societal perspective, was approximately EUR 414 million[3]. Productivity losses[3] and the cost of disease modifying therapies (DMT) were the two main cost drivers[4].

The COI in MS has been shown to increase with disease's disability progression[4]. On the other hand, treatment with DMTs, has been associated with slowing this progression; possibly reducing the increase in the COI in MS over time[5].

Until now, only few observational studies have been conducted evaluating the COI progression in MS in relation to DMTs, which however do not have a longitudinal design[6], and are also not accounting for disability progression.

The aim of this study was to explore the COI progression in MS among newly diagnosed people with MS (PwMS) in Sweden, in relation to the first DMT received (interferons (IFN) vs. glatiramer acetate (GA) vs. natalizumab (NAT)), adjusting for disability progression over time.

For peer review only - http://bmjopen.bmj.com/site/about/guidelines.xhtml

2 Material and Methods

This was a register-based prospective cohort study of PwMS first diagnosed with MS in 2006-2015 (index year), when aged 20-55 years. They were identified from the Swedish MS register (SMSreg)[7] and those who received IFN, GA, or NAT within 1 month after diagnosis and for at least 6 months, were included. The maximum age of 55 was chosen to allow follow-up until ordinary retirement age of 65.

The cohort of PwMS were followed until maximum 2016 in nationwide registers[7, 8]. The following data, kept by four authorities, were used:

- Stockholm Region:
  - Swedish MS register (SMSreg)[7]: diagnosis age and date, type of MS, Expanded Disability Status Scale (EDSS), and type and date of DMT treatment
- National Board of Health and Welfare:
  - National Patient Register (NPR)[8]: dates and diagnoses for inpatient and specialized outpatient healthcare
  - Swedish Prescribed Drug Register (SPDR)[8]: dates, names, and costs, for all prescribed drugs dispensed from pharmacies
  - Cause of Death Register[8]: year of death
- Statistics Sweden: Longitudinal Integration Database for Health Insurance and Labor Market Studies[8]; sex, birth year, education, country of birth, type of living area, family situation, indicator for receiving income from work and/or benefits during each calendar year
- Swedish Social Insurance Agency: Micro Data for the Analysis of Social Insurance register[8]; dates, diagnoses, and grade (full- or part-time) of sickness absence (SA) in SA spells >14 days and disability pension (DP).

For peer review only - http://bmjopen.bmj.com/site/about/guidelines.xhtml

The registers were linked using the unique personal identity number that all residents in Sweden are assigned.

PwMS who switched from one DMT group to another relevant group before the 6-months cut-off, were allocated to the later DMT group.

Never-users of DMTs, those not on IFN/GA/NAT within 1 month from MS diagnosis, and/or not receiving it for at least 6 months after treatment initiation, those receiving a DMT before MS diagnosis, and those who were not living in Sweden during the index year, were not included in the study. In analyses, PwMS were censored at migration or death.

The project received ethical approval from the Regional Ethical Review Board in Stockholm. Approval numbers: 2007/762-31; 2009/23-32; 2009/1917-32; 2010/466-32; 2011/806-32; 2011/1710-32; and 2014/236-32.

2.1 Patient and public involvement

No patients or members of the general public were enrolled as study participants, or were involved in the design of/conducting this study.

2.2 Study outcomes

The following sociodemographic characteristics, measured at the year of diagnosis, were considered: sex, age at index year, educational level, country of birth, type of living area, and family situation.

Four types of MS at diagnosis were identified, based on information available from SMSreg: Relapsing-Remitting MS (RRMS), Progressive-Relapsing MS (PRMS), Primary-Progressive MS (PPMS), and Secondary-Progressive MS (SPMS).

Multi-morbidity in the index year was derived utilizing the Rx-Risk Comorbidity Index[9], based on the type of drugs prescribed to PwMS according to SPDR.

For peer review only - http://bmjopen.bmj.com/site/about/guidelines.xhtml

Disability in MS was based on information from the Expanded Disability Status Scale (EDSS) available. The score of EDSS ranges from 0 to 10, with 0.5 step intervals (0 indicating no impairment, while 10 indicating death from MS)[10]. A clinically meaningful change in the EDSS score is a change of at least one point in patients with EDSS <5.5, and 0.5 point for those with EDSS of ≥5.5[11].

Where multiple EDSS scores for each calendar year were available, the highest EDSS value was retained. In case of missing EDSS scores, the mean annual score was used, computed based on scores from PwMS in the same DMT group and index year, and with the same type of MS.

Healthcare resources consumed by PwMS as well as their SA and DP days were calculated and multiplied with unit costs (Table 1) to determine the societal COI for MS. The estimated COI included all costs occurring for PwMS during a calendar year, irrespective of the main diagnosis behind the cost.

Inpatient and outpatient costs were calculated for each year multiplying the number of days/visits, derived from NPR, with the cost per 1.0 retrospective nationwide weight from the diagnosis-related groups (DRG) system. Co-payment costs for these visits were also estimated by summing daily/per visit fees for inpatient and outpatient care. We assumed that the reimbursement period starts on 1 January, so costs exceeding the ceiling in Sweden for co-payments for one year's period, were set to the maximum amount allowed per year (Table 1).

The cost of dispensed drugs was derived from SPDR, based on the pharmacy's retail price of each dispensed drug package, including the co-payment paid by patients. The annual cost of drugs was calculated by summing the cost for all dispensed drugs during each year.

The cost of DMTs not available through SPDR, i.e., MS drugs not dispensed by pharmacies, but used as MS treatment in the hospital setting (natalizumab and rituximab for the

For peer review only - http://bmjopen.bmj.com/site/about/guidelines.xhtml

study period), was calculated using information in SMSreg. The number of months on these treatments was multiplied with the cost per month of treatment (Table 1).

By adding costs of inpatient and outpatient care and co-payments for inpatient and outpatient care and drugs, the total annual healthcare costs per patient were calculated. Productivity losses were estimated based on the net days with SA and DP per year, multiplied with the age-adjusted mean salary, adding social security contributions made by employers (Table 1), using the human capital approach[12].

All people living in Sweden with income from work or unemployment benefits are from 16 years of age covered by the public SA insurance system and can be granted SA benefits if their work capacity is reduced due to disease or injury. The employer usually reimburses income loss during days 2-14, after which SA benefits are administered by the Swedish Social Insurance Agency; for unemployed individuals, this happens from day 2. Therefore, to prevent bias in relation to employment status, only SA >14 days was included.

All people aged 19-65 years can be granted DP if their work capacity is long-term or permanently reduced[13]. Both SA and DP can be granted for full-time (100%) or part-time (75%, 50%, or 25%) of ordinary work hours[13], making it possible to have both partial SA and DP concurrently. Hence, we combined them to calculate the net days of SA and DP.

All costs were inflated to 2018 prices, applying the harmonized index for consumer prices[14]. Costs calculated in Swedish Krona (SEK) were then converted to Euros (EUR) in 2018 values (Table 1).

Table 1: Unit costs used in the calculation of healthcare costs and productivity losses

	Year	Value in 2018 SEK	Value in 2018 Euros[1]	Source
Average inpatient and outpatient cost per	2006	50,973 kr	4,969 €	Swedish Association of Local Authorities and Regions [Sveriges
	2007	50,224 kr	4,896 €	

For peer review only

For peer review only - http://bmjopen.bmj.com/site/about/guidelines.xhtml

1.0 DRG	2008	51,388 kr	5,009 €	Kommuner och Regioner], KPP Somatik vård [15]
	2009	51,785 kr	5,048 €	
	2010	50,457 kr	4,919 €	
	2011	50,286 kr	4,902 €	
	2012	49,820 kr	4,857 €	
	2013	51,468 kr	5,017 €	
	2014	53,388 kr	5,204 €	
	2015	55,807 kr	5,440 €	
	2016	57,334 kr	5,589 €	
Co-payment for hospital stay (cost per day of stay)	2018	100 kr	10 €	Assume 100 SEK per day, as this is the case for the majority of the regions (including Stockholm).[16] The max co-payment amount for inpatient care was set to 1500 SEK per year (assumption for whole Sweden, based on information from the region Västra Götaland)[17]
Co-payment for visit in specialised care (cost per visit)	2018	273 kr	27 €	Swedish Association of Local Authorities and Regions (18). The max co-payment amount for outpatient care was set to 1100 SEK per year. Only one region in Sweden has a max co-payment less than 1100 SEK; so it was assumed 1100 SEK for the entire country. Swedish Association of Local Authorities and Regions[16]
Cost per month for natalizumab	2018	15,839 kr	1,544 €	Treatment with natalizumab is every 4th week (i.e. one per month)[18]; therefore, the cost per month of natalizumab was assumed to be the price for one dose of natalizumab (pharmacy's retail price)[18], excluding the cost of administration, which is captured in this study as an outpatient visit to the hospital
Cost per month for rituximab	2018	2,022 kr	197 €	Rituximab is used off-label in the treatment of MS; therefore, the exact treatment dosing was not available in the Swedish guidelines for MS treatment. A recent published study in Sweden regarding the use of rituximab for PwMS indicated that the drug dose is 500mg to 1000mg per treatment regime (here we assumed the mean, i.e 750mg per treatment regime), and the mean treatment interval for RRMS PwMS was 7.2 months per year[19]. The cost per month was then assumed to be 1.5 times the pharmacy's retail price per 500mg of rituximab injection, which was taken from FASS (9703.61 SEK)[20], divided with the frequency of treatment regimes in months (frequency: every 7.2 months) [19].
Monthly salary	2006	40,930 kr	4,248 €	The average age-adjusted monthly

For peer review only - http://bmjopen.bmj.com/site/about/guidelines.xhtml

including employer contributions	2007	41,049 kr	4,260 €	wage (2018 values) for all employment types was retrieved[21]. It was multiplied with the annual employer contributions, available from the Swedish Tax Authority[22]. The final salary was then inflated to 2018 prices using annual Harmonised Indices of Consumer Prices [HICP] for healthcare available from Eurostat[14].
	2008	40,042 kr	4,156 €	
	2009	44,583 kr	4,627 €	
	2010	44,080 kr	4,575 €	
	2011	44,730 kr	4,642 €	
	2012	44,748 kr	4,644 €	
	2013	45,051 kr	4,676 €	
	2014	46,120 kr	4,787 €	
	2015	50,300 kr	5,221 €	
	2016	48,146 kr	4,997 €	

[1]The annual exchange rate for 2018 from SEK to Euros that was used was 10.2583. Source: Eurostat, Annual Exchange Rates Euro/ECU

2.3 Analyses

Sociodemographic characteristics, multi-morbidity, type of MS at diagnosis, MS disability progression, average COI per patient and year, including 95% confidence intervals (CI), were calculated for each of the three treatment groups.

Pearson's chi-squared test[23] and the likelihood ratio test[23] were used to explore whether the observed sociodemographic and multi-morbidity differences across the three DMT groups were statistically significant (p-value<0.05).

Two-tailed student's T-tests, with unequal variance[24] were used to assess the significance (p-value<0.05) of the disability and COI changes over time, within each treatment group, compared to baseline values (at diagnosis) and differences across the treatment groups.

A generalised linear model[25], with Poisson distribution for costs, was used to estimate mean cost per patient per year, and per group, and 95% CI of the estimation. The model was adjusted with the disability progression of PwMS over time, by setting the EDSS variable to the mean EDSS score across all the three groups for that year.

For peer review only - http://bmjopen.bmj.com/site/about/guidelines.xhtml

3 Results

In total, 3673 PwMS, receiving their first treatment with IFN (N=2696), GA (N=441), or NAT (N=536) between 2006-2015, were included. Sex, age distribution in the groups, educational level, family situation, country of birth, type of living area, and multi-morbidity (any) differed across the groups (p-value<0.05; Table 2). NAT had fewer people with any comorbidities (Table 2) vs. the IFN and GA groups. The baseline EDSS score was similar for the three groups (Supplementary Figure 1, in the Online Supplementary Material).

In Table 3, the unadjusted mean per patient per year COI for MS is presented, over the 10-year follow-up, and for the three treatment groups. Figures 1a and b show the disability adjusted healthcare costs and productivity losses. Disability adjusted costs were also computed for all the cost components (Supplementary Figure 2a-f, in the Online Supplementary Material).

People on NAT had higher healthcare costs vs. the IFN and GA groups (p-value<0.05 for both comparisons), driven by the higher drug costs for this group (Supplementary Figure 2d, in the Online Supplementary Material). IFN and GA groups had similar healthcare costs and productivity losses (p-values>0.05). Productivity losses for NAT vs. GA were similar (p-value>0.05), while higher when compared to those on IFN (p-value>0.05), driven by the higher SA costs for the NAT group (p-value=0.055 vs the IFN group). NAT had the lowest DP costs vs. the other two groups at the start of follow-up, and lower DP costs compared to those for the GA group (p-value>0.05) throughout the follow-up time.

For peer review only - http://bmjopen.bmj.com/site/about/guidelines.xhtml

Manuscript, Version 20220815 final draft

Table 2: Sociodemographic and multi-morbidity characteristics at year of diagnosis (index year)

	Treatment with Interferons N=2696 n(%)[a]	Treatment with Glatiramer Acetate N=441 n(%)[a]	Treatment with Natalizumab N=536 n(%)[a]	Pearson's Chi-Square (p-value)	Log-likelihood test Chi-Square (p-value)
**Sex**				7.49 (0.024)	7.70 (0.021)
Women	1882 (51.24)	333 (9.07)	364 (9.91)		
Men	814 (22.16)	108 (2.94)	172 (4.68)		
**Age at diagnosis (index year)**				107.26 (<.0001)	108.56 (<.0001)
20-25	321 (8.74)	45 (1.23)	114 (3.1)		
26-30	435 (11.84)	67 (1.82)	129 (3.51)		
31-35	465 (12.66)	65 (1.77)	114 (3.1)		
36-40	477 (12.99)	88 (2.4)	74 (2.01)		
41-45	463 (12.61)	75 (2.04)	46 (1.25)		
46-50	316 (8.6)	60 (1.63)	39 (1.06)		
51-55	219 (5.96)	41 (1.12)	20 (0.54)		
**Type of MS at diagnosis (index year)[b]**				2.24 (0.896)	2.25 (0.896)
RRMS	1960 (65.14)	302 (10.04)	419 (13.92)		
PPMS	23 (0.76)	4 (0.13)	4 (0.13)		
SPMS	187 (6.21)	31 (1.03)	33 (1.1)		
PRMS	31 (1.03)	6 (0.2)	9 (0.3)		
**Educational level[c]**				72.90 (<.0001)	58.09 (<.0001)
Elementary school	387 (10.84)	67 (1.88)	134 (3.75)		
High School	1290 (36.13)	210 (5.88)	225 (6.3)		
University/college	946 (26.5)	153 (4.29)	158 (4.43)		
**Country of birth[c]**				17.70 (0.0236)	14.70 (0.065)
Sweden	2348 (65.77)	390 (10.92)	454 (12.72)		
Nordic countries (except Sweden)	43 (1.2)	9 (0.25)	15 (0.42)		
EU27 (except Denmark, Finland, and Sweden)	42 (1.18)	8 (0.22)	16 (0.45)		
Rest of the world	190 (5.32)	23 (0.64)	32 (0.9)		
**Type of living area**				15.47 (0.004)	15.30 (0.004)
Big cities[c]	1020 (28.57)	142 (3.98)	210 (5.88)		
Medium sized cities	888 (24.87)	141 (3.95)	187 (5.24)		
Rural areas	715 (20.03)	147 (4.12)	120 (3.36)		
**Family situation[c]**				140.56 (<.0001)	123.74 (<.0001)

For peer review only - http://bmjopen.bmj.com/site/about/guidelines.xhtml

Married or cohabitant without children[d,e]	189 (5.29)	38 (1.06)	26 (0.73)		
Married or cohabitant with children[d,e]	1021 (28.6)	165 (4.62)	117 (3.28)		
Single without children[d,e]	1211 (33.92)	190 (5.32)	320 (8.96)		
Single with children[d,e]	174 (4.87)	28 (0.78)	25 (0.7)		
**Any multi-morbidity**				68.35 (<.0001)	53.92 (<.0001)
Yes	2580 (70.24)	424 (11.54)	466 (12.69)		
No	116 (3.16)	17 (0.46)	70 (1.91)		
**Anxiety/Depression**				4.10 (0.129)	3.91 (0.142)
Yes	137 (3.73)	28 (0.76)			
No	2559 (69.67)	413 (11.24)			

[a]The percentages are calculated as n divided with the total number of individuals in the study population (3673 if not otherwise indicated; see b)

[b]For 18% of the cohort, type of MS information was missing; so, information was available for 3009 PwMS in the study cohort

[c]The total number of individuals with this type of information in the study population (i.e., all individuals excluding those with missing information in this category) was 3570

[d]Only cohabitants with children in common are registered as cohabitants. Otherwise they were registered as single.

[e]As children were considered those <18 years living at home

For peer review only

Manuscript, Version 20220815 final draft

Table 3: Mean costs per patient per year, mean costs [95% confidence intervals] in € 2018 prices (crude observed means and confidence intervals, unadjusted), by treatment groups

	Year 0 (year of MS diagnosis)	Year 1	Year 2	Year 3	Year 4	Year 5	Year 6	Year 7	Year 8	Year 9	Year 10
**Interferon group**	**N=2696**	**N=2696**	**N=2644**	**N=2495**	**N=2219**	**N=1879**	**N=1556**	**N=1260**	**N=957**	**N=627**	**N=320**
Inpatient costs	1778 [1634-1921]	1081 [924-1238]	1330 [1149-1510]	1239 [1070-1407]	1150 [977-1324]	1243 [1024-1462]	1283 [1055-1512]	1084 [874-1293]	1442 [1006-1877]	1008 [708-1307]	1044 [609-1479]
Outpatient costs	1803 [1750-1857]	1371 [1315-1426]	1390 [1331-1448]	1420 [1355-1485]	1446 [1380-1512]	1531 [1456-1607]	1539 [1457-1622]	1629 [1513-1745]	1638 [1535-1741]	1729 [1597-1862]	1757 [1575-1938]
Co-payments	108 [106-109]	74 [72-75]	69 [67-71]	66 [64-68]	66 [64-68]	67 [65-69]	67 [64-69]	66 [64-69]	67 [64-70]	65 [62-69]	69 [63-74]
Drug costs	5915 [5743-6086]	11,191 [11,004-11,377]	11,049 [10,831-11,268]	10,863 [10,616-11,110]	10,706 [10,431-10,981]	10,131 [9823-10,440]	9868 [9522-10,215]	9378 [8991-9766]	8640 [8192-9089]	8410 [7849-8970]	7942 [7133-8751]
**Total healthcare costs**	**9603 [9362-9844]**	**13726 [13464-13988]**	**13838 [13537-14139]**	**13,587 [13,267-13,907]**	**13,369 [13,025-13,713]**	**12,972 [12,574-13,370]**	**12,758 [12,326-13,190]**	**12,157 [11,694-12,621]**	**11,787 [11,166-12,407]**	**11,212 [10,559-11,864]**	**10,812 [9910-11,714]**
SA costs	6940 [6481-7399]	7830 [7276-8384]	6598 [6090-7107]	5329 [4858-5801]	4658 [4175-5140]	4454 [3963-4946]	4120 [3602-4639]	3973 [3410-4537]	4418 [3695-5140]	3995 [3205-4786]	3903 [2842-4964]
DP costs	1893 [1558-2229]	2551 [2172-2931]	3894 [3439-4349]	5624 [5078-6170]	7399 [6743-8056]	8605 [7846-9365]	10,087 [9188-10,985]	11,966 [10,885-13,047]	12,805 [11,522-14,088]	14,819 [13,142-16,496]	15,232 [12,912-17,552]
**Total productivity losses**	**8834 [8288-9379]**	**10,375 [9727-11,024]**	**10,492 [9836-11,149]**	**10,954 [10,263-11,645]**	**12,057 [11,280-12,835]**	**13,060 [12,197-13,922]**	**14,207 [13,209-15,205]**	**15,939 [14,774-17,104]**	**17,223 [15,834-18,612]**	**18,814 [17,054-20,574]**	**19,135 [16,718-21,552]**
**Glatiramer Acetate group**	**N=441**	**N=441**	**N=430**	**N=406**	**N=348**	**N=292**	**N=225**	**N=170**	**N=116**	**N=66**	**N=31**
Inpatient costs	2221 [1736-2706]	1325 [863-1787]	1185 [777-1593]	1138 [733-1543]	1432 [958-1905]	1540 [912-2169]	881 [445-1317]	1403 [828-1978]	1327 [603-2051]	551 [134-968]	1419 [-913-3751]
Outpatient costs	2045 [1898-2193]	1509 [1363-1655]	1513 [1371-1654]	1569 [1409-1729]	1630 [1431-1829]	1719 [1508-1931]	1712 [1481-1943]	1737 [1478-1997]	1999 [1557-2440]	2169 [1576-2763]	2744 [1164-4324]
Co-payments	109 [104-113]	79 [74-84]	74 [69-78]	72 [67-77]	71 [66-77]	75 [69-81]	70 [64-77]	78 [69-86]	74 [64-84]	71 [60-82]	74 [57-90]
Drug costs	5680 [5237-6123]	10,310 [9821-10,800]	10,275 [9726-10,825]	9909 [9322-10,495]	9228 [8568-9887]	9043 [8272-9814]	8862 [7981-9744]	9298 [8258-10,339]	8405 [7177-9633]	8483 [6752-10,214]	8298 [5654-10,941]

For peer review only

For peer review only - http://bmjopen.bmj.com/site/about/guidelines.xhtml

**Total healthcare costs**	**10,055 [9358-10,753]**	**13,260 [12,521-13,998]**	**13,047 [12,274-13,819]**	**12,688 [11,937-13,439]**	**12,361 [11,504-13,217]**	**12,377 [11,336-13,418]**	**11,526 [10,483-12,570]**	**12,516 [11,288-13,744]**	**11,805 [10,308-13,302]**	**11,275 [9329-13,221]**	**12,534 [8618-16,450]**
SA costs	8133 [6860-9406]	8697 [7243-10151]	7203 [5844-8562]	6661 [5310-8013]	6116 [4726-7506]	5164 [3775-6553]	5163 [3634-6693]	5076 [3428-6725]	5201 [2902-7499]	4613 [2411-6815]	3822 [657-6988]
DP costs	3335 [2222-4449]	4135 [2901-5369]	5322 [3923-6721]	7629 [5956-9302]	9772 [7793-11,751]	11,810 [9491-14,129]	13,166 [10,490-15,841]	15,118 [11,794-18,441]	18,111 [13,852-22,370]	18,826 [13,266-24,386]	19,079 [11,725-26,433]
**Total productivity losses**	**11,469 [9889-13,048]**	**12,906 [11,124-14,688]**	**12,525 [10,710-14,340]**	**14,290 [12,294-16,287]**	**15,888 [13,645-18,131]**	**16,974 [14,468-19,480]**	**18,329 [15,425-21,233]**	**20,194 [16,778-23,610]**	**23,312 [18,854-27,770]**	**23,439 [17,705-29,172]**	**22,901 [15,146-30,656]**
**Natalizumab group**	**N=536**	**N=536**	**N=453**	**N=379**	**N=315**	**N=260**	**N=197**	**N=153**	**N=118**	**N=65**	**N=26**
Inpatient costs	4871 [4210-5531]	3236 [2524-3949]	2321 [1756-2886]	3049 [1687-4411]	2295 [1552-3037]	1729 [1076-2381]	1645 [922-2368]	1840 [1047-2633]	1089 [476-1703]	2956 [1097-4815]	3308 [166-6450]
Outpatient costs	2666 [2480-2853]	2585 [2377-2793]	2256 [2041-2471]	2165 [1921-2408]	2496 [2192-2801]	2258 [2011-2506]	2207 [1907-2506]	2402 [2010-2794]	2231 [1856-2605]	2836 [2242-3431]	3033 [1934-4132]
Co-payments	135 [131-140]	102 [97-107]	87 [83-92]	87 [82-92]	88 [82-94]	90 [84-96]	82 [75-89]	86 [78-95]	78 [70-86]	92 [77-107]	94 [71-118]
Drug costs	7582 [7077-8087]	16,052 [15,514-16,591]	15,959 [15,319-16,599]	14,577 [13,795-15,358]	13,537 [12,642-14,432]	13,319 [12,300-14,339]	13,392 [12,236-14,548]	11,848 [10,488-13,208]	11,357 [9834-12,880]	11,218 [9015-13,421]	13,411 [8179-18,643]
**Total healthcare costs**	**15,254 [14,309-16,199]**	**22,629 [21,731-23,527]**	**20,623 [19,760-21,487]**	**19,877 [18,253-21,501]**	**18,416 [17,236-19,596]**	**17,396 [16,270-18,523]**	**17,325 [16,043-18,607]**	**16,176 [14,631-17,722]**	**14,755 [12,933-16,576]**	**17,103 [14,031-20,174]**	**19,846 [13,308-26,384]**
SA costs	9701 [8507-10895]	11,877 [10331-13,423]	9885 [8373-11,397]	7766 [6317-9214]	7497 [5794-9200]	7036 [5319-8753]	5302 [3645-6959]	5412 [3581-7243]	4797 [2812-6782]	5353 [2252-8453]	3323 [287-6359]
DP costs	2331 [1500-3163]	3398 [2392-4404]	5043 [3734-6352]	7643 [5953-9334]	9256 [7254-11,258]	12,084 [9647-14,522]	14,329 [11,450-17,209]	15,902 [12,344-19,460]	17,996 [13,719-22,274]	19,957 [13,881-26,034]	20,670 [10,842-30,499]
**Total productivity losses**	**12,032 [10,666-13,399]**	**15,269 [13,511-17,027]**	**14,929 [13,071-16,786]**	**15,409 [13,368-17,450]**	**16,753 [14,381-19,126]**	**19,120 [16,457-21,783]**	**19,632 [16,589-22,674]**	**21,314 [17,652-24,976]**	**22,794 [18,506-27,082]**	**25,310 [19,219-31,401]**	**23,994 [14,434-33,553]**

For peer review only

For peer review only - http://bmjopen.bmj.com/site/about/guidelines.xhtml

4 Discussion

In this register-based prospective cohort study we explored the development of COI in MS in Sweden over time, among newly diagnosed PwMS, in relation to what type of first line treatment was used; IFN, vs GA, vs NAT, adjusting for MS disability progression. The analysis sub-groups were constructed based on the DMTs that were licensed to be used as 1st line therapies for MS in Sweden during 2006 and 2016, and for which we had information during the study's data period (2006-2016).

Overall, the general trends of healthcare costs and productivity losses that were observed in this study, i.e., decreasing healthcare costs and increasing productivity losses, are in line with previous COI studies in MS with a similar longitudinal design[26, 27]. No significant differences in healthcare costs were observed between the IFN and GA, while NAT had higher healthcare costs (p-value<0.05). This could be explained by the higher treatment cost with NAT[28], i.e., higher drug cost and healthcare utilization to administer this DMT. As previous studies have shown, in the last two decades, the cost of drugs has become main COI driver in MS[4].

In addition, outpatient costs for PwMS on NAT could have played a role; they were higher for NAT (p-value>0.05). For the beginning of follow-up, until PwMS on NAT change therapy, this could be explained by that fact that these patients require at least one extra outpatient visit per month to get their treatment[29]. Before treatment, additional diagnostic tests (blood tests and in cases MRI scans) have to be performed, not needed for PwMS on other therapies, including IFN and GA[29]. Therefore, when compared to IFN and GA, healthcare costs for NAT could be amplified due to the extra outpatient visits needed for the treatment itself. Had no such visits and tests been necessary, outpatient costs NAT could have been at the same level as the ones for IFN and GA, or even lower.

For peer review only - http://bmjopen.bmj.com/site/about/guidelines.xhtml

Towards the end of follow-up, when people may have switched to other DMTs, or stopped treatment, other factors could be associated with the observed healthcare cost differences, which have not been covered in this study, such as the progression of MS symptoms, relapses, and multi-morbidity.

Productivity losses for NAT and GA were similar; both had higher productivity losses vs. IFN (p-value<0.05). Since the comparative effectiveness of IFN and GA is similar[30], other unmeasured factors than disease progression, such as fatigue and multi-morbidity could explain this finding. On the other hand, even if treatment with NAT can lead to lower relapse rates[31], and hence possibly to slower disease progression, compared to IFN and GA, productivity losses for this group were higher. The need to take time off work when on NAT related to the administration of the therapy (outpatient visits to administer the therapy and run diagnostic tests), and recovery from the administration of this DMT, could explain part of this observed outcome[29], since it adds extra SA costs for NAT[32].

In fact, only SA costs were higher for NAT compared to the other groups; DP costs were lower than GA, indicating that even if PwMS receiving NAT as first-line treatment, are having higher MS disability at baseline, and probably higher disease activity compared to those receiving IFN or GA[29], still, their ability to maintain their long-term work capacity is higher. Experiencing less relapses and in general less MS symptoms like fatigue, which can allow patients to maintain employment longer[33].

Health economic models published previously, presenting the cost progression related to these three DMTs, have often been criticised due to their limited data availability as well as the methodological complexities and uncertainties[33]. The COI of MS associated with new generation therapies like NAT, compared to older therapies (IFN and GA) has not been studied so far

For peer review only - http://bmjopen.bmj.com/site/about/guidelines.xhtml

involving longitudinal COI data with multiple years of follow-up for costs and disease progression.

4.1 4.1 Strengths and limitations

Main strengths of this study are its prospective cohort design, with several years of follow-up, and that data from administrative registers could be used, instead of self-reported information. All PwMS in the SMSreg diagnosed with MS between 2006 and 2015 and receiving first-line therapy with NAT, IFN, or GA could be followed for several years in nationwide registers, eliminating recall biases when using self-reported information from PwMS[4]. While this is an important strength, we did not have information from nationwide registers in Sweden regarding primary healthcare, rehabilitation, home help, home investments to improve mobility, and other out of pocket expenses, to include in our COI calculations. Such information could complement our results, showing how MS symptoms and multi-morbidity can impact the overall healthcare costs in MS, based on the DMT treatment administered.

In addition, the longest follow-up time we had was 10 years after diagnosis; while this time can give important information regarding the COI progression for the three treatment groups, only very few patients had this number of follow-up years. Therefore, the longer the follow-up the higher the uncertainty for the measured COI is. Moreover, while the impact of the first-line therapy on the COI can be observed for the time PwMS are receiving that treatment and maybe for a short period of time afterwards, also other factors could be strongly associated with the COI progression over time after the first-line therapy was stopped, not measured in this study (e.g., type of second-line therapy, adherence to treatment, etc.).

Another important advantage is that robust information on productivity losses was available, which are the main long-term driver of the COI in MS as shown in this and previous studies [4], enabling us to apply the societal perspective on the COI calculations. However, short-

For peer review only - http://bmjopen.bmj.com/site/about/guidelines.xhtml

term SA-spells of 14 days or less, were not quantified into productivity losses, possibly leading to their underestimation. Short-term SA spells have not been considered for both employed (including self-employed) and unemployed patients, hence no bias based on employment status has been introduced in the calculation of productivity losses.

Moreover, any productivity reductions while being present at work that could potentially be related to the presence of MS were not quantified. Furthermore, productivity losses incurred by partners of people with MS, i.e., informal care costs, were not included in the COI calculations; while measuring these costs was beyond the aim of this study, they are an important cost component when defining the overall COI of MS[4].

Similar to what previous COI in MS studies have done[4], we used the EDSS score to explore disability progression for PwMS in the three treatment groups. However, when EDSS information was missing, information had to be imputed ; this has likely diluted the EDSS score used in the adjusted COI analysis. In addition, EDSS has been primarily developed and used for research purposes, not for clinical or treatment decisions in MS, and its scores are less effectively tracking disease progression over time[34]. Also, some MS symptoms, like fatigue, which are important and can limit the everyday activities of PwMS, are not well detailed in the EDSS assessment[34], obtaining in this way a skewed result regarding the disability of PwMS. Additional clinical measures could have complemented our analysis.

Another limitation is that the generalizability of our findings can be limited. The study cohort was taken from SMSreg, which back in 2006 had approximately 50% coverage of all PwMS in Sweden, and 80% in 2015[35]. Assuming no distinct differences in the sociodemographic characteristics, multi-morbidity, and disability with the PwMS that our study did not include, our findings could be generalizable to all PwMS in Sweden. Generalization of our findings to other

For peer review only - http://bmjopen.bmj.com/site/about/guidelines.xhtml

countries is not possible, considering the differences in the organisation of healthcare and social security systems.

5 Conclusions

Using real-world data from Swedish nationwide registers, this study explored the association between the type of first-line DMT received (IFN vs. GA vs. NAT) with COI progression, indicating that PwMS on NAT maintained their work capacity for a longer time, leading to lower DP costs. COI serves as an objective measure showing the importance of the use of second-generation treatments in maintaining at low levels the progression of MS over time, and hence the overall COI.

For peer review only - http://bmjopen.bmj.com/site/about/guidelines.xhtml

Data Availability Statement

The data used in this study is administered by the Division of Insurance Medicine, Karolinska Institutet, and cannot be made publicly available. According to the General Data Protection Regulation, the Swedish law SFS 2018:218, the Swedish Data Protection Act, the Swedish Ethical Review Act, and the Public Access to Information and Secrecy Act, these type of sensitive data can only be made available, after legal review, for researchers who meet the criteria for access to this type of sensitive and confidential data. Readers may contact Professor Kristina Alexanderson (kristina.alexanderson@ki.se) regarding the data.

Author Contributions

All authors (Korinna Karampampa, KK; Hanna Gyllensten, HG; Emilie Friberg, EF; Chantelle Murley, CM; Jan Hillert, JH; Andrius Kavaliunas, AK; Tomas Olsson, TO; Kristina Alexanderson, KA) contributed to the conceptualization of the research questions, the study design and methods. KK performed the analysis of the data, interpreted the study findings, and drafted the manuscript. All remaining authors (HG, EF, CM, JH, AK, TO, KA) assisted in the interpretation of the study findings and contributed with comments/suggestions and text to the manuscript.

Compliance with Ethical Standards

Patient consent for publication: Not required.

Ethics approval Due to the use of pseudonymised data from total population registers, informed consent from the research participants was not applicable. The project received ethical approval from the Regional Ethical Review Board in Stockholm. Approval numbers: 2007/762-31; 2009/23-32; 2009/1917-32; 2010/466-32; 2011/806-32; 2011/1710-32; and 2014/236-32.

For peer review only

Funding: This study was supported by an unrestricted investigator-initiated trial grant from Biogen to support the conduct of this study (Award number N/A). The study also had financial support from the Swedish Research Council (Awand number N/A).

Conflicts of Interest: All authors (KK, HG, EF, CM, JH, AK, TO, KA) are employed or affiliated at the Department of Clinical Neuroscience, Karolinska Institutet, Stockholm, Sweden. KK is currently employed by Celgene/Bristol Myers Squibb; she initiated this study while being employed at Karolinska Institutet (employment ended in October 2019); since then, she has received no salary from Karolinska Institutet or other type of funding for this research. HG is currently employed part-time by Statfinn/EPID Research (which is part of IQVIA); both companies are contract research organizations that perform commissioned pharmacoepidemiological studies, and therefore are collaborating with several pharmaceutical companies. AK is currently also employed by Takeda Pharma AB. JH, KA, and EF are collaborating with several pharmaceutical companies; EF has received an unrestricted MS research grant from Celgene.TO has received advisory board and/or lecture honoraria, and unrestricted MS research grants from Biogen, Novartis, Sanofi, Merck and Roche.

For peer review only

References

1. Dutta R and Trapp BD. Mechanisms of neuronal dysfunction and degeneration in multiple sclerosis. *Prog Neurobiol*. 2011; 93: 1-12.
2. Ahlgren C, Oden A and Lycke J. High nationwide incidence of multiple sclerosis in Sweden. *PLoS One*. 2014; 9: e108599.
3. Gyllensten H, Wiberg M, Alexanderson K, et al. Costs of illness of multiple sclerosis in Sweden: a population-based register study of people of working age. *Eur J Health Econ*. 2017.
4. Ernstsson O, Gyllensten H, Alexanderson K, Tinghög P, Friberg E and Norlund A. Cost of Illness of Multiple Sclerosis - A Systematic Review. *PLoS One*. 2016; 11: e0159129.
5. Cerqueira JJ, Compston DAS, Geraldes R, et al. Time matters in multiple sclerosis: can early treatment and long-term follow-up ensure everyone benefits from the latest advances in multiple sclerosis? *J Neurol Neurosurg Psychiatry*. 2018; 89: 844-50.
6. Iannazzo S, Iliza AC and Perrault L. Disease-Modifying Therapies for Multiple Sclerosis: A Systematic Literature Review of Cost-Effectiveness Studies. *Pharmacoeconomics*. 2018; 36: 189-204.
7. Hillert J and Stawiarz L. The Swedish MS registry - clinical support tool and scientific resource. *Acta neurologica Scandinavica*. 2015; 132: 11-9.
8. Ludvigsson JF, Almqvist C, Bonamy AK, et al. Registers of the Swedish total population and their use in medical research. *Eur J Epidemiol*. 2016; 31: 125-36.
9. Pratt NL, Kerr M, Barratt JD, et al. The validity of the Rx-Risk Comorbidity Index using medicines mapped to the Anatomical Therapeutic Chemical (ATC) Classification System. *BMJ Open*. 2018; 8: e021122.
10. Collins CD, Ivry B, Bowen JD, et al. A comparative analysis of Patient-Reported Expanded Disability Status Scale tools. *Mult Scler*. 2016; 22: 1349-58.
11. Goodkin DE. EDSS reliability. *Neurology*. 1991; 41: 332.
12. Drummond MF, Sculpher MJ, Claxton K, Stoddart GL and Torrance GW. *Methods for the economic evaluation of health care programmes*. Oxford university press, 2015.
13. National Insurance Agency [Försäkringskassan]. Social Insurance in Figures 2017. 2017.
14. Eurostat. HICP - all items - annual average indices (2015=100). https://ec.europa.eu/eurostat/tgm/table.do?tab=table&init=1&language=en&pcode=tec00027&plugin=12019.
15. Swedish Association of Local Authorities and Regions [Sveriges Kommuner och Landsting]. Economic statistics - KPP Somatik - SKL. https://skl.se/ekonomijuridikstatistik/statistik/kostnadperpatientkpp/kppsomatik.1077.html2019.
16. Swedish Association of Local Authorities and Regions [Sveriges Kommuner och Landsting]. Economic statistics - Patientavgifter fr.o.m. den 1 januari 2018. https://skl.se/download/18.3ee4cc4f1611af77b89179dd/1517495154691/avgift_%C3%B6ppenv%C3%A5rd_inkl.missiv_slutenavg18_19jan18.pdf2018.
17. Vårdguiden 1177. Patient fees in Västra Götaland county in Sweden [Patientavgifter i Västra Götaland]. https://www.1177.se/Vastra-Gotaland/Regler-och-rattigheter/Patientavgifter-i-Vastra-Gotaland/2018.
18. FASS.se. Tysabri. https://www.fass.se/LIF/product?nplId=20040916001115&userType=0&docType=6&scrollPosition=751.33331298828122019.
19. Salzer J, Svenningsson R, Alping P, et al. Rituximab in multiple sclerosis: A retrospective observational study on safety and efficacy. *Neurology*. 2016; 87: 2074-81.

For peer review only - http://bmjopen.bmj.com/site/about/guidelines.xhtml

20. FASS.se. Rituximab - price. https://www.fass.se/LIF/product?userType=2&nplId=19980602000029&docType=30&scrollPosition=600s2019.
21. Statistics Sweden. Genomsnittlig månadslön 1973–2018. https://www.scb.se/hitta-statistik/statistik-efter-amne/arbetsmarknad/loner-och-arbetskostnader/lonestrukturstatistik-privat-sektor-slp/pong/tabell-och-diagram/genomsnittlig-manadslon-1973-/2019.
22. Swedish Tax Authority [Skatteverket]. Belopp och procent för åren 2019-2004 - privat. https://www.skatteverket.se/privat/skatter/beloppochprocent.4.18e1b10334ebe8bc80004109.html2019.
23. Moore DS. Tests of chi-squared type. *Goodness-of-fit-techniques*. Routledge, 2017, p. 63-96.
24. Kim TK. T test as a parametric statistic. *Korean journal of anesthesiology*. 2015; 68: 540-6.
25. McCullagh P and Nelder J. Generalized linear models., 2nd edn.(Chapman and Hall: London). *Standard book on generalized linear models*. 1989.
26. Gyllensten H, Kavaliunas A, Murley C, et al. Costs of illness progression for different multiple sclerosis phenotypes: A population-based study in Sweden. *Submitted*. 2018.
27. Karampampa K, Gyllensten H, Yang F, et al. Healthcare, Sickness Absence, and Disability Pension Cost Trajectories in the First 5 Years After Diagnosis with Multiple Sclerosis: A Prospective Register-Based Cohort Study in Sweden. *Pharmacoecon Open*. 2019.
28. Hartung DM, Bourdette DN, Ahmed SM and Whitham RH. The cost of multiple sclerosis drugs in the US and the pharmaceutical industry: Too big to fail? *Neurology*. 2015; 84: 2185-92.
29. Piehl F, Olsson T, Lycke J, et al. Läkemedelsbehandling av multipel skleros (MS)– bakgrundsdokumentation. https://lakemedelsverket.se/upload/halso-och-sjukvard/behandlingsrekommendationer/bakg_dok/Lakemedelsbehandling_av_multipel_skleros_MS_bakgrundsdokumentation_webb.pdf.
30. Melendez-Torres GJ, Armoiry X, Court R, et al. Comparative effectiveness of beta-interferons and glatiramer acetate for relapsing-remitting multiple sclerosis: systematic review and network meta-analysis of trials including recommended dosages. *BMC Neurol*. 2018; 18: 162.
31. Spelman T, Kalincik T, Jokubaitis V, et al. Comparative efficacy of first-line natalizumab vs IFN-beta or glatiramer acetate in relapsing MS. *Neurol Clin Pract*. 2016; 6: 102-15.
32. Kwiatkowski A, Marissal JP, Pouyfaucon M, Vermersch P, Hautecoeur P and Dervaux B. Social participation in patients with multiple sclerosis: correlations between disability and economic burden. *BMC Neurol*. 2014; 14: 115.
33. Glanz BI, Degano IR, Rintell DJ, Chitnis T, Weiner HL and Healy BC. Work productivity in relapsing multiple sclerosis: associations with disability, depression, fatigue, anxiety, cognition, and health-related quality of life. *Value Health*. 2012; 15: 1029-35.
34. Meyer-Moock S, Feng YS, Maeurer M, Dippel FW and Kohlmann T. Systematic literature review and validity evaluation of the Expanded Disability Status Scale (EDSS) and the Multiple Sclerosis Functional Composite (MSFC) in patients with multiple sclerosis. *BMC Neurol*. 2014; 14: 58.
35. Swedish Neuroregistries MS. Visualisation and Analysis platform (VAP) for Multiple Sclerosis - Coverage ratio broken down by county, gender and the last follow-up date [Täckningsgrad uppdelat för län, kön och sista uppföljningsdatum], Coverage ratio against the patient register, by year [Täckningsgrad mot patientregistret, år].

For peer review only - http://bmjopen.bmj.com/site/about/guidelines.xhtml

https://www.neuroreg.se/en.html/multiple-sclerosis-realtime-data-and-results-vap: Swedish Neuroregistries - Multiple Sclerosis, 2019.

For peer review only - http://bmjopen.bmj.com/site/about/guidelines.xhtml

Figure 1(A,B)

Healthcare costs (A) and Productivity losses (B) (estimated mean from the regression) from baseline (year at MS diagnosis) to the end of follow-up, by treatment groups, adjusted for disability progression during the follow-up

For peer review only

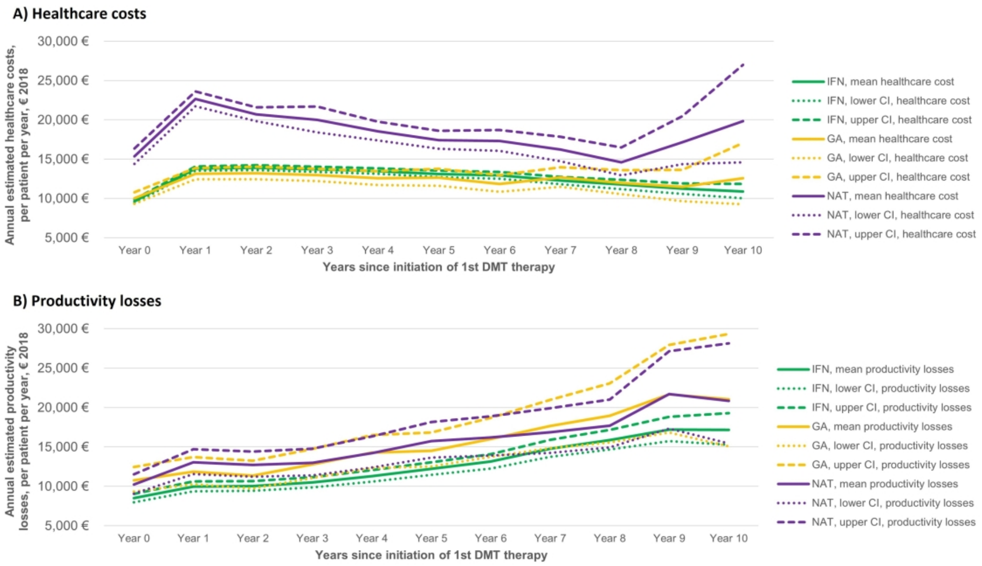

Healthcare costs (A) and Productivity losses (B) (estimated mean from the regression) from baseline (year at MS diagnosis) to the end of follow-up, by treatment groups, adjusted for disability progression during the follow-up

294x170mm (300 x 300 DPI)

Online Supplementary Material

Supplementary Figure 1: EDSS score progression from baseline (index year) to the end of follow-up, by IFN vs GA vs NAT treatment groups

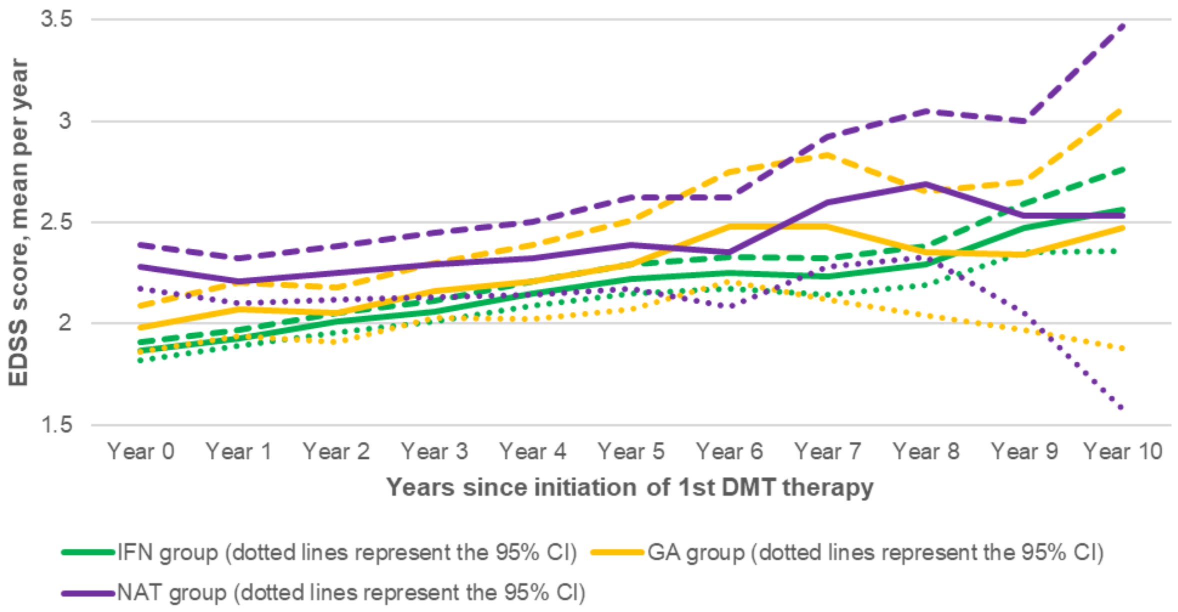

review only

Supplementary Figure 2 (a-f): Progression (estimated mean from the regression) for all cost components, from baseline (year at MS diagnosis) to the end of follow-up, by treatment groups, adjusted for disability progression during follow-up

A) Inpatient costs

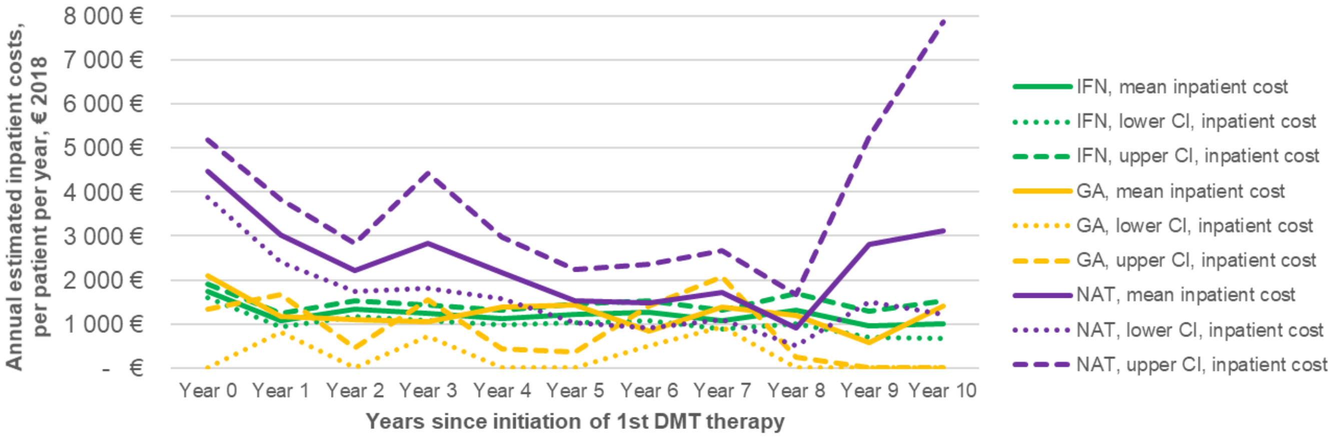

B) Outpatient costs

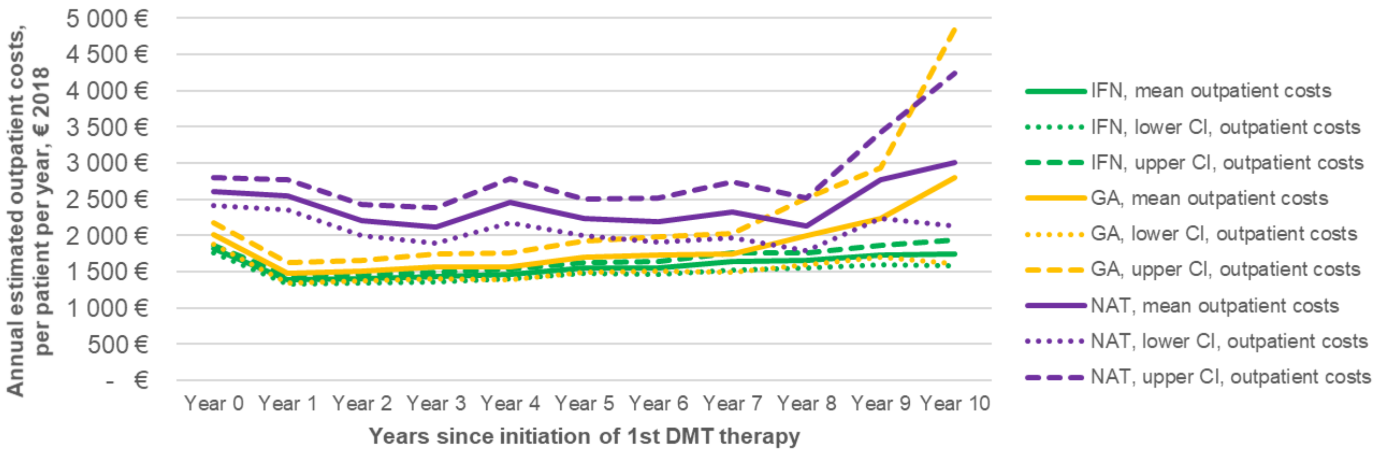

C) Co-payments

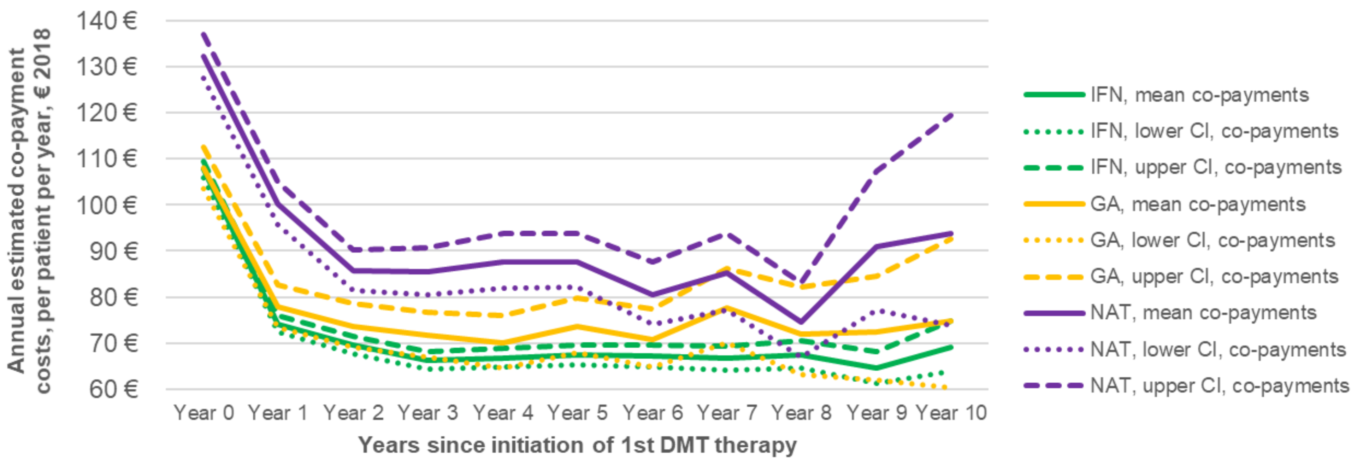

D) Drug costs

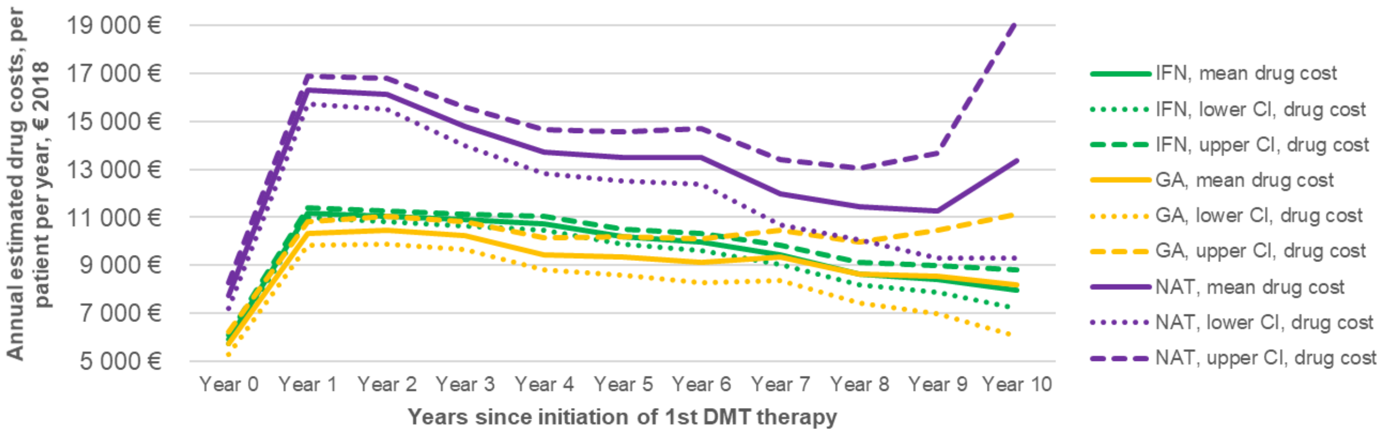

E) Sickness absence costs

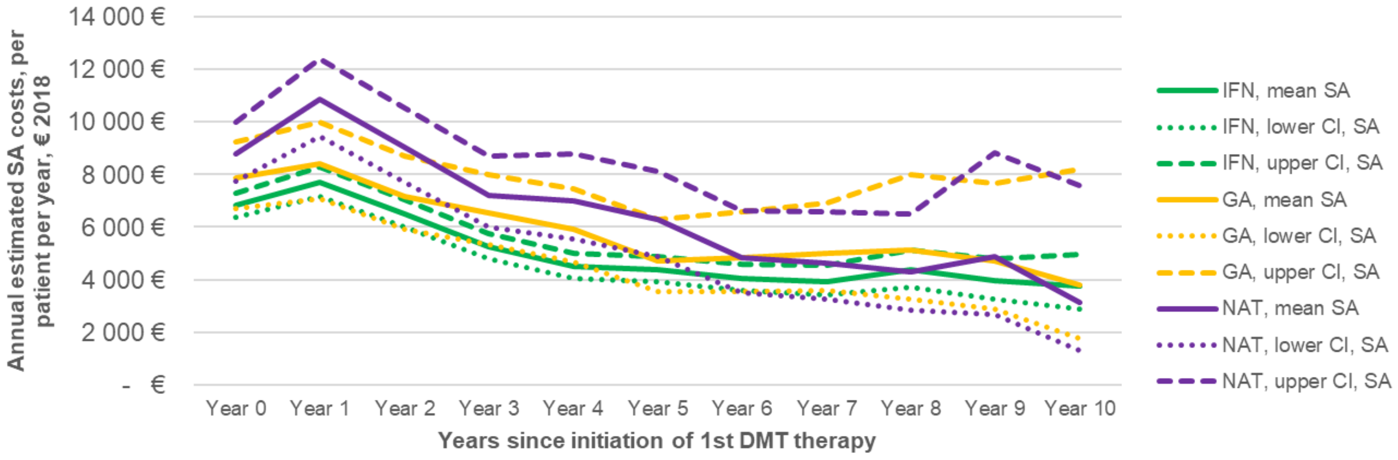

F) Disability pension costs

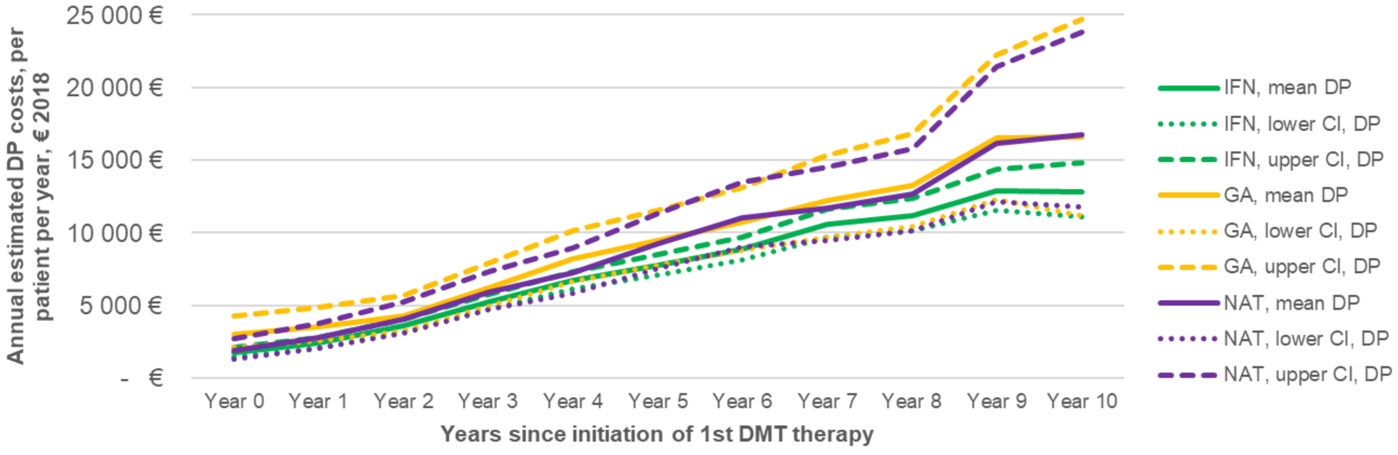

STROBE Statement—Checklist of items that should be included in reports of ***cohort studies***

	Item No	Recommendation	Page No
**Title and abstract**	1	(*a*) Indicate the study's design with a commonly used term in the title or the abstract	1
		(*b*) Provide in the abstract an informative and balanced summary of what was done and what was found	2
**Introduction**			
Background/rationale	2	Explain the scientific background and rationale for the investigation being reported	4
Objectives	3	State specific objectives, including any prespecified hypotheses	4
**Methods**			
Study design	4	Present key elements of study design early in the paper	5
Setting	5	Describe the setting, locations, and relevant dates, including periods of recruitment, exposure, follow-up, and data collection	5-6
Participants	6	(*a*) Give the eligibility criteria, and the sources and methods of selection of participants. Describe methods of follow-up	5-6
		(*b*) For matched studies, give matching criteria and number of exposed and unexposed	n.a.
Variables	7	Clearly define all outcomes, exposures, predictors, potential confounders, and effect modifiers. Give diagnostic criteria, if applicable	5-9
Data sources/ measurement	8*	For each variable of interest, give sources of data and details of methods of assessment (measurement). Describe comparability of assessment methods if there is more than one group	5-7
Bias	9	Describe any efforts to address potential sources of bias	5-7
Study size	10	Explain how the study size was arrived at	5
Quantitative variables	11	Explain how quantitative variables were handled in the analyses. If applicable, describe which groupings were chosen and why	8-9
Statistical methods	12	(*a*) Describe all statistical methods, including those used to control for confounding	8-9
		(*b*) Describe any methods used to examine subgroups and interactions	8-9
		(*c*) Explain how missing data were addressed	7-9
		(*d*) If applicable, explain how loss to follow-up was addressed	6-9
		(*e*) Describe any sensitivity analyses	n.a.
**Results**			
Participants	13*	(a) Report numbers of individuals at each stage of study—eg numbers potentially eligible, examined for eligibility, confirmed eligible, included in the study, completing follow-up, and analysed	10
		(b) Give reasons for non-participation at each stage	5-10
		(c) Consider use of a flow diagram	n.a.
Descriptive data	14*	(a) Give characteristics of study participants (eg demographic, clinical, social) and information on exposures and potential confounders	10
		(b) Indicate number of participants with missing data for each variable of interest	10
		(c) Summarise follow-up time (eg, average and total amount)	10
Outcome data	15*	Report numbers of outcome events or summary measures over time	10

For peer review only - http://bmjopen.bmj.com/site/about/guidelines.xhtml

Main results	16	(*a*) Give unadjusted estimates and, if applicable, confounder-adjusted estimates and their precision (eg, 95% confidence interval). Make clear which confounders were adjusted for and why they were included	10, 21-26
		(*b*) Report category boundaries when continuous variables were categorized	n.a.
		(*c*) If relevant, consider translating estimates of relative risk into absolute risk for a meaningful time period	n.a.
Other analyses	17	Report other analyses done—eg analyses of subgroups and interactions, and sensitivity analyses	10, 21-26
**Discussion**			
Key results	18	Summarise key results with reference to study objectives	11-15
Limitations	19	Discuss limitations of the study, taking into account sources of potential bias or imprecision. Discuss both direction and magnitude of any potential bias	13-15
Interpretation	20	Give a cautious overall interpretation of results considering objectives, limitations, multiplicity of analyses, results from similar studies, and other relevant evidence	11-15
Generalisability	21	Discuss the generalisability (external validity) of the study results	14-15
**Other information**			
Funding	22	Give the source of funding and the role of the funders for the present study and, if applicable, for the original study on which the present article is based	16-17

*Give information separately for exposed and unexposed groups.

Note: An Explanation and Elaboration article discusses each checklist item and gives methodological background and published examples of transparent reporting. The STROBE checklist is best used in conjunction with this article (freely available on the Web sites of PLoS Medicine at http://www.plosmedicine.org/, Annals of Internal Medicine at http://www.annals.org/, and Epidemiology at http://www.epidem.com/). Information on the STROBE Initiative is available at http://www.strobe-statement.org.

For peer review only

For peer review only - http://bmjopen.bmj.com/site/about/guidelines.xhtml

Cost-of-illness progression among people newly diagnosed with multiple sclerosis: a cohort study regarding treatment initiation with interferons, glatiramer acetate, or natalizumab

Journal:	*BMJ Open*
Manuscript ID	bmjopen-2022-067516.R1
Article Type:	Original research
Date Submitted by the Author:	21-Mar-2023
Complete List of Authors:	Karampampa, Korinna; Karolinska Institutet, Department of Clinical Neuroscience<br>Gyllensten, Hanna; University of Gothenburg, Institute of Health and Care Sciences; University of Gothenburg Centre for Person-Centred Care,<br>Friberg, Emilie; Karolinska Institute<br>Murley, Chantelle; Karolinska Institutet Department of Clinical Neuroscience, Clinical Neuroscience<br>Kavaliunas, A; Karolinska Universitetssjukhuset<br>Hillert, Jan; Karolinska Institute, Clinical Neuroscience<br>Olsson, Tomas; Karolinska Institute<br>Alexanderson, Kristina; karolinska institutet, Department of Clinical Neuroscience
**Primary Subject Heading**:	Health economics
Secondary Subject Heading:	Neurology, Epidemiology
Keywords:	Multiple sclerosis < NEUROLOGY, HEALTH ECONOMICS, Health economics < HEALTH SERVICES ADMINISTRATION & MANAGEMENT

SCHOLARONE™
Manuscripts

I, the Submitting Author has the right to grant and does grant on behalf of all authors of the Work (as defined in the below author licence), an exclusive licence and/or a non-exclusive licence for contributions from authors who are: i) UK Crown employees; ii) where BMJ has agreed a CC-BY licence shall apply, and/or iii) in accordance with the terms applicable for US Federal Government officers or employees acting as part of their official duties; on a worldwide, perpetual, irrevocable, royalty-free basis to BMJ Publishing Group Ltd ("BMJ") its licensees and where the relevant Journal is co-owned by BMJ to the co-owners of the Journal, to publish the Work in this journal and any other BMJ products and to exploit all rights, as set out in our licence.

The Submitting Author accepts and understands that any supply made under these terms is made by BMJ to the Submitting Author unless you are acting as an employee on behalf of your employer or a postgraduate student of an affiliated institution which is paying any applicable article publishing charge ("APC") for Open Access articles. Where the Submitting Author wishes to make the Work available on an Open Access basis (and intends to pay the relevant APC), the terms of reuse of such Open Access shall be governed by a Creative Commons licence – details of these licences and which Creative Commons licence will apply to this Work are set out in our licence referred to above.

Other than as permitted in any relevant BMJ Author's Self Archiving Policies, I confirm this Work has not been accepted for publication elsewhere, is not being considered for publication elsewhere and does not duplicate material already published. I confirm all authors consent to publication of this Work and authorise the granting of this licence.

For peer review only

Cost-of-illness progression among people newly diagnosed with multiple sclerosis: a cohort study regarding treatment initiation with interferons, glatiramer acetate, or natalizumab

Authors: Korinna Karampampa[1], Hanna Gyllensten[2], Emilie Friberg[1], Chantelle Murley[1], , Andrius Kavaliunas[1], Jan Hillert[1],Tomas Olsson[1], Kristina Alexanderson[1]

Affiliations:

[1]Department of Clinical Neuroscience, Karolinska Institutet, SE-171 77 Stockholm, Sweden

[2]Institute of Health and Care Science, University of Gothenburg, Gothenburg, Sweden

Corresponding author:

Korinna Karampampa, MSc, PhD

Division of Insurance Medicine, Department of Clinical Neuroscience

Karolinska Institutet, SE-171 77 Stockholm, Sweden

E-mail: korinna.karampampa@ki.se

Phone: +46 704 33 74 15

For peer review only

For peer review only - http://bmjopen.bmj.com/site/about/guidelines.xhtml

ABSTRACT

Objectives: Disease modifying therapies (DMTs) can slow disease progression in multiple sclerosis (MS). The objective of this study was to assess their impact on the cost-of-illness (COI) progression in MS.

Setting: An observational study utilizing data from nationwide registers in Sweden.

Participants: People with MS (PwMS) in Sweden first diagnosed in 2006-2015, when aged 20-55, receiving first-line therapy with interferons (IFN), glatiramer acetate (GA), or natalizumab (NAT). They were followed up through 2016.

Primary and secondary outcome measures: Outcomes (in Euros, €) were: 1) secondary healthcare costs: specialized outpatient and inpatient care including out-of-pocket expenditure, DMTs including hospital administered MS therapies, and prescribed drugs, and 2) productivity losses: sickness absence and disability pension. Descriptive statistics and Poisson regression were computed, adjusting for disability progression using the Expanded Disability Status Scale.

Results: 3673 newly diagnosed PwMS who were treated with IFN (N=2696), GA (N=441), or NAT (N=536) were identified. Healthcare costs were similar for the INF and GA groups, while the NAT group had higher costs (p-value<0.05), owing to DMT and outpatient costs. IFN had lower productivity losses than NAT and GA (p-value>0.05), driven by fewer sickness absence days. NAT had a trend towards lower disability pension costs compared to GA (p-value>0.05).

Conclusions: Similar trends over time for healthcare costs and productivity losses were identified across the DMT sub-groups. PwMS on NAT maintained their work capacity for a longer time compared to those on GA, potentially leading to lower disability pension costs over time. COI serves as an objective measure to explore the importance of DMTs in maintaining low levels of progression of MS over time.

For peer review only - http://bmjopen.bmj.com/site/about/guidelines.xhtml

Strengths and limitations of this study:

- Data from nationwide registers with nationwide coverage were used, preventing recall and selection bias in the productivity loss and healthcare cost calculations.
- Additional costs (primary healthcare, rehabilitation, home help, home investments to improve mobility, other out-of-pocket expenses, sickness absence spells of 14 days or less, productivity reductions while being present at work, and informal care costs) were unable to be included in our COI calculations due to lack of register information.
- Factors which could be potentially strongly associated with the COI progression over time after the first-line therapy was stopped were not measured in this study (e.g., type of second-line therapy, adherence to treatment, etc.).
- While Expanded Disability Status Scale (EDSS) scores were used to adjust the COI progression for imbalances in disability progression across the three treatment groups as per established methodology, EDSS does not capture all relevant MS symptoms that can limit everyday activities, such as fatigue.

For peer review only - http://bmjopen.bmj.com/site/about/guidelines.xhtml

1 Introduction

Multiple sclerosis (MS) is a highly disabling chronic neurological disease[1], usually diagnosed when aged 20-40 years[1]. Sweden has one of the highest prevalence of MS in Europe, estimated at 189 cases over 100,000 individuals[2].

In 2010, the annual cost of illness (COI) of MS in Sweden, from a societal perspective, was approximately EUR 414 million[3]. Productivity losses[3] and the cost of disease-modifying therapies (DMT) were the two main cost drivers identified[4].

The COI of MS has been shown to increase with disease's disability progression[4]. On the other hand, treatment with DMTs, has been associated with slowing this progression; plausibly also reducing the increase in the COI in MS over time[5].

Until now, only a few observational studies have been conducted evaluating the COI progression in MS in relation to DMTs. However, these studies do not have a longitudinal design[6], and have also not accounted for disability progression.

The aim of this study was to explore the COI progression in MS among newly diagnosed people with MS (PwMS) in Sweden, in relation to the first DMT received (interferons (IFN) vs. glatiramer acetate (GA) vs. natalizumab (NAT)), adjusting for disability progression over time.

For peer review only - http://bmjopen.bmj.com/site/about/guidelines.xhtml

2 Methods

This was a register-based prospective cohort study of PwMS in Sweden who fulfilled the following two inclusion criteria:

- according to the Swedish MS register (SMSreg)[7] had their first MS diagnosis in one of the years 2006-2015 (index year), when aged 20-55, and lived in Sweden when first diagnosed, (maximum age of 55 was chosen to allow follow-up until ordinary retirement age of 65)
- received IFN, GA, or NAT (DMTs licensed for MS treatment in Sweden during the study period), within 1 month after diagnosis, and they stayed on the therapy for at least 6 months.

Never-users of DMTs, those not on IFN/GA/NAT within 1 month from MS diagnosis, and/or not receiving it for at least 6 months after treatment initiation, and those receiving a DMT before MS diagnosis, were not included in the study.

All included PwMS were followed through 2016 in nationwide registers[7, 8]. The following anonymised microdata, kept by four authorities, were used:

- <u>Stockholm Region:</u>
    - Swedish MS register (SMSreg)[7]: diagnosis age and date, type of MS, Expanded Disability Status Scale (EDSS), and type(s) and dates of DMT treatment(s)
- <u>National Board of Health and Welfare:</u>
    - National Patient Register (NPR)[8]: dates and diagnoses for inpatient and specialized outpatient healthcare (i.e., secondary healthcare)
    - Swedish Prescribed Drug Register (SPDR)[8]: dates, names, and costs for all prescribed drugs dispensed from pharmacies
    - Cause of Death Register[8]: year of death

For peer review only - http://bmjopen.bmj.com/site/about/guidelines.xhtml

- Statistics Sweden: Longitudinal Integration Database for Health Insurance and Labor Market Studies[8]; sex, birth year, educational level, country of birth, type of living area, family situation, annual income from work and/or benefits (yes/no)
- Swedish Social Insurance Agency: Micro Data for the Analysis of Social Insurance register[8]; dates, diagnoses, and grade (full- or part-time) of sickness absence (SA) in SA spells >14 days and disability pension (DP).

The registers were linked using the unique personal identity number that all residents in Sweden are assigned.

PwMS who switched from one DMT group to another relevant group before the 6-months cut-off, were allocated to the later DMT group. PwMS were censored at migration or death.

The following sociodemographic characteristics, measured at the year of diagnosis (index year), were considered: sex, age at index year, educational level, country of birth, type of living area, and family situation.

Four types of MS at diagnosis were identified, based on information available from SMSreg: Relapsing-Remitting MS (RRMS), Progressive-Relapsing MS (PRMS), Primary-Progressive MS (PPMS), and Secondary-Progressive MS (SPMS).

Multi-morbidity in the index year was derived utilizing the Rx-Risk Comorbidity Index[9], based on the type of drugs prescribed to PwMS according to SPDR.

Disability in MS was based on information from the Expanded Disability Status Scale (EDSS) available. The score of EDSS ranges from 0 to 10, with 0.5 step intervals (0 indicating no impairment, while 10 indicating death from MS)[10]. A clinically meaningful change in the EDSS score is a change of at least one point in patients with EDSS <5.5, and 0.5 point for those with EDSS of ≥5.5[11].

For peer review only - http://bmjopen.bmj.com/site/about/guidelines.xhtml

Where multiple EDSS scores for each calendar year were available, the highest EDSS value was retained. In case of missing EDSS scores in SMSreg, the mean annual score was used. This was computed from the scores of PwMS in the same DMT group, index year, and with the same type of MS. This method of imputation was chosen to account for individuals who had multiple missing EDSS values during the study follow-up.

2.1 Study outcomes

Healthcare resources consumed by PwMS as well as their SA and DP days were calculated and multiplied with unit costs (Table 1) to determine the societal COI of MS. The estimated COI included all such costs occurring for PwMS during a calendar year, irrespective of the main diagnosis behind the cost.

Inpatient and outpatient costs were calculated for each year of follow-up, by multiplying the number of days/visits, derived from NPR, with the cost per 1.0 retrospective nationwide weight from the diagnosis-related groups (DRG) system. Co-payment costs for these visits were also estimated by summing daily/per visit fees for inpatient and outpatient care. We assumed that the reimbursement periods started on 1 January, so costs exceeding the co-payment ceiling in Sweden for one year's period, were set to the maximum amount allowed per year (Table 1).

The cost of prescribed drugs was derived from the SPDR, based on the pharmacy's retail price of each dispensed drug package, including the co-payment paid by patients. The annual cost of drugs was calculated by summing the costs for all dispensed drugs during each year of follow-up.

The cost of DMTs not available through SPDR, i.e., MS drugs administered in the hospital setting and not dispensed by pharmacies (natalizumab and rituximab for the study period), was calculated using information in the SMSreg. The number of months on these treatments was multiplied with the cost per month of treatment (Table 1).

For peer review only - http://bmjopen.bmj.com/site/about/guidelines.xhtml

By adding costs of inpatient and outpatient care and co-payments for inpatient and outpatient care and drugs, the total annual healthcare costs per patient were calculated. Productivity losses were estimated based on the net days with SA and/or DP per year of follow-up, using the human capital approach[12]. Net months were multiplied with the age-adjusted mean salary, adding social security contributions made by employers (Table 1).

All people living in Sweden with income from work or unemployment benefits are from 16 years of age covered by the public SA insurance system and can be granted SA benefits if their work capacity is reduced due to disease or injury. The employer reimburses income loss during days 2-14 of a SA spell, after which SA benefits are administered by the Swedish Social Insurance Agency; for unemployed individuals, this happens from day 2. Therefore, to prevent bias in relation to employment status, only SA >14 days was included.

All people aged 19-65 years can be granted DP if their work capacity is long-term or permanently reduced[13]. Both SA and DP can be granted for full-time (100%) or part-time (75%, 50%, or 25%) of ordinary work hours[13], making it possible to have both partial SA and DP concurrently. Hence, we combined them to calculate the net days of SA and DP/year.

All costs were inflated to 2022 prices, applying the harmonized index for consumer prices[14]. Costs calculated in Swedish Krona (SEK) were then converted to Euros (EUR) in 2022 values (Table 1).

Table 1: Unit costs used in the calculation of healthcare costs and productivity losses

	Year	Value in 2022 SEK	Value in 2022 Euros[1]	Source
Average inpatient and outpatient cost per 1.0 DRG	2006	52,721 kr	4,960 €	Swedish Association of Local Authorities and Regions [Sveriges Kommuner och Regioner], KPP Somatik vård [15]
	2007	51,946 kr	4,887 €	
	2008	53,151 kr	5,000 €	
	2009	53,561 kr	5,039 €	
	2010	52,187 kr	4,910 €	
	2011	52,010 kr	4,893 €	

For peer review only - http://bmjopen.bmj.com/site/about/guidelines.xhtml

For peer review only

	2012	51,529 kr	4,848 €	
	2013	53,233 kr	5,008 €	
	2014	55,218 kr	5,195 €	
	2015	57,720 kr	5,430 €	
	2016	59,300 kr	5,579 €	
Co-payment for hospital stay (cost per day of stay)	2018	104 kr	10 €	Assume 100 SEK per day, as this is the case for the majority of the regions (including Stockholm).[16] The max co-payment amount for inpatient care was set to 1500 SEK per year (assumption for whole Sweden, based on information from the region Västra Götaland)[17]
Co-payment for visit in specialised care (cost per visit)	2018	284 kr	28 €	Swedish Association of Local Authorities and Regions (18). The max co-payment amount for outpatient care was set to 1100 SEK per year. Only one region in Sweden has a max co-payment less than 1100 SEK; so it was assumed 1100 SEK for the entire country. Swedish Association of Local Authorities and Regions[16]
Cost per month for natalizumab	2018	1,144 kr	111 €	Treatment with natalizumab is every 4th week (i.e. one per month)[18]; therefore, the cost per month of natalizumab was assumed to be the price for one dose of natalizumab (pharmacy's retail price)[18], excluding the cost of administration, which is captured in this study as an outpatient visit to the hospital
Cost per month for rituximab	2018	2,022 kr	197 €	Rituximab is used off-label in the treatment of MS; therefore, the exact treatment dosing was not available in the Swedish guidelines for MS treatment. A recent published study in Sweden regarding the use of rituximab for PwMS indicated that the drug dose is 500mg to 1000mg per treatment regime (here we assumed the mean, i.e 750mg per treatment regime), and the mean treatment interval for RRMS PwMS was 7.2 months per year[19]. The cost per month was then assumed to be 1.5 times the pharmacy's retail price per 500mg of rituximab injection, which was taken from FASS (9703.61 SEK)[20], divided with the frequency of treatment regimes in months (frequency: every 7.2 months) [19].
Monthly salary including employer contributions	2006	42,334	4,127 €	The average age-adjusted monthly wage (2018 values) for all employment types was retrieved[21]. It was multiplied with the annual employer contributions, available from the Swedish Tax Authority[22]. The final
	2007	42,457	4,139 €	
	2008	41,416	4,037 €	
	2009	46,112	4,495 €	

For peer review only - http://bmjopen.bmj.com/site/about/guidelines.xhtml

	2010	45,591	4,444 €	salary was then inflated to 2022 prices using annual Harmonised Indices of Consumer Prices [HICP] for healthcare available from Eurostat[14].
	2011	46,264	4,510 €	
	2012	46,283	4,512 €	
	2013	46,596	4,542 €	
	2014	47,701	4,650 €	
	2015	52,025	5,072 €	
	2016	49,797	4,854 €	

[1]The annual exchange rate for 2022 from SEK to Euros that was used was 10.6296. Source: Eurostat, Annual Exchange Rates Euro/ECU

For peer review only

2.2 Analyses

Sociodemographic characteristics, multi-morbidity, type of MS at diagnosis, MS disability progression, average COI per patient and year, including 95% confidence intervals (CI), were calculated for each of the three treatment groups.

Pearson's chi-squared test[23] and the likelihood ratio test[23] were used to explore whether the observed sociodemographic and multi-morbidity differences across the three DMT groups were statistically significant (p-value<0.05).

Two-tailed student's T-tests, with unequal variance[24] were used to assess the significance (p-value<0.05) of the disability and COI changes over time, within each treatment group, compared to baseline values (at diagnosis) and differences across the treatment groups.

A generalised linear model[25], with Poisson distribution for costs, was used to estimate mean cost per patient per year, and per group, and 95% CI of the estimation. The model was adjusted with the disability progression of PwMS over time, by setting the EDSS variable to the mean EDSS score across all the three groups for that year.

2.3 Patient and public involvement

None.

For peer review only - http://bmjopen.bmj.com/site/about/guidelines.xhtml

3 Results

In total, 3673 PwMS, receiving their first treatment with IFN (N=2696), GA (N=441), or NAT (N=536) between 2006-2015, were included. Sex, age distribution in the groups, educational level, family situation, country of birth, type of living area, and multi-morbidity (any) differed across the groups (p-value<0.05; Table 2). NAT had fewer people with any comorbidities (Table 2) vs. the IFN and GA groups. The baseline EDSS score was similar for the three groups (Supplementary Figure 1, in the Online Supplementary Material).

In Supplementary Table 1, in the Online Supplementary Material, the unadjusted mean per patient per year COI for MS is presented, over the 10-year follow-up, and for the three treatment groups. Figures 1a and b show the disability-adjusted healthcare costs and productivity losses. Disability-adjusted costs were also computed for all the cost components (Supplementary Figure 2a-f, in the Online Supplementary Material).

People on NAT had higher healthcare costs vs. the IFN and GA groups (p-value<0.05 for both comparisons), driven by the higher drug costs for this group (Supplementary Figure 2d, in the Online Supplementary Material). IFN and GA groups had similar healthcare costs and productivity losses (p-values>0.05). Productivity losses for NAT vs. GA were similar (p-value>0.05), while higher when compared to those on IFN (p-value>0.05), driven by the higher SA costs for the NAT group (p-value=0.055 vs the IFN group). NAT had the lowest DP costs vs. the other two groups at the start of follow-up, and there was a trend of lower DP costs compared to those for the GA group (p-value>0.05) over time.

For peer review only - http://bmjopen.bmj.com/site/about/guidelines.xhtml

Table 2: Sociodemographic and multi-morbidity characteristics at year of diagnosis (index year)

	Treatment with Interferons	Treatment with Glatiramer Acetate	Treatment with Natalizumab	Pearson's Chi-Square (p-value)	Log-likelihood test Chi-Square (p-value)
	N=2696	N=441	N=536		
	n (%)[a]	n(%)[a]	n(%)[a]		
**Sex**				7.49 (0.024)	7.70 (0.021)
Women	1882 (51.24)	333 (9.07)	364 (9.91)		
Men	814 (22.16)	108 (2.94)	172 (4.68)		
**Age at diagnosis (index year)**				107.26 (<.0001)	108.56 (<.0001)
20-25	321 (8.74)	45 (1.23)	114 (3.1)		
26-30	435 (11.84)	67 (1.82)	129 (3.51)		
31-35	465 (12.66)	65 (1.77)	114 (3.1)		
36-40	477 (12.99)	88 (2.4)	74 (2.01)		
41-45	463 (12.61)	75 (2.04)	46 (1.25)		
46-50	316 (8.6)	60 (1.63)	39 (1.06)		
51-55	219 (5.96)	41 (1.12)	20 (0.54)		
**Type of MS at diagnosis (index year)[b]**				2.24 (0.896)	2.25 (0.896)
RRMS	1960 (65.14)	302 (10.04)	419 (13.92)		
PPMS	23 (0.76)	4 (0.13)	4 (0.13)		
SPMS	187 (6.21)	31 (1.03)	33 (1.1)		
PRMS	31 (1.03)	6 (0.2)	9 (0.3)		
**Educational level[c]**				72.90 (<.0001)	58.09 (<.0001)
Elementary school	387 (10.84)	67 (1.88)	134 (3.75)		
High School	1290 (36.13)	210 (5.88)	225 (6.3)		
University/college	946 (26.5)	153 (4.29)	158 (4.43)		
**Country of birth[c]**				17.70 (0.0236)	14.70 (0.065)
Sweden	2348 (65.77)	390 (10.92)	454 (12.72)		
Nordic countries (except Sweden)	43 (1.2)	9 (0.25)	15 (0.42)		
EU27 (except Denmark, Finland, and Sweden)	42 (1.18)	8 (0.22)	16 (0.45)		
Rest of the world	190 (5.32)	23 (0.64)	32 (0.9)		
**Type of living area**				15.47 (0.004)	15.30 (0.004)
Big cities[c]	1020 (28.57)	142 (3.98)	210 (5.88)		

For peer review only - http://bmjopen.bmj.com/site/about/guidelines.xhtml

Medium sized cities	888 (24.87)	141 (3.95)	187 (5.24)		
Rural areas	715 (20.03)	147 (4.12)	120 (3.36)		
**Family situation**[c]				140.56 (<.0001)	123.74 (<.0001)
Married or cohabitant without children[d,e]	189 (5.29)	38 (1.06)	26 (0.73)		
Married or cohabitant with children[d,e]	1021 (28.6)	165 (4.62)	117 (3.28)		
Single without children[d,e]	1211 (33.92)	190 (5.32)	320 (8.96)		
Single with children[d,e]	174 (4.87)	28 (0.78)	25 (0.7)		
**Any multi-morbidity**				68.35 (<.0001)	53.92 (<.0001)
Yes	2580 (70.24)	424 (11.54)	466 (12.69)		
No	116 (3.16)	17 (0.46)	70 (1.91)		
**Anxiety/Depression**				4.10 (0.129)	3.91 (0.142)
Yes	137 (3.73)	28 (0.76)			
No	2559 (69.67)	413 (11.24)			

[a]The percentages are calculated as n divided with the total number of individuals in the study population (3673 if not otherwise indicated; see b)

[b]For 18% of the cohort, type of MS information was missing; so, information was available for 3009 PwMS in the study cohort

[c]The total number of individuals with this type of information in the study population (i.e., all individuals excluding those with missing information in this category) was 3570

[d]Only cohabitants with children in common are registered as cohabitants. Otherwise they were registered as single.

[e]Children were considered as being <18 years and living at home

For peer review only

For peer review only - http://bmjopen.bmj.com/site/about/guidelines.xhtml

4 Discussion

In this register-based prospective cohort study we explored the development of the COI of MS over time among newly diagnosed PwMS in Sweden. Costs were estimated in relation to what type of first line treatment was used; IFN, vs GA, vs NAT, with adjustment for MS disability progression. The analysis sub-groups were constructed based on the DMTs that were licensed to be used as 1st line therapies for MS in Sweden during 2006-2016, and for which we had information during the study's data period (2006-2016).

Overall, the general trends of decreasing healthcare costs and increasing productivity losses that were observed in this study are in line with previous COI studies in MS with a similar longitudinal design[26, 27]. No significant differences in healthcare costs were observed between the IFN and GA, while NAT had higher healthcare costs (p-value<0.05). This could be explained by the higher treatment cost with NAT[28], i.e., higher drug cost and healthcare utilization to administer and monitor PwMS on this DMT. As previous studies have shown, in the last two decades, the cost of drugs has become main COI driver in MS[4].

In addition, outpatient costs for PwMS on NAT could have played a role; they were higher for NAT (p-value>0.05). For the beginning of follow-up, until PwMS on NAT change therapy, this could be explained by that fact that these patients require at least one extra outpatient visit per month to get their treatment[29]. Before treatment, additional diagnostic tests (blood tests and in cases MRI scans) have to be performed, not needed for PwMS on other therapies, including IFN and GA[29]. Therefore, when compared to IFN and GA, healthcare costs for NAT could be amplified due to the extra outpatient visits needed for the treatment itself. Had no such visits and tests been necessary, outpatient costs NAT could have been at the same level as the ones for IFN and GA, or even lower.

For peer review only - http://bmjopen.bmj.com/site/about/guidelines.xhtml

Furthermore, DMTs were not randomly assigned to PwMS in our observational study, and therefore this study could not consider treatment decisions based on observed disease activity at the time of diagnosis. If treating neurologists assigned NAT to PwMS who could have had potentially higher disease activity, trying to prevent worse disease outcomes, this may have resulted in greater healthcare costs in the NAT group vs. IFN or GA, than those that could be expected if DMTs were randomly assigned to PwMS.

Towards the end of follow-up, when people may have switched to other DMTs, or stopped treatment, other factors could be associated with the observed healthcare cost differences, which have not been covered in this study, such as the progression of MS symptoms, relapses, and multi-morbidity.

Productivity losses for NAT and GA were similar; both had higher productivity losses vs. IFN (p-value<0.05). Since the comparative effectiveness of IFN and GA is similar[30], other unmeasured factors than disease progression, such as fatigue and multi-morbidity could explain this finding. On the other hand, even if treatment with NAT can lead to lower relapse rates[31], and hence possibly to slower disease progression, compared to IFN and GA, productivity losses for this group were higher. The need to take time off work when on NAT related to the administration of the therapy (outpatient visits to administer the therapy and run diagnostic tests), and recovery from the administration of this DMT, could explain part of this observed outcome[29], since it adds extra SA costs for NAT[32].

In fact, only SA costs were higher for NAT compared to the other groups; DP costs were lower than GA, indicating that even if PwMS receiving NAT as first-line treatment, are having higher MS disability at baseline, and probably higher disease activity compared to those receiving IFN or GA[29], still, their ability to maintain their long-term work capacity is higher.

For peer review only - http://bmjopen.bmj.com/site/about/guidelines.xhtml

Experiencing less relapses and in general less MS symptoms like fatigue, which can allow patients to maintain employment longer[33].

Previously published health economic models presenting the cost progression related to these three DMTs have often been criticised due to their limited data availability as well as the methodological complexities and uncertainties[33]. The COI of MS associated with new generation therapies, like NAT, compared to older therapies (IFN and GA) has not been studied so far involving longitudinal COI data with multiple years of follow-up for costs and disease progression.

4.1 Strengths and limitations

Main strengths of this study are its prospective cohort design, with several years of follow-up, and that data from high-quality administrative registers could be used. All PwMS in the SMSreg diagnosed with MS in the years 2006-2015 and receiving first-line therapy with NAT, IFN, or GA could be followed for several years in nationwide registers, eliminating drop outs and recall biases when using self-reported information from PwMS[4]. However, as there are no nationwide register data in Sweden regarding primary healthcare, rehabilitation, home help, home investments to improve mobility, and other out of pocket expenses this could not be included in our COI calculations. Hopefully future studies can include such information, to analyse how MS symptoms and multi-morbidity might be associated with the overall healthcare costs in MS, based on the DMT treatment administered.

In addition, the longest follow-up time we had was ten years after diagnosis; while this time can give important information regarding the COI progression for the three treatment groups, only very few patients had this many follow-up years. Therefore, the longer the follow-up, the higher the uncertainty is for the estimated COI presented. Moreover, while the impact of the first-line therapy on the COI can be observed for the time PwMS are receiving that treatment

For peer review only - http://bmjopen.bmj.com/site/about/guidelines.xhtml

and maybe for a short period of time afterwards, also other factors could be strongly associated with the COI progression over time after the first-line therapy was stopped, not measured in this study (e.g., type of second-line therapy, adherence to treatment, etc.).

Another important strength is that robust information on productivity losses was available enabling us to apply the societal perspective on the COI calculations. Productivity losses are the main long-term driver of the COI in MS as shown in this and previous studies[4]. However, short-term SA-spells of 14 days or less were not quantified possibly leading to underestimation of the productivity losses. Short-term SA spells have not been considered for both employed (including self-employed) and unemployed patients, hence no bias based on employment status has been introduced in the calculation of productivity losses.

Moreover, any productivity reductions while being present at work that could potentially be related to the presence of MS were not quantified. Furthermore, productivity losses incurred by partners of people with MS, i.e., informal care costs, were not included in the COI calculations; while measuring these costs was beyond the aim of this study, they are an important cost component when defining the overall COI of MS[4].

Similar to what previous COI in MS studies have done[4], we used the EDSS score to explore disability progression for PwMS in the three treatment groups. However, when EDSS information was missing, information had to be imputed. This has likely diluted the EDSS score used in the adjusted COI analysis. In addition, EDSS has been primarily developed and used for research purposes, not for clinical or treatment decisions in MS, and its scores are less effectively tracking disease progression over time[34]. Also, some MS symptoms, like fatigue, which are important and can limit the everyday activities of PwMS early in the disease trajectory, are not well detailed in the EDSS assessment[34], obtaining in this way a skewed result regarding the disability of PwMS. Additional clinical measures could have complemented our analysis.

For peer review only - http://bmjopen.bmj.com/site/about/guidelines.xhtml

Another limitation is that the generalizability of our findings can be limited. The study cohort was taken from SMSreg, which back in 2006 had approximately 50% coverage of all PwMS in Sweden, and 80% in 2015[35]. Assuming no distinct differences in the sociodemographic characteristics, multi-morbidity, and disability with the PwMS that our study did not include, our findings could be generalizable to all PwMS in Sweden. Still, in our cohort, an elevated number of people with SPMS at diagnosis was included vs. the expected distribution of the type of MS at diagnosis which could potentially hinder the generalizability of our findings. In addition, generalization of our findings to other countries is not possible, considering the differences in the organisation of healthcare and social security systems.

5 Conclusions

Using real-world data from Swedish nationwide registers, this study explored the association between the type of first-line DMT received (IFN vs. GA vs. NAT) with COI progression. The findings of a trend towards lower DP costs indicated that PwMS on NAT could potentially maintain their work capacity for a longer time. COI serves as an objective measure showing the importance of the use of second-generation treatments in maintaining at low levels the progression of MS over time, and hence the overall COI.

For peer review only - http://bmjopen.bmj.com/site/about/guidelines.xhtml

Data Availability Statement

The data used in this study is administered by the Division of Insurance Medicine, Karolinska Institutet, and cannot be made publicly available. According to the General Data Protection Regulation, the Swedish law SFS 2018:218, the Swedish Data Protection Act, the Swedish Ethical Review Act, and the Public Access to Information and Secrecy Act, these type of sensitive data can only be made available, after legal review, for researchers who meet the criteria for access to this type of sensitive and confidential data. Readers may contact Professor Kristina Alexanderson (kristina.alexanderson@ki.se) regarding the data.

Author Contributions

All authors (Korinna Karampampa, KK; Hanna Gyllensten, HG; Emilie Friberg, EF; Chantelle Murley, CM; Jan Hillert, JH; Andrius Kavaliunas, AK; Tomas Olsson, TO; Kristina Alexanderson, KA) contributed to the conceptualization of the research questions, the study design and methods. KK performed the analysis of the data, interpreted the study findings, and drafted the manuscript. All remaining authors (HG, EF, CM, JH, AK, TO, KA) assisted in the interpretation of the study findings and contributed with comments/suggestions and text to the manuscript.

Compliance with Ethical Standards

Patient consent for publication: Not required.

Ethics approval The project received ethical approval from the Regional Ethical Review Board in Stockholm. Approval numbers: 2007/762-31; 2009/23-32; 2009/1917-32; 2010/466-32; 2011/806-32; 2011/1710-32; and 2014/236-32.

Funding: This study was supported by an unrestricted investigator-initiated trial grant from Biogen to support the conduct of this study (Award number N/A). We utilised data from the

For peer review only - http://bmjopen.bmj.com/site/about/guidelines.xhtml

REWHARD consortium supported by the Swedish Research Council (grant number 2021-00154).

Conflicts of Interest: All authors (KK, HG, EF, CM, JH, AK, TO, KA) are employed or affiliated at the Department of Clinical Neuroscience, Karolinska Institutet, Stockholm, Sweden. KK is currently employed by Celgene/Bristol Myers Squibb; she initiated this study while being employed at Karolinska Institutet (employment ended in October 2019); since then, she has received no salary from Karolinska Institutet or other type of funding for this research. HG is currently employed part-time by Statfinn/EPID Research (which is part of IQVIA); both companies are contract research organizations that perform commissioned pharmacoepidemiological studies, and therefore are collaborating with several pharmaceutical companies. CM since submission of this paper has begun employment with Macanda AB. AK is currently also employed by Takeda Pharma AB. JH, KA, and EF are collaborating with several pharmaceutical companies; EF has received an unrestricted MS research grant from Celgene/Bristol Myers Squibb .TO has received advisory board and/or lecture honoraria, and unrestricted MS research grants from Biogen, Novartis, Sanofi, Merck and Roche.

For peer review only - http://bmjopen.bmj.com/site/about/guidelines.xhtml

References

1. Dutta R and Trapp BD. Mechanisms of neuronal dysfunction and degeneration in multiple sclerosis. *Prog Neurobiol*. 2011; 93: 1-12.
2. Ahlgren C, Oden A and Lycke J. High nationwide incidence of multiple sclerosis in Sweden. *PLoS One*. 2014; 9: e108599.
3. Gyllensten H, Wiberg M, Alexanderson K, et al. Costs of illness of multiple sclerosis in Sweden: a population-based register study of people of working age. *Eur J Health Econ*. 2017.
4. Ernstsson O, Gyllensten H, Alexanderson K, Tinghög P, Friberg E and Norlund A. Cost of Illness of Multiple Sclerosis - A Systematic Review. *PLoS One*. 2016; 11: e0159129.
5. Cerqueira JJ, Compston DAS, Geraldes R, et al. Time matters in multiple sclerosis: can early treatment and long-term follow-up ensure everyone benefits from the latest advances in multiple sclerosis? *J Neurol Neurosurg Psychiatry*. 2018; 89: 844-50.
6. Iannazzo S, Iliza AC and Perrault L. Disease-Modifying Therapies for Multiple Sclerosis: A Systematic Literature Review of Cost-Effectiveness Studies. *Pharmacoeconomics*. 2018; 36: 189-204.
7. Hillert J and Stawiarz L. The Swedish MS registry - clinical support tool and scientific resource. *Acta neurologica Scandinavica*. 2015; 132: 11-9.
8. Ludvigsson JF, Almqvist C, Bonamy AK, et al. Registers of the Swedish total population and their use in medical research. *Eur J Epidemiol*. 2016; 31: 125-36.
9. Pratt NL, Kerr M, Barratt JD, et al. The validity of the Rx-Risk Comorbidity Index using medicines mapped to the Anatomical Therapeutic Chemical (ATC) Classification System. *BMJ Open*. 2018; 8: e021122.
10. Collins CD, Ivry B, Bowen JD, et al. A comparative analysis of Patient-Reported Expanded Disability Status Scale tools. *Mult Scler*. 2016; 22: 1349-58.
11. Goodkin DE. EDSS reliability. *Neurology*. 1991; 41: 332.
12. Drummond MF, Sculpher MJ, Claxton K, Stoddart GL and Torrance GW. *Methods for the economic evaluation of health care programmes*. Oxford university press, 2015.
13. National Insurance Agency [Försäkringskassan]. Social Insurance in Figures 2017. 2017.
14. Eurostat. HICP - all items - annual average indices (2015=100). https://ec.europa.eu/eurostat/tgm/table.do?tab=table&init=1&language=en&pcode=tec00027&plugin=12019.
15. Swedish Association of Local Authorities and Regions [Sveriges Kommuner och Landsting]. Economic statistics - KPP Somatik - SKL. https://skl.se/ekonomijuridikstatistik/statistik/kostnadperpatientkpp/kppsomatik.1077.html2019.
16. Swedish Association of Local Authorities and Regions [Sveriges Kommuner och Landsting]. Economic statistics - Patientavgifter fr.o.m. den 1 januari 2018. https://skl.se/download/18.3ee4cc4f1611af77b89179dd/1517495154691/avgift_%C3%B6ppenv%C3%A5rd_inkl.missiv_slutenavg18_19jan18.pdf2018.
17. Vårdguiden 1177. Patient fees in Västra Götaland county in Sweden [Patientavgifter i Västra Götaland]. https://www.1177.se/Vastra-Gotaland/Regler-och-rattigheter/Patientavgifter-i-Vastra-Gotaland/2018.
18. FASS.se. Tysabri. https://www.fass.se/LIF/product?nplId=20040916001115&userType=0&docType=6&scrollPosition=751.33331298828122019.
19. Salzer J, Svenningsson R, Alping P, et al. Rituximab in multiple sclerosis: A retrospective observational study on safety and efficacy. *Neurology*. 2016; 87: 2074-81.

For peer review only - http://bmjopen.bmj.com/site/about/guidelines.xhtml

20. FASS.se. Rituximab - price. https://www.fass.se/LIF/product?userType=2&nplId=19980602000029&docType=30&scrollPosition=600s2019.
21. Statistics Sweden. Genomsnittlig månadslön 1973–2018. https://www.scb.se/hitta-statistik/statistik-efter-amne/arbetsmarknad/loner-och-arbetskostnader/lonestrukturstatistik-privat-sektor-slp/pong/tabell-och-diagram/genomsnittlig-manadslon-1973-/2019.
22. Swedish Tax Authority [Skatteverket]. Belopp och procent för åren 2019-2004 - privat. https://www.skatteverket.se/privat/skatter/beloppochprocent.4.18e1b10334ebe8bc80004109.html 2019.
23. Moore DS. Tests of chi-squared type. *Goodness-of-fit-techniques*. Routledge, 2017, p. 63-96.
24. Kim TK. T test as a parametric statistic. *Korean journal of anesthesiology*. 2015; 68: 540-6.
25. McCullagh P and Nelder J. Generalized linear models., 2nd edn.(Chapman and Hall: London). *Standard book on generalized linear models*. 1989.
26. Gyllensten H, Kavaliunas A, Murley C, et al. Costs of illness progression for different multiple sclerosis phenotypes: A population-based study in Sweden. *Submitted*. 2018.
27. Karampampa K, Gyllensten H, Yang F, et al. Healthcare, Sickness Absence, and Disability Pension Cost Trajectories in the First 5 Years After Diagnosis with Multiple Sclerosis: A Prospective Register-Based Cohort Study in Sweden. *Pharmacoecon Open*. 2019.
28. Hartung DM, Bourdette DN, Ahmed SM and Whitham RH. The cost of multiple sclerosis drugs in the US and the pharmaceutical industry: Too big to fail? *Neurology*. 2015; 84: 2185-92.
29. Piehl F, Olsson T, Lycke J, et al. Läkemedelsbehandling av multipel skleros (MS)– bakgrundsdokumentation. https://lakemedelsverket.se/upload/halso-och-sjukvard/behandlingsrekommendationer/bakg_dok/Lakemedelsbehandling_av_multipel_skleros_MS_bakgrundsdokumentation_webb.pdf.
30. Melendez-Torres GJ, Armoiry X, Court R, et al. Comparative effectiveness of beta-interferons and glatiramer acetate for relapsing-remitting multiple sclerosis: systematic review and network meta-analysis of trials including recommended dosages. *BMC Neurol*. 2018; 18: 162.
31. Spelman T, Kalincik T, Jokubaitis V, et al. Comparative efficacy of first-line natalizumab vs IFN-beta or glatiramer acetate in relapsing MS. *Neurol Clin Pract*. 2016; 6: 102-15.
32. Kwiatkowski A, Marissal JP, Pouyfaucon M, Vermersch P, Hautecoeur P and Dervaux B. Social participation in patients with multiple sclerosis: correlations between disability and economic burden. *BMC Neurol*. 2014; 14: 115.
33. Glanz BI, Degano IR, Rintell DJ, Chitnis T, Weiner HL and Healy BC. Work productivity in relapsing multiple sclerosis: associations with disability, depression, fatigue, anxiety, cognition, and health-related quality of life. *Value Health*. 2012; 15: 1029-35.
34. Meyer-Moock S, Feng YS, Maeurer M, Dippel FW and Kohlmann T. Systematic literature review and validity evaluation of the Expanded Disability Status Scale (EDSS) and the Multiple Sclerosis Functional Composite (MSFC) in patients with multiple sclerosis. *BMC Neurol*. 2014; 14: 58.
35. Swedish Neuroregistries MS. Visualisation and Analysis platform (VAP) for Multiple Sclerosis - Coverage ratio broken down by county, gender and the last follow-up date [Täckningsgrad uppdelat för län, kön och sista uppföljningsdatum], Coverage ratio against the patient register, by year [Täckningsgrad mot patientregistret, år].

For peer review only - http://bmjopen.bmj.com/site/about/guidelines.xhtml

https://www.neuroreg.se/en.html/multiple-sclerosis-realtime-data-and-results-vap: Swedish Neuroregistries - Multiple Sclerosis, 2019.

For peer review only

For peer review only - http://bmjopen.bmj.com/site/about/guidelines.xhtml

Figure 1(A,B)

Healthcare costs (A) and Productivity losses (B) (estimated mean from the regression) from baseline (year at MS diagnosis) to the end of follow-up, by treatment groups, adjusted for disability progression during the follow-up

For peer review only

For peer review only - http://bmjopen.bmj.com/site/about/guidelines.xhtml

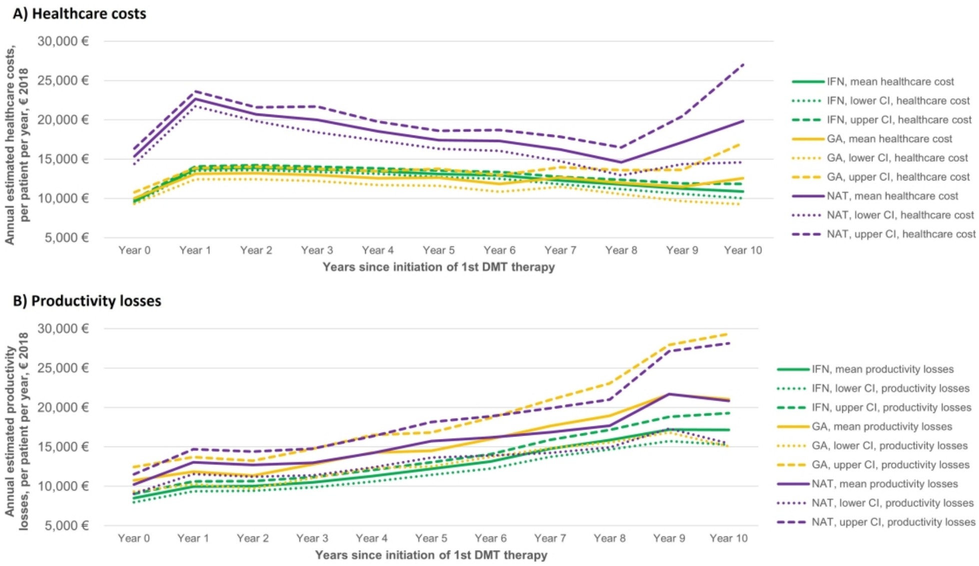

Healthcare costs (A) and Productivity losses (B) (estimated mean from the regression) from baseline (year at MS diagnosis) to the end of follow-up, by treatment groups, adjusted for disability progression during the follow-up

294x170mm (300 x 300 DPI)

Online Supplementary Material

Supplementary Table 1: Mean costs per patient per year, mean costs [95% confidence intervals] in € 2022 prices (crude observed means and confidence intervals, unadjusted), by treatment groups

	Year 0 (year of MS diagnosis)	Year 1	Year 2	Year 3	Year 4	Year 5	Year 6	Year 7	Year 8	Year 9	Year 10
**Interferon group**	**N=2696**	**N=2696**	**N=2644**	**N=2495**	**N=2219**	**N=1879**	**N=1556**	**N=1260**	**N=957**	**N=627**	**N=320**
Inpatient costs	1774 [1631-1918]	1079 [923-1236]	1327 [1147-1508]	1236 [1068-1405]	1148 [975-1321]	1241 [1022-1460]	1281 [1053-1509]	1082 [872-1291]	1439 [1004-1874]	1006 [707-1304]	1042 [608-1477]
Outpatient costs	1800 [1747-1853]	1368 [1313-1424]	1387 [1329-1446]	1417 [1352-1482]	1444 [1378-1510]	1528 [1453-1604]	1537 [1454-1619]	1626 [1510-1742]	1635 [1532-1738]	1726 [1594-1858]	1754 [1572-1935]
Co-payments	107 [106-109]	74 [72-75]	69 [67-71]	66 [64-68]	66 [64-68]	67 [65-69]	67 [64-69]	66 [64-69]	67 [64-70]	65 [61-69]	69 [63-74]
Drug costs	5904 [5733-6074]	11170 [10984-11357]	11029 [10811-11247]	10843 [10596-11090]	10686 [10412-10961]	10113 [9805-10421]	9850 [9504-10196]	9361 [8974-9748]	8624 [8176-9072]	8394 [7835-8954]	7927 [7120-8735]
**Total healthcare costs**	9585 [9345-9826]	13701 [13439-13962]	13813 [13512-14114]	13562 [13243-13882]	13345 [13001-13688]	12948 [12551-13346]	12734 [12303-13165]	12135 [11672-12598]	11765 [11145-12385]	11191 [10540-11842]	10792 [9892-11693]
SA costs	6927 [6469-7386]	7816 [7263-8369]	6586 [6078-7094]	5320 [4849-5790]	4649 [4167-5131]	4446 [3955-4937]	4113 [3595-4630]	3966 [3403-4529]	4410 [3688-5131]	3988 [3199-4777]	3896 [2837-4955]
DP costs	1890 [1555-2225]	2547 [2168-2926]	3887 [3433-4341]	5614 [5069-6159]	7386 [6730-8042]	8590 [7832-9348]	10068 [9172-10965]	11944 [10865-13023]	12781 [11501-14062]	14792 [13118-16466]	15204 [12888-17519]
**Total productivity losses**	8817 [8273-9362]	10356 [9709-11004]	10473 [9818-11128]	10934 [10244-11623]	12035 [11259-12811]	13036 [12175-13897]	14181 [13184-15177]	15910 [14747-17073]	17191 [15804-18578]	18780 [17023-20536]	19100 [16687-21512]
**Glatiramer Acetate group**	**N=441**	**N=441**	**N=430**	**N=406**	**N=348**	**N=292**	**N=225**	**N=170**	**N=116**	**N=66**	**N=31**
Inpatient costs	2217 [1733-2702]	1323 [862-1784]	1183 [775-1590]	1136 [731-1540]	1429 [956-1902]	1538 [911-2165]	880 [445-1315]	1401 [827-1974]	1324 [602-2047]	550 [134-967]	1417 [-911-3744]
Outpatient costs	2042 [1894-2189]	1506 [1361-1652]	1510 [1369-1651]	1566 [1407-1726]	1627 [1428-1826]	1716 [1505-1927]	1709 [1478-1940]	1734 [1475-1993]	1995 [1555-2435]	2165 [1573-2758]	2739 [1162-4316]
Co-payments	109 [104-113]	79 [74-84]	73 [69-78]	72 [67-76]	71 [65-77]	75 [69-80]	70 [64-76]	77 [69-86]	74 [64-84]	71 [60-82]	73 [57-90]
Drug costs	5670 [5227-6112]	10292 [9803-10780]	10257 [9708-10805]	9891 [9305-10476]	9211 [8553-9868]	9026 [8257-9796]	8846 [7966-9726]	9281 [8243-10320]	8390 [7164-9616]	8467 [6740-10195]	8282 [5644-10921]
**Total healthcare costs**	10037 [9341-10733]	13235 [12498-13972]	13023 [12252-13794]	12664 [11915-13414]	12338 [11483-13192]	12355 [11316-13393]	11505 [10464-12546]	12493 [11267-13719]	11783 [10289-13277]	11254 [9312-13196]	12511 [8603-16420]

For peer review only

SA costs	8118 [6848-9389]	8681 [7229-10132]	7190 [5834-8546]	6649 [5300-7998]	6105 [4718-7492]	5155 [3768-6541]	5154 [3627-6680]	5067 [3422-6712]	5191 [2897-7485]	4605 [2407-6803]	3815 [656-6975]
DP costs	3329 [2218-4441]	4127 [2896-5359]	5312 [3915-6709]	7615 [5945-9285]	9754 [7778-11730]	11788 [9474-14103]	13142 [10471-15812]	15090 [11772-18407]	18078 [13827-22329]	18791 [13241-24341]	19044 [11703-26384]
**Total productivity losses**	11447 [9871-13024]	12882 [11104-14661]	12502 [10690-14314]	14264 [12271-16257]	15859 [13620-18097]	16943 [14442-19444]	18295 [15397-21194]	20157 [16747-23566]	23269 [18820-27719]	23396 [17673-29118]	22859 [15118-30600]
**Natalizumab group**	**N=536**	**N=536**	**N=453**	**N=379**	**N=315**	**N=260**	**N=197**	**N=153**	**N=118**	**N=65**	**N=26**
Inpatient costs	4862 [4202-5521]	3230 [2519-3942]	2317 [1753-2881]	3043 [1683-4403]	2290 [1549-3031]	1725 [1074-2376]	1642 [920-2363]	1836 [1045-2628]	1087 [475-1699]	2951 [1095-4806]	3302 [166-6438]
Outpatient costs	2662 [2475-2848]	2580 [2372-2788]	2252 [2037-2467]	2161 [1918-2404]	2492 [2188-2795]	2254 [2007-2501]	2202 [1904-2501]	2397 [2006-2788]	2227 [1853-2601]	2831 [2238-3425]	3027 [1930-4124]
Co-payments	135 [130-140]	102 [97-107]	87 [83-91]	87 [82-92]	88 [82-94]	90 [83-96]	82 [75-89]	86 [78-94]	78 [70-86]	92 [77-107]	94 [71-117]
Drug costs	7568 [7064-8072]	16023 [15485-16560]	15930 [15291-16569]	14550 [13770-15330]	13512 [12619-14405]	13295 [12277-14312]	13368 [12214-14522]	11826 [10469-13184]	11336 [9816-12856]	11197 [8998-13396]	13386 [8164-18608]
**Total healthcare costs**	15226 [14283-16170]	22588 [21691-23484]	20586 [19724-21447]	19840 [18220-21461]	18382 [17204-19560]	17364 [16240-18489]	17293 [16013-18573]	16147 [14604-17689]	14728 [12909-16546]	17071 [14005-20137]	19809 [13284-26335]
SA costs	9683 [8492-10875]	11855 [10312-13399]	9867 [8358-11376]	7751 [6306-9197]	7484 [5784-9184]	7023 [5309-8737]	5292 [3639-6946]	5402 [3574-7229]	4788 [2807-6770]	5343 [2248-8438]	3317 [287-6348]
DP costs	2327 [1498-3157]	3392 [2388-4396]	5034 [3727-6341]	7629 [5942-9317]	9239 [7241-11237]	12062 [9629-14495]	14303 [11429-17177]	15873 [12321-19425]	17963 [13694-22233]	19921 [13856-25986]	20632 [10822-30443]
**Total productivity losses**	12010 [10646-13375]	15241 [13486-16996]	14901 [13047-16755]	15381 [13343-17418]	16723 [14354-19091]	19085 [16427-21743]	19596 [16559-22632]	21275 [17620-24930]	22752 [18472-27032]	25264 [19184-31343]	23950 [14408-33491]

For peer review only

Supplementary Figure 1: EDSS score progression from baseline (index year) to the end of follow-up, by IFN vs GA vs NAT treatment groups

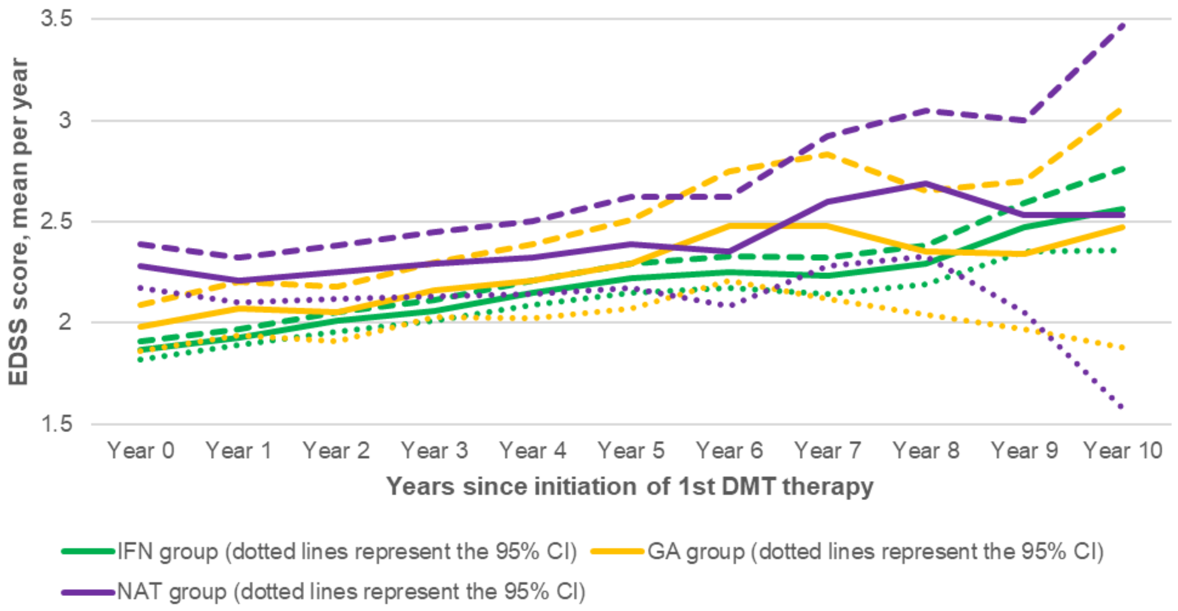

For peer review only

Supplementary Figure 2 (a-f): Progression (estimated mean from the regression) for all cost components, from baseline (year at MS diagnosis) to the end of follow-up, by treatment groups, adjusted for disability progression during follow-up

A) Inpatient costs

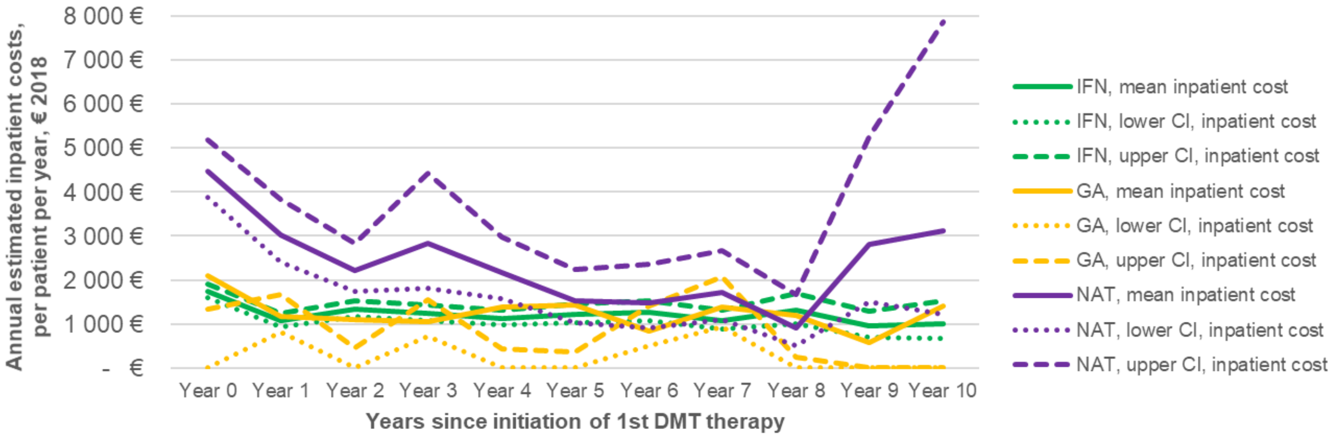

B) Outpatient costs

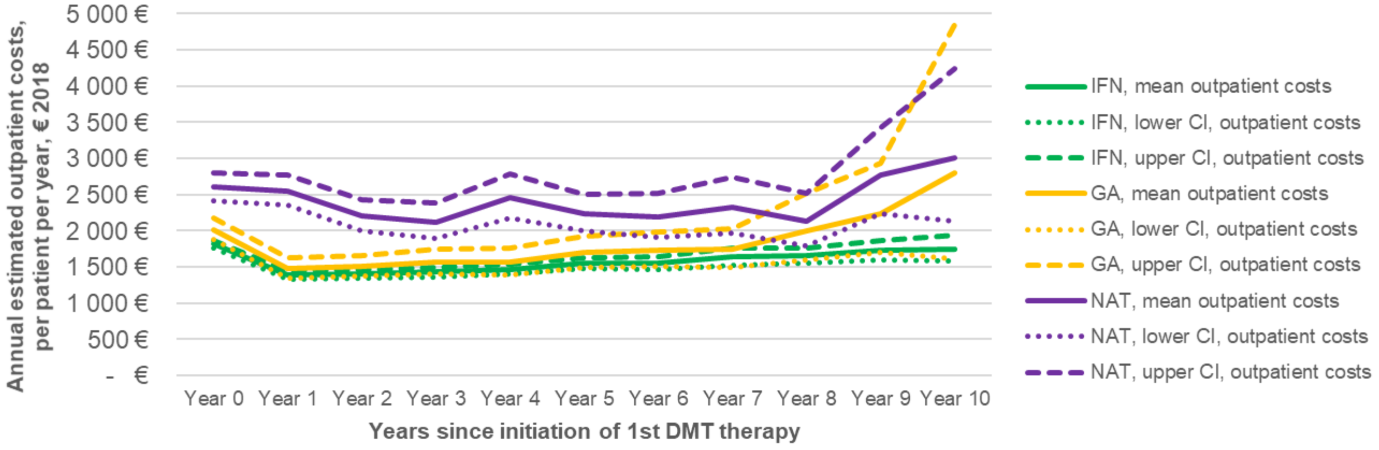

C) Co-payments

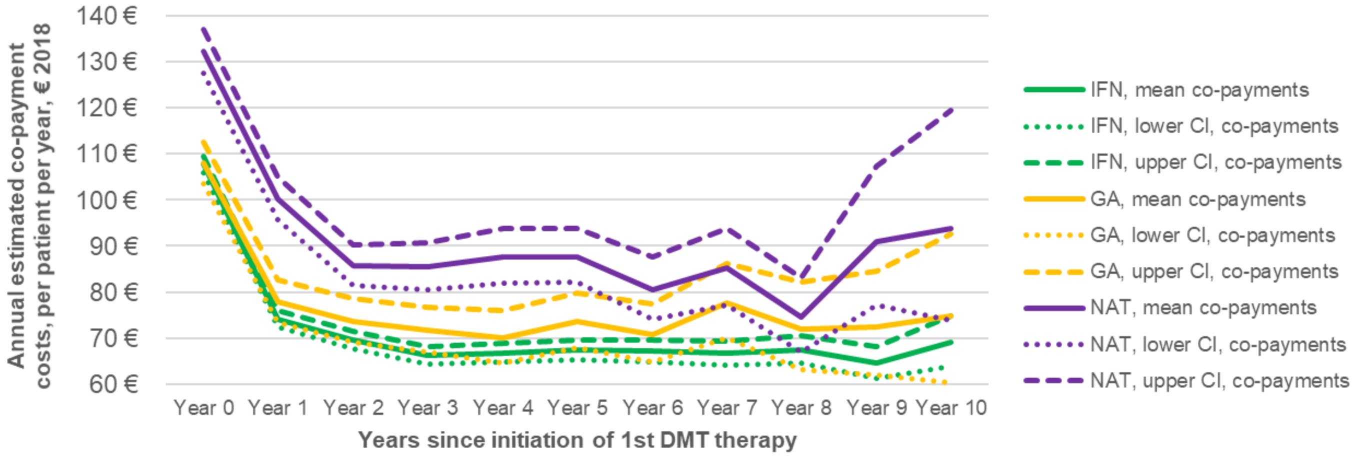

D) Drug costs

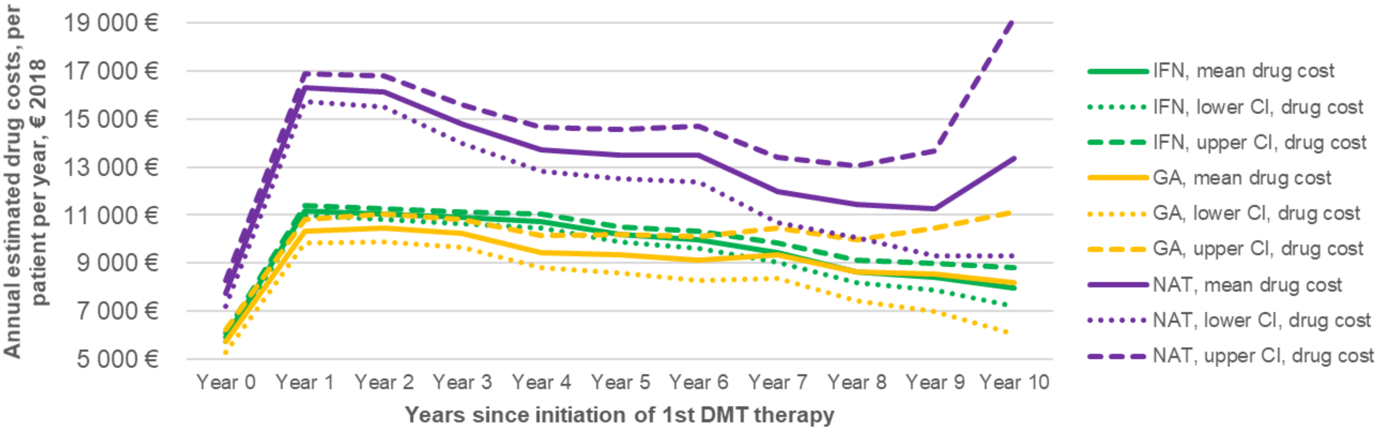

E) Sickness absence costs

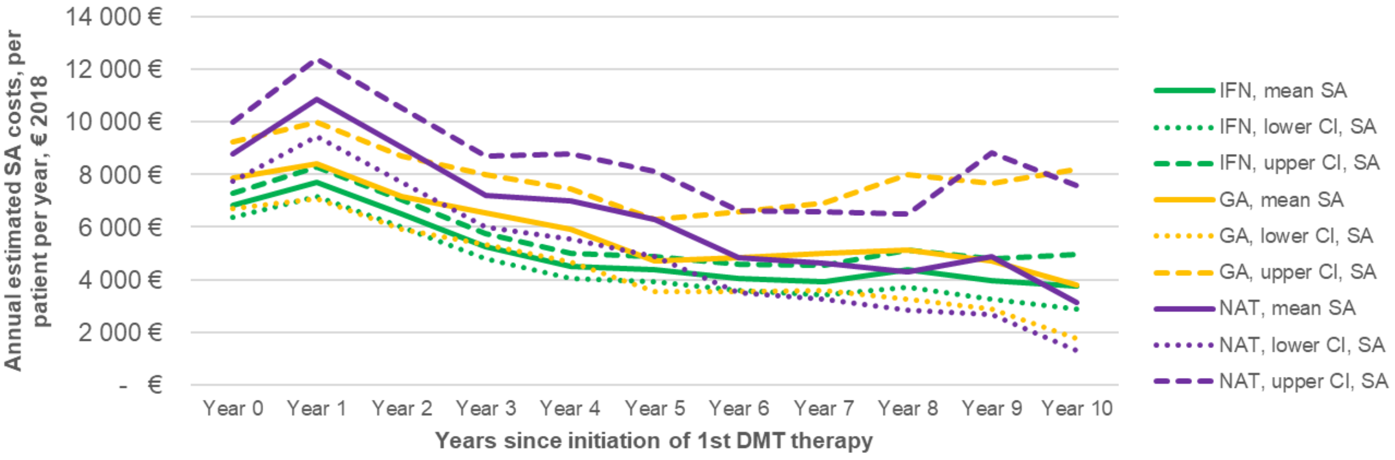

F) Disability pension costs

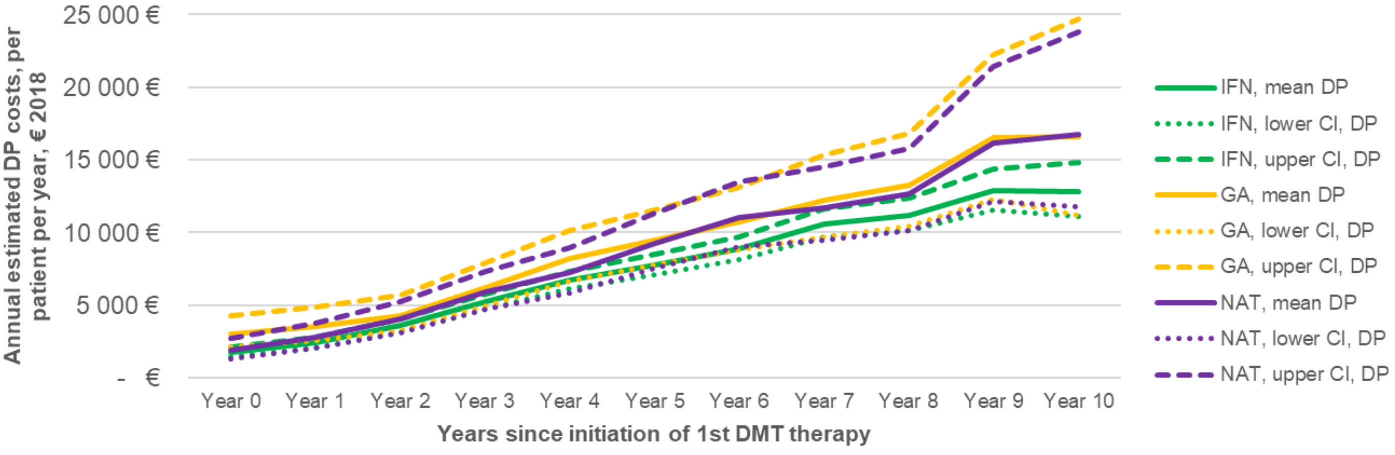

STROBE Statement—Checklist of items that should be included in reports of ***cohort studies***

	Item No	Recommendation	Page No
**Title and abstract**	1	(*a*) Indicate the study's design with a commonly used term in the title or the abstract	1
		(*b*) Provide in the abstract an informative and balanced summary of what was done and what was found	2
**Introduction**			
Background/rationale	2	Explain the scientific background and rationale for the investigation being reported	4
Objectives	3	State specific objectives, including any prespecified hypotheses	4
**Methods**			
Study design	4	Present key elements of study design early in the paper	5
Setting	5	Describe the setting, locations, and relevant dates, including periods of recruitment, exposure, follow-up, and data collection	5-6
Participants	6	(*a*) Give the eligibility criteria, and the sources and methods of selection of participants. Describe methods of follow-up	5-6
		(*b*) For matched studies, give matching criteria and number of exposed and unexposed	n.a.
Variables	7	Clearly define all outcomes, exposures, predictors, potential confounders, and effect modifiers. Give diagnostic criteria, if applicable	5-9
Data sources/ measurement	8*	For each variable of interest, give sources of data and details of methods of assessment (measurement). Describe comparability of assessment methods if there is more than one group	5-7
Bias	9	Describe any efforts to address potential sources of bias	5-7
Study size	10	Explain how the study size was arrived at	5
Quantitative variables	11	Explain how quantitative variables were handled in the analyses. If applicable, describe which groupings were chosen and why	8-9
Statistical methods	12	(*a*) Describe all statistical methods, including those used to control for confounding	8-9
		(*b*) Describe any methods used to examine subgroups and interactions	8-9
		(*c*) Explain how missing data were addressed	7-9
		(*d*) If applicable, explain how loss to follow-up was addressed	6-9
		(*e*) Describe any sensitivity analyses	n.a.
**Results**			
Participants	13*	(a) Report numbers of individuals at each stage of study—eg numbers potentially eligible, examined for eligibility, confirmed eligible, included in the study, completing follow-up, and analysed	10
		(b) Give reasons for non-participation at each stage	5-10
		(c) Consider use of a flow diagram	n.a.
Descriptive data	14*	(a) Give characteristics of study participants (eg demographic, clinical, social) and information on exposures and potential confounders	10
		(b) Indicate number of participants with missing data for each variable of interest	10
		(c) Summarise follow-up time (eg, average and total amount)	10
Outcome data	15*	Report numbers of outcome events or summary measures over time	10

For peer review only - http://bmjopen.bmj.com/site/about/guidelines.xhtml

Main results	16	(*a*) Give unadjusted estimates and, if applicable, confounder-adjusted estimates and their precision (eg, 95% confidence interval). Make clear which confounders were adjusted for and why they were included	10, 21-26
		(*b*) Report category boundaries when continuous variables were categorized	n.a.
		(*c*) If relevant, consider translating estimates of relative risk into absolute risk for a meaningful time period	n.a.
Other analyses	17	Report other analyses done—eg analyses of subgroups and interactions, and sensitivity analyses	10, 21-26
**Discussion**			
Key results	18	Summarise key results with reference to study objectives	11-15
Limitations	19	Discuss limitations of the study, taking into account sources of potential bias or imprecision. Discuss both direction and magnitude of any potential bias	13-15
Interpretation	20	Give a cautious overall interpretation of results considering objectives, limitations, multiplicity of analyses, results from similar studies, and other relevant evidence	11-15
Generalisability	21	Discuss the generalisability (external validity) of the study results	14-15
**Other information**			
Funding	22	Give the source of funding and the role of the funders for the present study and, if applicable, for the original study on which the present article is based	16-17

*Give information separately for exposed and unexposed groups.

Note: An Explanation and Elaboration article discusses each checklist item and gives methodological background and published examples of transparent reporting. The STROBE checklist is best used in conjunction with this article (freely available on the Web sites of PLoS Medicine at http://www.plosmedicine.org/, Annals of Internal Medicine at http://www.annals.org/, and Epidemiology at http://www.epidem.com/). Information on the STROBE Initiative is available at http://www.strobe-statement.org.

For peer review only

For peer review only - http://bmjopen.bmj.com/site/about/guidelines.xhtml

BMJ Open

Disease-modifying therapies and cost-of-illness progression among people newly diagnosed with multiple sclerosis: a national register-based cohort study covering treatment initiation with interferons, glatiramer acetate, or natalizumab

Journal:	*BMJ Open*
Manuscript ID	bmjopen-2022-067516.R2
Article Type:	Original research
Date Submitted by the Author:	24-Apr-2023

Complete List of Authors:	Karampampa, Korinna; Karolinska Institutet, Department of Clinical Neuroscience<br>Gyllensten, Hanna; University of Gothenburg, Institute of Health and Care Sciences; University of Gothenburg Centre for Person-Centred Care,<br>Friberg, Emilie; Karolinska Institute<br>Murley, Chantelle; Karolinska Institutet Department of Clinical Neuroscience, Clinical Neuroscience<br>Kavaliunas, A; Karolinska Universitetssjukhuset<br>Hillert, Jan; Karolinska Institute, Clinical Neuroscience<br>Olsson, Tomas; Karolinska Institute<br>Alexanderson, Kristina; karolinska institutet, Department of Clinical Neuroscience

**Primary Subject Heading**:	Health economics
Secondary Subject Heading:	Neurology, Epidemiology
Keywords:	Multiple sclerosis < NEUROLOGY, HEALTH ECONOMICS, Health economics < HEALTH SERVICES ADMINISTRATION & MANAGEMENT

SCHOLARONE™
Manuscripts

I, the Submitting Author has the right to grant and does grant on behalf of all authors of the Work (as defined in the below author licence), an exclusive licence and/or a non-exclusive licence for contributions from authors who are: i) UK Crown employees; ii) where BMJ has agreed a CC-BY licence shall apply, and/or iii) in accordance with the terms applicable for US Federal Government officers or employees acting as part of their official duties; on a worldwide, perpetual, irrevocable, royalty-free basis to BMJ Publishing Group Ltd ("BMJ") its licensees and where the relevant Journal is co-owned by BMJ to the co-owners of the Journal, to publish the Work in this journal and any other BMJ products and to exploit all rights, as set out in our licence.

The Submitting Author accepts and understands that any supply made under these terms is made by BMJ to the Submitting Author unless you are acting as an employee on behalf of your employer or a postgraduate student of an affiliated institution which is paying any applicable article publishing charge ("APC") for Open Access articles. Where the Submitting Author wishes to make the Work available on an Open Access basis (and intends to pay the relevant APC), the terms of reuse of such Open Access shall be governed by a Creative Commons licence – details of these licences and which Creative Commons licence will apply to this Work are set out in our licence referred to above.

Other than as permitted in any relevant BMJ Author's Self Archiving Policies, I confirm this Work has not been accepted for publication elsewhere, is not being considered for publication elsewhere and does not duplicate material already published. I confirm all authors consent to publication of this Work and authorise the granting of this licence.

For peer review only

Disease-modifying therapies and cost-of-illness progression among people newly diagnosed with multiple sclerosis: a national register-based cohort study covering treatment initiation with interferons, glatiramer acetate, or natalizumab

Korinna Karampampa[1], Hanna Gyllensten[2], Emilie Friberg[1], Chantelle Murley[1], , Andrius Kavaliunas[1], Jan Hillert[1],Tomas Olsson[1], Kristina Alexanderson[1]

Affiliations

[1]Department of Clinical Neuroscience, Karolinska Institutet, SE-171 77 Stockholm, Sweden

[2]Institute of Health and Care Science, University of Gothenburg, Gothenburg, Sweden

Correspondence to:

Korinna Karampampa, MSc, PhD

Division of Insurance Medicine, Department of Clinical Neuroscience, Karolinska Institutet, SE-171 77 Stockholm, Sweden

E-mail: korinna.karampampa@ki.se

Phone: +46 704 33 74 15

For peer review only - http://bmjopen.bmj.com/site/about/guidelines.xhtml

ABSTRACT

Objectives: Disease modifying therapies (DMTs) can slow disease progression in multiple sclerosis (MS). The objective of this study was to explore the cost-of-illness (COI) progression among newly diagnosed people with MS in relation to the first DMT received.

Design and setting: A cohort study utilizing data from nationwide registers in Sweden.

Participants: People with MS (PwMS) in Sweden first diagnosed in 2006-2015, when aged 20-55, receiving first-line therapy with interferons (IFN), glatiramer acetate (GA), or natalizumab (NAT). They were followed up through 2016.

Outcome measures: Outcomes (in Euros, €) were: 1) secondary healthcare costs: specialized outpatient and inpatient care including out-of-pocket expenditure, DMTs including hospital administered MS therapies, and prescribed drugs, and 2) productivity losses: sickness absence and disability pension. Descriptive statistics and Poisson regression were computed, adjusting for disability progression using the Expanded Disability Status Scale.

Results: 3673 newly diagnosed PwMS who were treated with IFN (N=2696), GA (N=441), or NAT (N=536) were identified. Healthcare costs were similar for the INF and GA groups, while the NAT group had higher costs (p-value<0.05), owing to DMT and outpatient costs. IFN had lower productivity losses than NAT and GA (p-value>0.05), driven by fewer sickness absence days. NAT had a trend towards lower disability pension costs compared to GA (p-value>0.05).

Conclusions: Similar trends over time for healthcare costs and productivity losses were identified across the DMT sub-groups. PwMS on NAT maintained their work capacity for a longer time compared to those on GA, potentially leading to lower disability pension costs over time. COI serves as an objective measure to explore the importance of DMTs in maintaining low levels of progression of MS over time.

For peer review only - http://bmjopen.bmj.com/site/about/guidelines.xhtml

Strengths and limitations of this study

- Data from nationwide registers with nationwide coverage were used, preventing recall and selection bias in the productivity loss and healthcare cost calculations.
- Additional costs (primary healthcare, rehabilitation, home help, home investments to improve mobility, other out-of-pocket expenses, sickness absence spells of 14 days or less, productivity reductions while being present at work, and informal care costs) were unable to be included in our cost-of-illness (COI) calculations due to lack of register information.
- Some factors that could potentially be strongly associated with the COI progression over time after the first-line therapy was stopped were not measured in this study (eg, type of second-line therapy, adherence to treatment, etc).
- While Expanded Disability Status Scale (EDSS) scores were used to adjust the COI progression for imbalances in disability progression across the three treatment groups as per established methodology, EDSS does not capture all relevant MS symptoms that can limit everyday activities, such as fatigue.
- The observational study design prevents any strong conclusions with respect to causality, and limitations of the dataset and setting could limit the generalizability of our findings.

For peer review only - http://bmjopen.bmj.com/site/about/guidelines.xhtml

Introduction

Multiple sclerosis (MS) is a highly disabling chronic neurological disease[1], usually diagnosed when aged 20-40 years[1]. Sweden has one of the highest prevalence of MS in Europe, estimated at 189 cases over 100,000 individuals[2].

In 2010, the annual cost of illness (COI) of MS in Sweden, from a societal perspective, was approximately EUR 414 million[3]. Productivity losses[3] and the cost of disease-modifying therapies (DMT) were the two main cost drivers identified[4].

The COI of MS has been shown to increase with disease's disability progression[4]. On the other hand, treatment with DMTs, has been associated with slowing this progression; plausibly also reducing the increase in the COI in MS over time[5].

Until now, only a few observational studies have been conducted evaluating the COI progression in MS in relation to DMTs. However, these studies do not have a longitudinal design[6], and have also not accounted for disability progression.

The aim of this study was to explore the COI progression in MS among newly diagnosed people with MS (PwMS) in Sweden, in relation to the first DMT received (interferons (IFN) vs. glatiramer acetate (GA) vs. natalizumab (NAT)), adjusting for disability progression over time.

For peer review only - http://bmjopen.bmj.com/site/about/guidelines.xhtml

Methods

Study design and data sources

This was a register-based prospective cohort study of PwMS in Sweden who fulfilled the following two inclusion criteria:

- according to the Swedish MS register (SMSreg)[7] had their first MS diagnosis in one of the years 2006-2015 (index year), when aged 20-55, and lived in Sweden when first diagnosed, (maximum age of 55 was chosen to allow follow-up until ordinary retirement age of 65)
- received IFN, GA, or NAT (DMTs licensed for MS treatment in Sweden during the study period), within 1 month after diagnosis, and they stayed on the therapy for at least 6 months.

Never-users of DMTs, those not on IFN/GA/NAT within 1 month from MS diagnosis, and/or not receiving it for at least 6 months after treatment initiation, and those receiving a DMT before MS diagnosis, were not included in the study.

All included PwMS were followed through 2016 in nationwide registers[7, 8]. The following anonymised microdata, kept by four authorities, were used:

- Stockholm Region:
  - Swedish MS register (SMSreg)[7]: diagnosis age and date, type of MS, Expanded Disability Status Scale (EDSS), and type(s) and dates of DMT treatment(s)
- National Board of Health and Welfare:
  - National Patient Register (NPR)[8]: dates and diagnoses for inpatient and specialized outpatient healthcare (i.e., secondary healthcare)
  - Swedish Prescribed Drug Register (SPDR)[8]: dates, names, and costs for all prescribed drugs dispensed from pharmacies

For peer review only - http://bmjopen.bmj.com/site/about/guidelines.xhtml

- Cause of Death Register[8]: year of death

- Statistics Sweden: Longitudinal Integration Database for Health Insurance and Labor Market Studies[8]; sex, birth year, educational level, country of birth, type of living area, family situation, annual income from work and/or benefits (yes/no)
- Swedish Social Insurance Agency: Micro Data for the Analysis of Social Insurance register[8]; dates, diagnoses, and grade (full- or part-time) of sickness absence (SA) in SA spells >14 days and disability pension (DP).

The registers were linked using the unique personal identity number that all residents in Sweden are assigned.

PwMS who switched from one DMT group to another relevant group before the 6-months cut-off, were allocated to the later DMT group. PwMS were censored at migration or death.

The following sociodemographic characteristics, measured at the year of diagnosis (index year), were considered: sex, age at index year, educational level, country of birth, type of living area, and family situation.

Four types of MS at diagnosis were identified, based on information available from SMSreg: Relapsing-Remitting MS (RRMS), Progressive-Relapsing MS (PRMS), Primary-Progressive MS (PPMS), and Secondary-Progressive MS (SPMS).

Multi-morbidity in the index year was derived utilizing the Rx-Risk Comorbidity Index[9], based on the type of drugs prescribed to PwMS according to SPDR.

Disability in MS was based on information from the Expanded Disability Status Scale (EDSS) available. The score of EDSS ranges from 0 to 10, with 0.5 step intervals (0 indicating no impairment, while 10 indicating death from MS)[10]. A clinically meaningful change in the EDSS score is a change of at least one point in patients with EDSS <5.5, and 0.5 point for those with EDSS of ≥5.5[11].

For peer review only - http://bmjopen.bmj.com/site/about/guidelines.xhtml

Where multiple EDSS scores for each calendar year were available, the highest EDSS value was retained. In case of missing EDSS scores in SMSreg, the mean annual score was used. This was computed from the scores of PwMS in the same DMT group, index year, and with the same type of MS. This method of imputation was chosen to account for individuals who had multiple missing EDSS values during the study follow-up.

Study outcomes

Healthcare resources consumed by PwMS as well as their SA and DP days were calculated and multiplied with unit costs (Table 1) to determine the societal COI of MS. The estimated COI included all such costs occurring for PwMS during a calendar year, irrespective of the main diagnosis behind the cost.

Inpatient and outpatient costs were calculated for each year of follow-up, by multiplying the number of days/visits, derived from NPR, with the cost per 1.0 retrospective nationwide weight from the diagnosis-related groups (DRG) system. Co-payment costs for these visits were also estimated by summing daily/per visit fees for inpatient and outpatient care. We assumed that the reimbursement periods started on 1 January, so costs exceeding the co-payment ceiling in Sweden for one year's period, were set to the maximum amount allowed per year (Table 1).

The cost of prescribed drugs was derived from the SPDR, based on the pharmacy's retail price of each dispensed drug package, including the co-payment paid by patients. The annual cost of drugs was calculated by summing the costs for all dispensed drugs during each year of follow-up.

The cost of DMTs not available through SPDR, i.e., MS drugs administered in the hospital setting and not dispensed by pharmacies (natalizumab and rituximab for the study period), was calculated using information in the SMSreg. The number of months on these treatments was multiplied with the cost per month of treatment (Table 1).

For peer review only - http://bmjopen.bmj.com/site/about/guidelines.xhtml

By adding costs of inpatient and outpatient care and co-payments for inpatient and outpatient care and drugs, the total annual healthcare costs per patient were calculated. Productivity losses were estimated based on the net days with SA and/or DP per year of follow-up, using the human capital approach[12]. Net months were multiplied with the age-adjusted mean salary, adding social security contributions made by employers (Table 1).

All people living in Sweden with income from work or unemployment benefits are from 16 years of age covered by the public SA insurance system and can be granted SA benefits if their work capacity is reduced due to disease or injury. The employer reimburses income loss during days 2-14 of a SA spell, after which SA benefits are administered by the Swedish Social Insurance Agency; for unemployed individuals, this happens from day 2. Therefore, to prevent bias in relation to employment status, only SA >14 days was included.

All people aged 19-65 years can be granted DP if their work capacity is long-term or permanently reduced[13]. Both SA and DP can be granted for full-time (100%) or part-time (75%, 50%, or 25%) of ordinary work hours[13], making it possible to have both partial SA and DP concurrently. Hence, we combined them to calculate the net days of SA and DP/year.

All costs were inflated to 2022 prices, applying the harmonized index for consumer prices[14]. Costs calculated in Swedish Krona (SEK) were then converted to Euros (EUR) in 2022 values (Table 1).

Table 1. Unit costs used in the calculation of healthcare costs and productivity losses

	Year	Value in 2022 SEK	Value in 2022 Euros[1]	Source
Average inpatient and outpatient cost per 1.0 DRG	2006	52,721 kr	4,960 €	Swedish Association of Local Authorities and Regions [Sveriges Kommuner och Regioner], KPP Somatik vård [15]
	2007	51,946 kr	4,887 €	
	2008	53,151 kr	5,000 €	
	2009	53,561 kr	5,039 €	
	2010	52,187 kr	4,910 €	
	2011	52,010 kr	4,893 €	

For peer review only - http://bmjopen.bmj.com/site/about/guidelines.xhtml

	2012	51,529 kr	4,848 €	
	2013	53,233 kr	5,008 €	
	2014	55,218 kr	5,195 €	
	2015	57,720 kr	5,430 €	
	2016	59,300 kr	5,579 €	
Co-payment for hospital stay (cost per day of stay)	2018	104 kr	10 €	Assume 100 SEK per day, as this is the case for the majority of the regions (including Stockholm).[16] The max co-payment amount for inpatient care was set to 1500 SEK per year (assumption for whole Sweden, based on information from the region Västra Götaland)[17]
Co-payment for visit in specialised care (cost per visit)	2018	284 kr	28 €	Swedish Association of Local Authorities and Regions (18). The max co-payment amount for outpatient care was set to 1100 SEK per year. Only one region in Sweden has a max co-payment less than 1100 SEK; so it was assumed 1100 SEK for the entire country. Swedish Association of Local Authorities and Regions[16]
Cost per month for natalizumab	2018	1,144 kr	111 €	Treatment with natalizumab is every 4th week (i.e. one per month)[18]; therefore, the cost per month of natalizumab was assumed to be the price for one dose of natalizumab (pharmacy's retail price)[18], excluding the cost of administration, which is captured in this study as an outpatient visit to the hospital
Cost per month for rituximab	2018	2,022 kr	197 €	Rituximab is used off-label in the treatment of MS; therefore, the exact treatment dosing was not available in the Swedish guidelines for MS treatment. A recent published study in Sweden regarding the use of rituximab for PwMS indicated that the drug dose is 500mg to 1000mg per treatment regime (here we assumed the mean, i.e 750mg per treatment regime), and the mean treatment interval for RRMS PwMS was 7.2 months per year[19]. The cost per month was then assumed to be 1.5 times the pharmacy's retail price per 500mg of rituximab injection, which was taken from FASS (9703.61 SEK)[20], divided with the frequency of treatment regimes in months (frequency: every 7.2 months) [19].
Monthly salary including employer contributions	2006	42,334	4,127 €	The average age-adjusted monthly wage (2018 values) for all employment types was retrieved[21]. It was multiplied with the annual employer contributions, available from the Swedish Tax Authority[22]. The final
	2007	42,457	4,139 €	
	2008	41,416	4,037 €	
	2009	46,112	4,495 €	

For peer review only - http://bmjopen.bmj.com/site/about/guidelines.xhtml

	2010	45,591	4,444 €	salary was then inflated to 2022 prices using annual Harmonised Indices of Consumer Prices [HICP] for healthcare available from Eurostat[14].
	2011	46,264	4,510 €	
	2012	46,283	4,512 €	
	2013	46,596	4,542 €	
	2014	47,701	4,650 €	
	2015	52,025	5,072 €	
	2016	49,797	4,854 €	

[1]The annual exchange rate for 2022 from SEK to Euros that was used was 10.6296. Source: Eurostat, Annual Exchange Rates Euro/ECU.

For peer review only

Analyses

Sociodemographic characteristics, multi-morbidity, type of MS at diagnosis, MS disability progression, average COI per patient and year, including 95% confidence intervals (CI), were calculated for each of the three treatment groups.

Pearson's chi-squared test[23] and the likelihood ratio test[23] were used to explore whether the observed sociodemographic and multi-morbidity differences across the three DMT groups were statistically significant (p-value<0.05).

Two-tailed student's T-tests, with unequal variance[24] were used to assess the significance (p-value<0.05) of the disability and COI changes over time, within each treatment group, compared to baseline values (at diagnosis) and differences across the treatment groups.

A generalised linear model[25], with Poisson distribution for costs, was used to estimate mean cost per patient per year, and per group, and 95% CI of the estimation. The model was adjusted with the disability progression of PwMS over time, by setting the EDSS variable to the mean EDSS score across all the three groups for that year.

Patient and public involvement

None.

For peer review only - http://bmjopen.bmj.com/site/about/guidelines.xhtml

Results

In total, 3673 PwMS, receiving their first treatment with IFN (N=2696), GA (N=441), or NAT (N=536) between 2006-2015, were included. Sex, age distribution in the groups, educational level, family situation, country of birth, type of living area, and multi-morbidity (any) differed across the groups (p-value<0.05; Table 2). NAT had fewer people with any comorbidities (Table 2) vs. the IFN and GA groups. The baseline EDSS score was similar for the three groups (Supplementary Figure 1, in the Online Supplementary Material).

In Supplementary Table 1, in the Online Supplementary Material, the unadjusted mean per patient per year COI for MS is presented, over the 10-year follow-up, and for the three treatment groups. Figures 1a and b show the disability-adjusted healthcare costs and productivity losses. Disability-adjusted costs were also computed for all the cost components (Supplementary Figure 2a-f, in the Online Supplementary Material).

People on NAT had higher healthcare costs vs. the IFN and GA groups (p-value<0.05 for both comparisons), driven by the higher drug costs for this group (Supplementary Figure 2d, in the Online Supplementary Material). IFN and GA groups had similar healthcare costs and productivity losses (p-values>0.05). Productivity losses for NAT vs. GA were similar (p-value>0.05), while higher when compared to those on IFN (p-value>0.05), driven by the higher SA costs for the NAT group (p-value=0.055 vs the IFN group). NAT had the lowest DP costs vs. the other two groups at the start of follow-up, and there was a trend of lower DP costs compared to those for the GA group (p-value>0.05) over time.

For peer review only - http://bmjopen.bmj.com/site/about/guidelines.xhtml

Table 2. Sociodemographic and multi-morbidity characteristics at year of diagnosis (index year)

	Treatment with Interferons	Treatment with Glatiramer Acetate	Treatment with Natalizumab	Pearson's Chi-Square (p-value)	Log-likelihood test Chi-Square (p-value)
	N=2696	N=441	N=536		
	n (%)[a]	n(%)[a]	n(%)[a]		
**Sex**				7.49 (0.024)	7.70 (0.021)
Women	1882 (51.24)	333 (9.07)	364 (9.91)		
Men	814 (22.16)	108 (2.94)	172 (4.68)		
**Age at diagnosis (index year)**				107.26 (<.0001)	108.56 (<.0001)
20-25	321 (8.74)	45 (1.23)	114 (3.1)		
26-30	435 (11.84)	67 (1.82)	129 (3.51)		
31-35	465 (12.66)	65 (1.77)	114 (3.1)		
36-40	477 (12.99)	88 (2.4)	74 (2.01)		
41-45	463 (12.61)	75 (2.04)	46 (1.25)		
46-50	316 (8.6)	60 (1.63)	39 (1.06)		
51-55	219 (5.96)	41 (1.12)	20 (0.54)		
**Type of MS at diagnosis (index year)[b]**				2.24 (0.896)	2.25 (0.896)
RRMS	1960 (65.14)	302 (10.04)	419 (13.92)		
PPMS	23 (0.76)	4 (0.13)	4 (0.13)		
SPMS	187 (6.21)	31 (1.03)	33 (1.1)		
PRMS	31 (1.03)	6 (0.2)	9 (0.3)		
**Educational level[c]**				72.90 (<.0001)	58.09 (<.0001)
Elementary school	387 (10.84)	67 (1.88)	134 (3.75)		
High School	1290 (36.13)	210 (5.88)	225 (6.3)		
University/college	946 (26.5)	153 (4.29)	158 (4.43)		
**Country of birth[c]**				17.70 (0.0236)	14.70 (0.065)
Sweden	2348 (65.77)	390 (10.92)	454 (12.72)		
Nordic countries (except Sweden)	43 (1.2)	9 (0.25)	15 (0.42)		
EU27 (except Denmark, Finland, and Sweden)	42 (1.18)	8 (0.22)	16 (0.45)		
Rest of the world	190 (5.32)	23 (0.64)	32 (0.9)		
**Type of living area**				15.47 (0.004)	15.30 (0.004)
Big cities[c]	1020 (28.57)	142 (3.98)	210 (5.88)		

For peer review only - http://bmjopen.bmj.com/site/about/guidelines.xhtml

Medium sized cities	888 (24.87)	141 (3.95)	187 (5.24)		
Rural areas	715 (20.03)	147 (4.12)	120 (3.36)		
**Family situation**[c]				140.56 (<.0001)	123.74 (<.0001)
Married or cohabitant without children[d,e]	189 (5.29)	38 (1.06)	26 (0.73)		
Married or cohabitant with children[d,e]	1021 (28.6)	165 (4.62)	117 (3.28)		
Single without children[d,e]	1211 (33.92)	190 (5.32)	320 (8.96)		
Single with children[d,e]	174 (4.87)	28 (0.78)	25 (0.7)		
**Any multi-morbidity**				68.35 (<.0001)	53.92 (<.0001)
Yes	2580 (70.24)	424 (11.54)	466 (12.69)		
No	116 (3.16)	17 (0.46)	70 (1.91)		
**Anxiety/Depression**				4.10 (0.129)	3.91 (0.142)
Yes	137 (3.73)	28 (0.76)			
No	2559 (69.67)	413 (11.24)			

[a]The percentages are calculated as n divided with the total number of individuals in the study population (3673 if not otherwise indicated; see b)

[b]For 18% of the cohort, type of MS information was missing; so, information was available for 3009 PwMS in the study cohort

[c]The total number of individuals with this type of information in the study population (i.e., all individuals excluding those with missing information in this category) was 3570

[d]Only cohabitants with children in common are registered as cohabitants. Otherwise they were registered as single.

[e]Children were considered as being <18 years and living at home

For peer review only

Discussion

In this register-based prospective cohort study we explored the development of the COI of MS over time among newly diagnosed PwMS in Sweden. Costs were estimated in relation to what type of first line treatment was used; IFN, vs GA, vs NAT, with adjustment for MS disability progression. The analysis sub-groups were constructed based on the DMTs that were licensed to be used as 1st line therapies for MS in Sweden during 2006-2016, and for which we had information during the study's data period (2006-2016).

Overall, the general trends of decreasing healthcare costs and increasing productivity losses that were observed in this study are in line with previous COI studies in MS with a similar longitudinal design[26, 27]. No significant differences in healthcare costs were observed between the IFN and GA, while NAT had higher healthcare costs (p-value<0.05). This could be explained by the higher treatment cost with NAT[28], i.e., higher drug cost and healthcare utilization to administer and monitor PwMS on this DMT. As previous studies have shown, in the last two decades, the cost of drugs has become main COI driver in MS[4].

In addition, outpatient costs for PwMS on NAT could have played a role; they were higher for NAT (p-value>0.05). For the beginning of follow-up, until PwMS on NAT change therapy, this could be explained by that fact that these patients require at least one extra outpatient visit per month to get their treatment[29]. Before treatment, additional diagnostic tests (blood tests and in cases MRI scans) have to be performed, not needed for PwMS on other therapies, including IFN and GA[29]. Therefore, when compared to IFN and GA, healthcare costs for NAT could be amplified due to the extra outpatient visits needed for the treatment itself. Had no such visits and tests been necessary, outpatient costs NAT could have been at the same level as the ones for IFN and GA, or even lower.

For peer review only - http://bmjopen.bmj.com/site/about/guidelines.xhtml

Furthermore, DMTs were not randomly assigned to PwMS in our observational study, and therefore this study could not consider treatment decisions based on observed disease activity at the time of diagnosis. If treating neurologists assigned NAT to PwMS who could have had potentially higher disease activity, trying to prevent worse disease outcomes, this may have resulted in greater healthcare costs in the NAT group vs. IFN or GA, than those that could be expected if DMTs were randomly assigned to PwMS.

Towards the end of follow-up, when people may have switched to other DMTs, or stopped treatment, other factors could be associated with the observed healthcare cost differences, which have not been covered in this study, such as the progression of MS symptoms, relapses, and multi-morbidity.

Productivity losses for NAT and GA were similar; both had higher productivity losses vs. IFN (p-value<0.05). Since the comparative effectiveness of IFN and GA is similar[30], other unmeasured factors than disease progression, such as fatigue and multi-morbidity could explain this finding. On the other hand, even if treatment with NAT can lead to lower relapse rates[31], and hence possibly to slower disease progression, compared to IFN and GA, productivity losses for this group were higher. The need to take time off work when on NAT related to the administration of the therapy (outpatient visits to administer the therapy and run diagnostic tests), and recovery from the administration of this DMT, could explain part of this observed outcome[29], since it adds extra SA costs for NAT[32].

In fact, only SA costs were higher for NAT compared to the other groups; DP costs were lower than GA, indicating that even if PwMS receiving NAT as first-line treatment, are having higher MS disability at baseline, and probably higher disease activity compared to those receiving IFN or GA[29], still, their ability to maintain their long-term work capacity is higher.

For peer review only - http://bmjopen.bmj.com/site/about/guidelines.xhtml

Experiencing less relapses and in general less MS symptoms like fatigue, which can allow patients to maintain employment longer[33].

Previously published health economic models presenting the cost progression related to these three DMTs have often been criticised due to their limited data availability as well as the methodological complexities and uncertainties[33]. The COI of MS associated with new generation therapies, like NAT, compared to older therapies (IFN and GA) has not been studied so far involving longitudinal COI data with multiple years of follow-up for costs and disease progression.

Strengths and limitations

Main strengths of this study are its prospective cohort design, with several years of follow-up, and that data from high-quality administrative registers could be used. All PwMS in the SMSreg diagnosed with MS in the years 2006-2015 and receiving first-line therapy with NAT, IFN, or GA could be followed for several years in nationwide registers, eliminating drop outs and recall biases when using self-reported information from PwMS[4]. However, as there are no nationwide register data in Sweden regarding primary healthcare, rehabilitation, home help, home investments to improve mobility, and other out of pocket expenses this could not be included in our COI calculations. In addition, this study does not take into account the societal costs associated to premature death due to the disease. Hopefully future studies can include such information, to analyse how MS symptoms and multi-morbidity might be associated with the healthcare costs and overall societal burden in MS, based on the DMT treatment administered.

In addition, the longest follow-up time we had was ten years after diagnosis; while this time can give important information regarding the COI progression for the three treatment groups, only very few patients had this many follow-up years. Therefore, the longer the follow-up, the higher the uncertainty is for the estimated COI presented. Moreover, while the potential

For peer review only - http://bmjopen.bmj.com/site/about/guidelines.xhtml

For peer review only

effect of the first-line therapy on the COI can be observed for the time PwMS are receiving that treatment and maybe for a short period of time afterwards, also other factors could be strongly associated with the COI progression over time after the first-line therapy was stopped, not measured in this study (eg, type of second-line therapy, adherence to treatment, etc).

Another important strength is that robust information on productivity losses was available enabling us to apply the societal perspective on the COI calculations. Productivity losses are the main long-term driver of the COI in MS as shown in this and previous studies[4]. However, short-term SA-spells of 14 days or less were not quantified possibly leading to underestimation of the productivity losses. Short-term SA spells have not been considered for both employed (including self-employed) and unemployed patients, hence no bias based on employment status has been introduced in the calculation of productivity losses.

Moreover, any productivity reductions while being present at work that could potentially be related to the presence of MS were not quantified. Furthermore, productivity losses incurred by partners of people with MS, i.e., informal care costs, were not included in the COI calculations; while measuring these costs was beyond the aim of this study, they are an important cost component when defining the overall COI of MS[4].

Similar to what previous COI in MS studies have done[4], we used the EDSS score to explore disability progression for PwMS in the three treatment groups. However, when EDSS information was missing, information had to be imputed. This has likely diluted the EDSS score used in the adjusted COI analysis. In addition, EDSS has been primarily developed and used for research purposes, not for clinical or treatment decisions in MS, and its scores are less effectively tracking disease progression over time[34]. Also, some MS symptoms, like fatigue, which are important and can limit the everyday activities of PwMS early in the disease trajectory, are not

For peer review only - http://bmjopen.bmj.com/site/about/guidelines.xhtml

well detailed in the EDSS assessment[34], obtaining in this way a skewed result regarding the disability of PwMS. Additional clinical measures could have complemented our analysis.

Another limitation is that the generalizability of our findings can be limited. The study cohort was taken from SMSreg, which back in 2006 had approximately 50% coverage of all PwMS in Sweden, and 80% in 2015[35]. Assuming no distinct differences in the sociodemographic characteristics, multi-morbidity, and disability with the PwMS that our study did not include, our findings could be generalizable to all PwMS in Sweden. Still, in our cohort, an elevated number of people with SPMS at diagnosis was included vs. the expected distribution of the type of MS at diagnosis, which could potentially hinder the generalizability of our findings. In addition, generalization of our findings to other countries is not possible, considering the differences in the organisation of healthcare and social security systems.

Conclusions

Using real-world data from Swedish nationwide registers, this study explored the association between the type of first-line DMT received (IFN vs. GA vs. NAT) with COI progression. The findings of a trend towards lower DP costs indicated that PwMS on NAT could potentially maintain their work capacity for a longer time. COI serves as an objective measure showing the importance of the use of second-generation treatments in maintaining at low levels the progression of MS over time, and hence the overall COI.

For peer review only - http://bmjopen.bmj.com/site/about/guidelines.xhtml

Data availability statement: The data used in this study is administered by the Division of Insurance Medicine, Karolinska Institutet, and cannot be made publicly available. According to the General Data Protection Regulation, the Swedish law SFS 2018:218, the Swedish Data Protection Act, the Swedish Ethical Review Act, and the Public Access to Information and Secrecy Act, these types of sensitive data can only be made available, after legal review, for researchers who meet the criteria for access to this type of sensitive and confidential data. Readers may contact Professor Kristina Alexanderson (kristina.alexanderson@ki.se) regarding the data.

Contributors: All authors (Korinna Karampampa, KK; Hanna Gyllensten, HG; Emilie Friberg, EF; Chantelle Murley, CM; Jan Hillert, JH; Andrius Kavaliunas, AK; Tomas Olsson, TO; Kristina Alexanderson, KA) contributed to the conceptualization of the research questions, the study design and methods. KK performed the analysis of the data, interpreted the study findings, and drafted the manuscript. All remaining authors (HG, EF, CM, JH, AK, TO, KA) assisted in the interpretation of the study findings and contributed with comments/suggestions and text to the manuscript.

Patient consent for publication: Not required.

Ethics approval: The project received ethical approval from the Regional Ethical Review Board in Stockholm. Approval numbers: 2007/762-31; 2009/23-32; 2009/1917-32; 2010/466-32; 2011/806-32; 2011/1710-32; and 2014/236-32.

Funding: This study was supported by an unrestricted investigator-initiated trial grant from Biogen to support the conduct of this study (Award number N/A). We utilised data from the REWHARD consortium supported by the Swedish Research Council (grant number 2021-00154).

For peer review only - http://bmjopen.bmj.com/site/about/guidelines.xhtml

Competing interests: All authors (KK, HG, EF, CM, JH, AK, TO, KA) are employed or affiliated at the Department of Clinical Neuroscience, Karolinska Institutet, Stockholm, Sweden. KK is currently employed by Celgene/Bristol Myers Squibb; she initiated this study while being employed at Karolinska Institutet (employment ended in October 2019); since then, she has received no salary from Karolinska Institutet or other type of funding for this research. HG is currently employed part-time by Statfinn/EPID Research (which is part of IQVIA); both companies are contract research organizations that perform commissioned pharmacoepidemiological studies, and therefore are collaborating with several pharmaceutical companies. CM since submission of this paper has begun employment with Macanda AB. AK is currently also employed by Takeda Pharma AB. JH, KA, and EF are collaborating with several pharmaceutical companies; EF has received an unrestricted MS research grant from Celgene/Bristol Myers Squibb .TO has received advisory board and/or lecture honoraria, and unrestricted MS research grants from Biogen, Novartis, Sanofi, Merck and Roche.

For peer review only - http://bmjopen.bmj.com/site/about/guidelines.xhtml

References

1. Dutta R and Trapp BD. Mechanisms of neuronal dysfunction and degeneration in multiple sclerosis. *Prog Neurobiol*. 2011; 93: 1-12.
2. Ahlgren C, Oden A and Lycke J. High nationwide incidence of multiple sclerosis in Sweden. *PLoS One*. 2014; 9: e108599.
3. Gyllensten H, Wiberg M, Alexanderson K, et al. Costs of illness of multiple sclerosis in Sweden: a population-based register study of people of working age. *Eur J Health Econ*. 2017.
4. Ernstsson O, Gyllensten H, Alexanderson K, Tinghög P, Friberg E and Norlund A. Cost of Illness of Multiple Sclerosis - A Systematic Review. *PLoS One*. 2016; 11: e0159129.
5. Cerqueira JJ, Compston DAS, Geraldes R, et al. Time matters in multiple sclerosis: can early treatment and long-term follow-up ensure everyone benefits from the latest advances in multiple sclerosis? *J Neurol Neurosurg Psychiatry*. 2018; 89: 844-50.
6. Iannazzo S, Iliza AC and Perrault L. Disease-Modifying Therapies for Multiple Sclerosis: A Systematic Literature Review of Cost-Effectiveness Studies. *Pharmacoeconomics*. 2018; 36: 189-204.
7. Hillert J and Stawiarz L. The Swedish MS registry - clinical support tool and scientific resource. *Acta neurologica Scandinavica*. 2015; 132: 11-9.
8. Ludvigsson JF, Almqvist C, Bonamy AK, et al. Registers of the Swedish total population and their use in medical research. *Eur J Epidemiol*. 2016; 31: 125-36.
9. Pratt NL, Kerr M, Barratt JD, et al. The validity of the Rx-Risk Comorbidity Index using medicines mapped to the Anatomical Therapeutic Chemical (ATC) Classification System. *BMJ Open*. 2018; 8: e021122.
10. Collins CD, Ivry B, Bowen JD, et al. A comparative analysis of Patient-Reported Expanded Disability Status Scale tools. *Mult Scler*. 2016; 22: 1349-58.
11. Goodkin DE. EDSS reliability. *Neurology*. 1991; 41: 332.
12. Drummond MF, Sculpher MJ, Claxton K, Stoddart GL and Torrance GW. *Methods for the economic evaluation of health care programmes*. Oxford university press, 2015.
13. National Insurance Agency [Försäkringskassan]. Social Insurance in Figures 2017. 2017.
14. Eurostat. HICP - all items - annual average indices (2015=100). https://ec.europa.eu/eurostat/tgm/table.do?tab=table&init=1&language=en&pcode=tec00027&plugin=12019.
15. Swedish Association of Local Authorities and Regions [Sveriges Kommuner och Landsting]. Economic statistics - KPP Somatik - SKL. https://skl.se/ekonomijuridikstatistik/statistik/kostnadperpatientkpp/kppsomatik.1077.html2019.
16. Swedish Association of Local Authorities and Regions [Sveriges Kommuner och Landsting]. Economic statistics - Patientavgifter fr.o.m. den 1 januari 2018. https://skl.se/download/18.3ee4cc4f1611af77b89179dd/1517495154691/avgift_%C3%B6ppenv%C3%A5rd_inkl.missiv_slutenavg18_19jan18.pdf2018.
17. Vårdguiden 1177. Patient fees in Västra Götaland county in Sweden [Patientavgifter i Västra Götaland]. https://www.1177.se/Vastra-Gotaland/Regler-och-rattigheter/Patientavgifter-i-Vastra-Gotaland/2018.
18. FASS.se. Tysabri. https://www.fass.se/LIF/product?nplId=20040916001115&userType=0&docType=6&scrollPosition=751.3333129882812019.
19. Salzer J, Svenningsson R, Alping P, et al. Rituximab in multiple sclerosis: A retrospective observational study on safety and efficacy. *Neurology*. 2016; 87: 2074-81.

For peer review only - http://bmjopen.bmj.com/site/about/guidelines.xhtml

20. FASS.se. Rituximab - price. https://www.fass.se/LIF/product?userType=2&nplId=19980602000029&docType=30&scrollPosition=600s2019.
21. Statistics Sweden. Genomsnittlig månadslön 1973–2018. https://www.scb.se/hitta-statistik/statistik-efter-amne/arbetsmarknad/loner-och-arbetskostnader/lonestrukturstatistik-privat-sektor-slp/pong/tabell-och-diagram/genomsnittlig-manadslon-1973-/2019.
22. Swedish Tax Authority [Skatteverket]. Belopp och procent för åren 2019-2004 - privat. https://www.skatteverket.se/privat/skatter/beloppochprocent.4.18e1b10334ebe8bc80004109.html 2019.
23. Moore DS. Tests of chi-squared type. *Goodness-of-fit-techniques*. Routledge, 2017, p. 63-96.
24. Kim TK. T test as a parametric statistic. *Korean journal of anesthesiology*. 2015; 68: 540-6.
25. McCullagh P and Nelder J. Generalized linear models., 2nd edn.(Chapman and Hall: London). *Standard book on generalized linear models*. 1989.
26. Gyllensten H, Kavaliunas A, Murley C, et al. Costs of illness progression for different multiple sclerosis phenotypes: A population-based study in Sweden. *Submitted*. 2018.
27. Karampampa K, Gyllensten H, Yang F, et al. Healthcare, Sickness Absence, and Disability Pension Cost Trajectories in the First 5 Years After Diagnosis with Multiple Sclerosis: A Prospective Register-Based Cohort Study in Sweden. *Pharmacoecon Open*. 2019.
28. Hartung DM, Bourdette DN, Ahmed SM and Whitham RH. The cost of multiple sclerosis drugs in the US and the pharmaceutical industry: Too big to fail? *Neurology*. 2015; 84: 2185-92.
29. Piehl F, Olsson T, Lycke J, et al. Läkemedelsbehandling av multipel skleros (MS)–bakgrundsdokumentation. https://lakemedelsverket.se/upload/halso-och-sjukvard/behandlingsrekommendationer/bakg_dok/Lakemedelsbehandling_av_multipel_skleros_MS_bakgrundsdokumentation_webb.pdf.
30. Melendez-Torres GJ, Armoiry X, Court R, et al. Comparative effectiveness of beta-interferons and glatiramer acetate for relapsing-remitting multiple sclerosis: systematic review and network meta-analysis of trials including recommended dosages. *BMC Neurol*. 2018; 18: 162.
31. Spelman T, Kalincik T, Jokubaitis V, et al. Comparative efficacy of first-line natalizumab vs IFN-beta or glatiramer acetate in relapsing MS. *Neurol Clin Pract*. 2016; 6: 102-15.
32. Kwiatkowski A, Marissal JP, Pouyfaucon M, Vermersch P, Hautecoeur P and Dervaux B. Social participation in patients with multiple sclerosis: correlations between disability and economic burden. *BMC Neurol*. 2014; 14: 115.
33. Glanz BI, Degano IR, Rintell DJ, Chitnis T, Weiner HL and Healy BC. Work productivity in relapsing multiple sclerosis: associations with disability, depression, fatigue, anxiety, cognition, and health-related quality of life. *Value Health*. 2012; 15: 1029-35.
34. Meyer-Moock S, Feng YS, Maeurer M, Dippel FW and Kohlmann T. Systematic literature review and validity evaluation of the Expanded Disability Status Scale (EDSS) and the Multiple Sclerosis Functional Composite (MSFC) in patients with multiple sclerosis. *BMC Neurol*. 2014; 14: 58.
35. Swedish Neuroregistries MS. Visualisation and Analysis platform (VAP) for Multiple Sclerosis - Coverage ratio broken down by county, gender and the last follow-up date [Täckningsgrad uppdelat för län, kön och sista uppföljningsdatum], Coverage ratio against the patient register, by year [Täckningsgrad mot patientregistret, år].

For peer review only - http://bmjopen.bmj.com/site/about/guidelines.xhtml

https://www.neuroreg.se/en.html/multiple-sclerosis-realtime-data-and-results-vap: Swedish Neuroregistries - Multiple Sclerosis, 2019.

For peer review only

For peer review only - http://bmjopen.bmj.com/site/about/guidelines.xhtml

Figure 1. Healthcare costs (A) and productivity losses (B) (estimated mean from the regression) from baseline (year at MS diagnosis) to the end of follow-up, by treatment groups, adjusted for disability progression during the follow-up

For peer review only

For peer review only - http://bmjopen.bmj.com/site/about/guidelines.xhtml

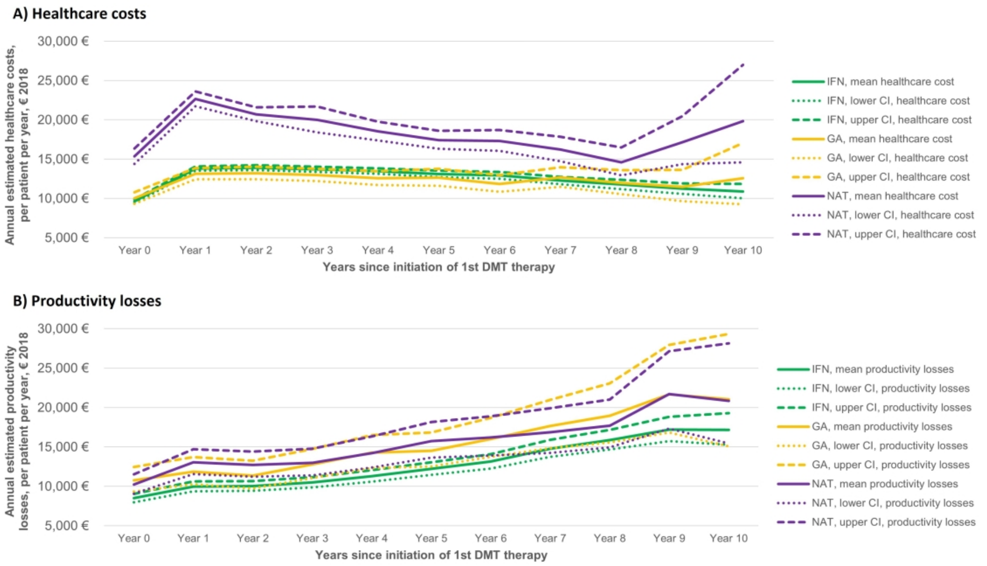

Healthcare costs (A) and Productivity losses (B) (estimated mean from the regression) from baseline (year at MS diagnosis) to the end of follow-up, by treatment groups, adjusted for disability progression during the follow-up

294x170mm (330 x 330 DPI)

Online Supplementary Material

Supplementary Table 1: Mean costs per patient per year, mean costs [95% confidence intervals] in € 2022 prices (crude observed means and confidence intervals, unadjusted), by treatment groups

	**Year 0 (year of MS diagnosis)**	**Year 1**	**Year 2**	**Year 3**	**Year 4**	**Year 5**	**Year 6**	**Year 7**	**Year 8**	**Year 9**	**Year 10**
**Interferon group**	**N=2696**	**N=2696**	**N=2644**	**N=2495**	**N=2219**	**N=1879**	**N=1556**	**N=1260**	**N=957**	**N=627**	**N=320**
Inpatient costs	1774 [1631-1918]	1079 [923-1236]	1327 [1147-1508]	1236 [1068-1405]	1148 [975-1321]	1241 [1022-1460]	1281 [1053-1509]	1082 [872-1291]	1439 [1004-1874]	1006 [707-1304]	1042 [608-1477]
Outpatient costs	1800 [1747-1853]	1368 [1313-1424]	1387 [1329-1446]	1417 [1352-1482]	1444 [1378-1510]	1528 [1453-1604]	1537 [1454-1619]	1626 [1510-1742]	1635 [1532-1738]	1726 [1594-1858]	1754 [1572-1935]
Co-payments	107 [106-109]	74 [72-75]	69 [67-71]	66 [64-68]	66 [64-68]	67 [65-69]	67 [64-69]	66 [64-69]	67 [64-70]	65 [61-69]	69 [63-74]
Drug costs	5904 [5733-6074]	11170 [10984-11357]	11029 [10811-11247]	10843 [10596-11090]	10686 [10412-10961]	10113 [9805-10421]	9850 [9504-10196]	9361 [8974-9748]	8624 [8176-9072]	8394 [7835-8954]	7927 [7120-8735]
**Total healthcare costs**	9585 [9345-9826]	13701 [13439-13962]	13813 [13512-14114]	13562 [13243-13882]	13345 [13001-13688]	12948 [12551-13346]	12734 [12303-13165]	12135 [11672-12598]	11765 [11145-12385]	11191 [10540-11842]	10792 [9892-11693]
SA costs	6927 [6469-7386]	7816 [7263-8369]	6586 [6078-7094]	5320 [4849-5790]	4649 [4167-5131]	4446 [3955-4937]	4113 [3595-4630]	3966 [3403-4529]	4410 [3688-5131]	3988 [3199-4777]	3896 [2837-4955]
DP costs	1890 [1555-2225]	2547 [2168-2926]	3887 [3433-4341]	5614 [5069-6159]	7386 [6730-8042]	8590 [7832-9348]	10068 [9172-10965]	11944 [10865-13023]	12781 [11501-14062]	14792 [13118-16466]	15204 [12888-17519]
**Total productivity losses**	8817 [8273-9362]	10356 [9709-11004]	10473 [9818-11128]	10934 [10244-11623]	12035 [11259-12811]	13036 [12175-13897]	14181 [13184-15177]	15910 [14747-17073]	17191 [15804-18578]	18780 [17023-20536]	19100 [16687-21512]
**Glatiramer Acetate group**	**N=441**	**N=441**	**N=430**	**N=406**	**N=348**	**N=292**	**N=225**	**N=170**	**N=116**	**N=66**	**N=31**
Inpatient costs	2217 [1733-2702]	1323 [862-1784]	1183 [775-1590]	1136 [731-1540]	1429 [956-1902]	1538 [911-2165]	880 [445-1315]	1401 [827-1974]	1324 [602-2047]	550 [134-967]	1417 [-911-3744]
Outpatient costs	2042 [1894-2189]	1506 [1361-1652]	1510 [1369-1651]	1566 [1407-1726]	1627 [1428-1826]	1716 [1505-1927]	1709 [1478-1940]	1734 [1475-1993]	1995 [1555-2435]	2165 [1573-2758]	2739 [1162-4316]
Co-payments	109 [104-113]	79 [74-84]	73 [69-78]	72 [67-76]	71 [65-77]	75 [69-80]	70 [64-76]	77 [69-86]	74 [64-84]	71 [60-82]	73 [57-90]
Drug costs	5670 [5227-6112]	10292 [9803-10780]	10257 [9708-10805]	9891 [9305-10476]	9211 [8553-9868]	9026 [8257-9796]	8846 [7966-9726]	9281 [8243-10320]	8390 [7164-9616]	8467 [6740-10195]	8282 [5644-10921]
**Total healthcare costs**	10037 [9341-10733]	13235 [12498-13972]	13023 [12252-13794]	12664 [11915-13414]	12338 [11483-13192]	12355 [11316-13393]	11505 [10464-12546]	12493 [11267-13719]	11783 [10289-13277]	11254 [9312-13196]	12511 [8603-16420]

For peer review only

SA costs	8118 [6848-9389]	8681 [7229-10132]	7190 [5834-8546]	6649 [5300-7998]	6105 [4718-7492]	5155 [3768-6541]	5154 [3627-6680]	5067 [3422-6712]	5191 [2897-7485]	4605 [2407-6803]	3815 [656-6975]
DP costs	3329 [2218-4441]	4127 [2896-5359]	5312 [3915-6709]	7615 [5945-9285]	9754 [7778-11730]	11788 [9474-14103]	13142 [10471-15812]	15090 [11772-18407]	18078 [13827-22329]	18791 [13241-24341]	19044 [11703-26384]
**Total productivity losses**	11447 [9871-13024]	12882 [11104-14661]	12502 [10690-14314]	14264 [12271-16257]	15859 [13620-18097]	16943 [14442-19444]	18295 [15397-21194]	20157 [16747-23566]	23269 [18820-27719]	23396 [17673-29118]	22859 [15118-30600]
**Natalizumab group**	**N=536**	**N=536**	**N=453**	**N=379**	**N=315**	**N=260**	**N=197**	**N=153**	**N=118**	**N=65**	**N=26**
Inpatient costs	4862 [4202-5521]	3230 [2519-3942]	2317 [1753-2881]	3043 [1683-4403]	2290 [1549-3031]	1725 [1074-2376]	1642 [920-2363]	1836 [1045-2628]	1087 [475-1699]	2951 [1095-4806]	3302 [166-6438]
Outpatient costs	2662 [2475-2848]	2580 [2372-2788]	2252 [2037-2467]	2161 [1918-2404]	2492 [2188-2795]	2254 [2007-2501]	2202 [1904-2501]	2397 [2006-2788]	2227 [1853-2601]	2831 [2238-3425]	3027 [1930-4124]
Co-payments	135 [130-140]	102 [97-107]	87 [83-91]	87 [82-92]	88 [82-94]	90 [83-96]	82 [75-89]	86 [78-94]	78 [70-86]	92 [77-107]	94 [71-117]
Drug costs	7568 [7064-8072]	16023 [15485-16560]	15930 [15291-16569]	14550 [13770-15330]	13512 [12619-14405]	13295 [12277-14312]	13368 [12214-14522]	11826 [10469-13184]	11336 [9816-12856]	11197 [8998-13396]	13386 [8164-18608]
**Total healthcare costs**	15226 [14283-16170]	22588 [21691-23484]	20586 [19724-21447]	19840 [18220-21461]	18382 [17204-19560]	17364 [16240-18489]	17293 [16013-18573]	16147 [14604-17689]	14728 [12909-16546]	17071 [14005-20137]	19809 [13284-26335]
SA costs	9683 [8492-10875]	11855 [10312-13399]	9867 [8358-11376]	7751 [6306-9197]	7484 [5784-9184]	7023 [5309-8737]	5292 [3639-6946]	5402 [3574-7229]	4788 [2807-6770]	5343 [2248-8438]	3317 [287-6348]
DP costs	2327 [1498-3157]	3392 [2388-4396]	5034 [3727-6341]	7629 [5942-9317]	9239 [7241-11237]	12062 [9629-14495]	14303 [11429-17177]	15873 [12321-19425]	17963 [13694-22233]	19921 [13856-25986]	20632 [10822-30443]
**Total productivity losses**	12010 [10646-13375]	15241 [13486-16996]	14901 [13047-16755]	15381 [13343-17418]	16723 [14354-19091]	19085 [16427-21743]	19596 [16559-22632]	21275 [17620-24930]	22752 [18472-27032]	25264 [19184-31343]	23950 [14408-33491]

For peer review only

Supplementary Figure 1: EDSS score progression from baseline (index year) to the end of follow-up, by IFN vs GA vs NAT treatment groups

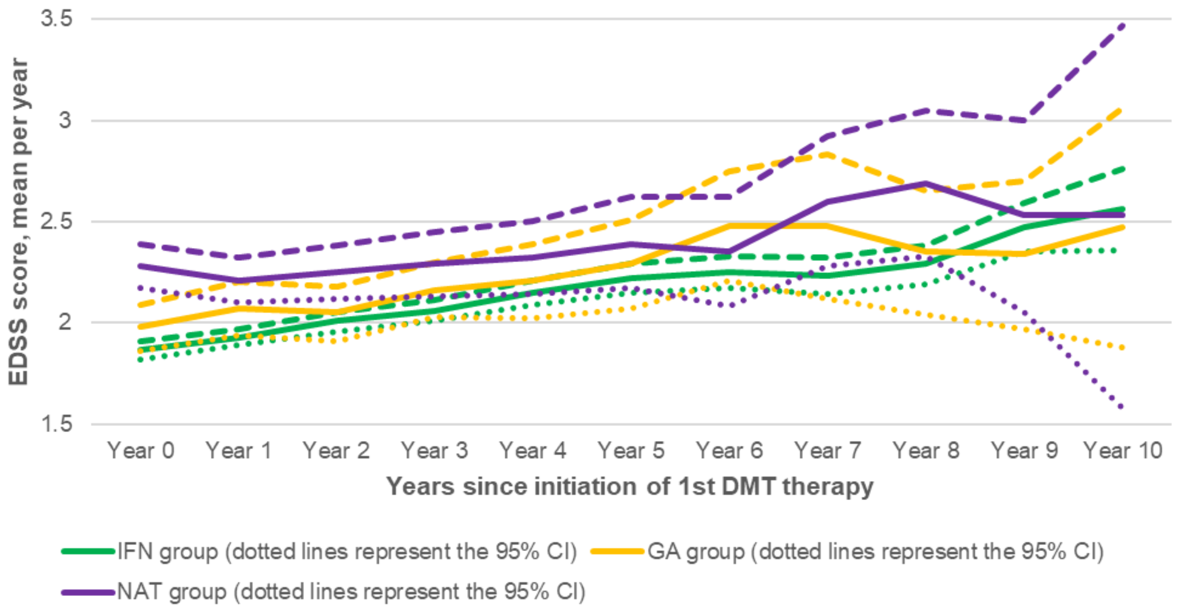

For peer review only

Supplementary Figure 2 (a-f): Progression (estimated mean from the regression) for all cost components, from baseline (year at MS diagnosis) to the end of follow-up, by treatment groups, adjusted for disability progression during follow-up

A) Inpatient costs

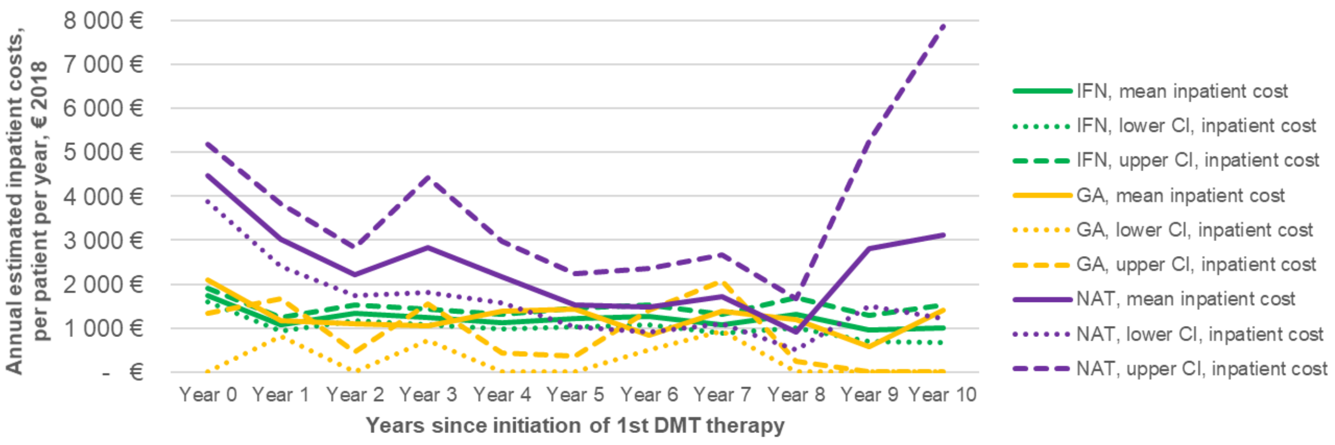

B) Outpatient costs

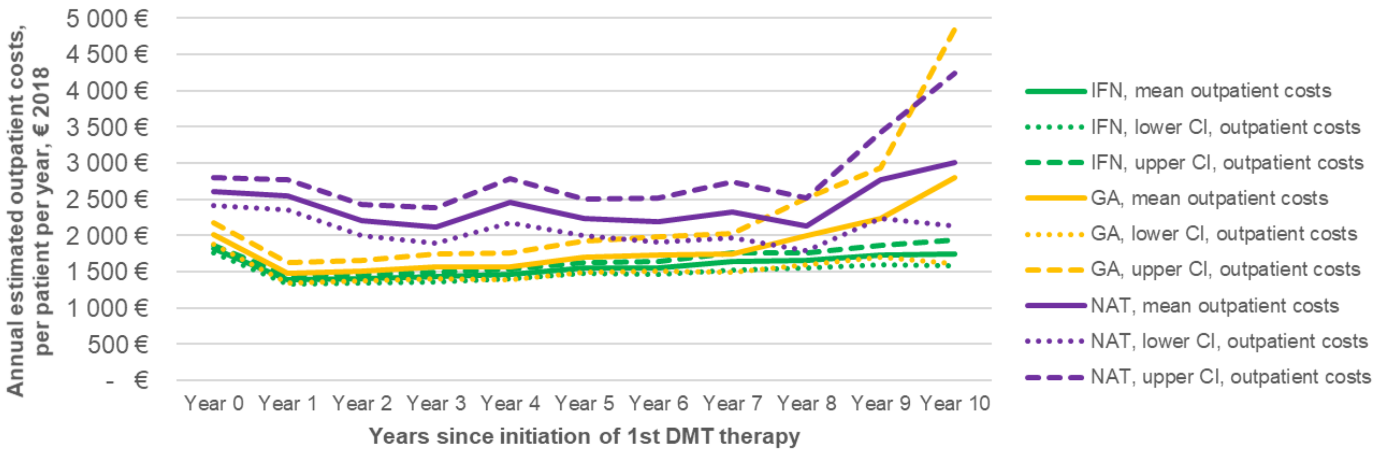

C) Co-payments

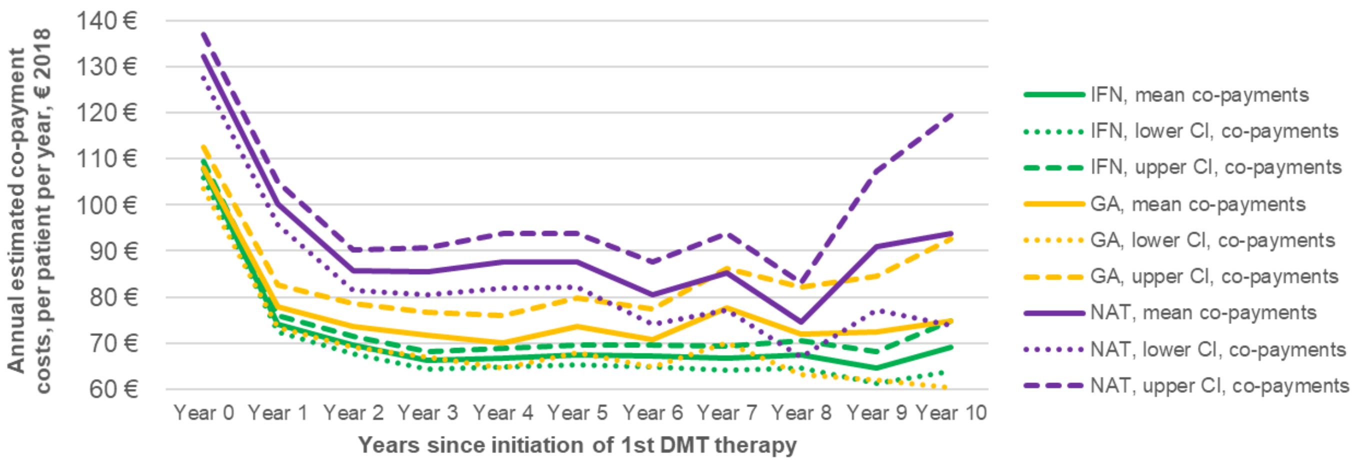

D) Drug costs

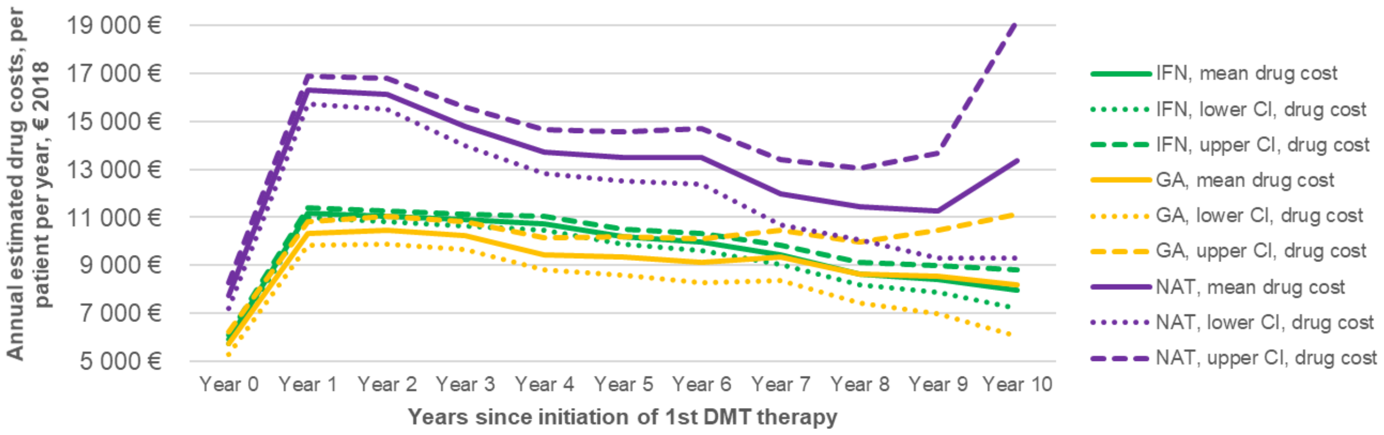

E) Sickness absence costs

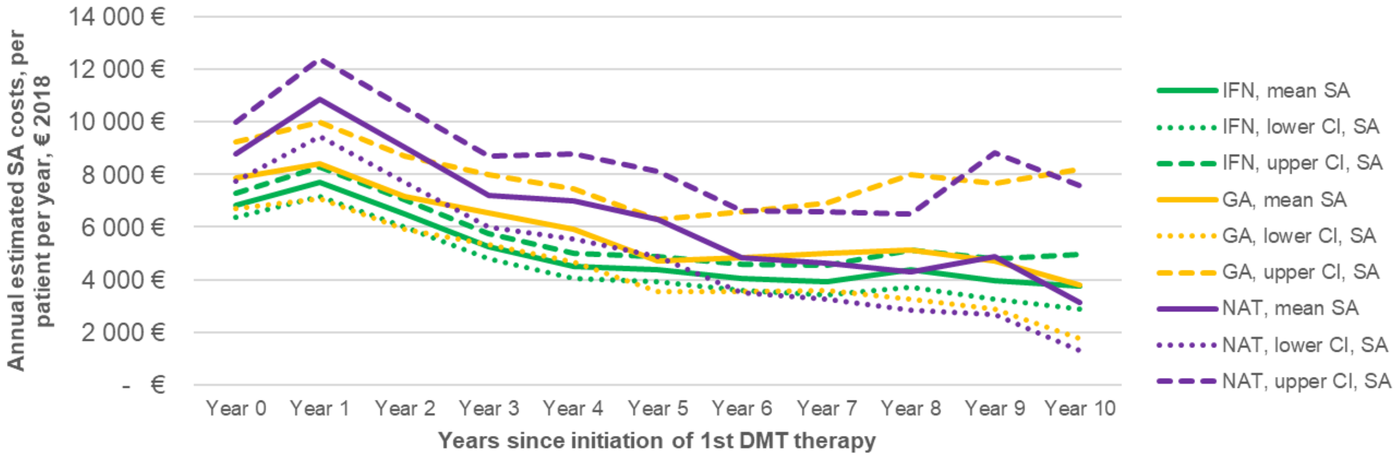

F) Disability pension costs

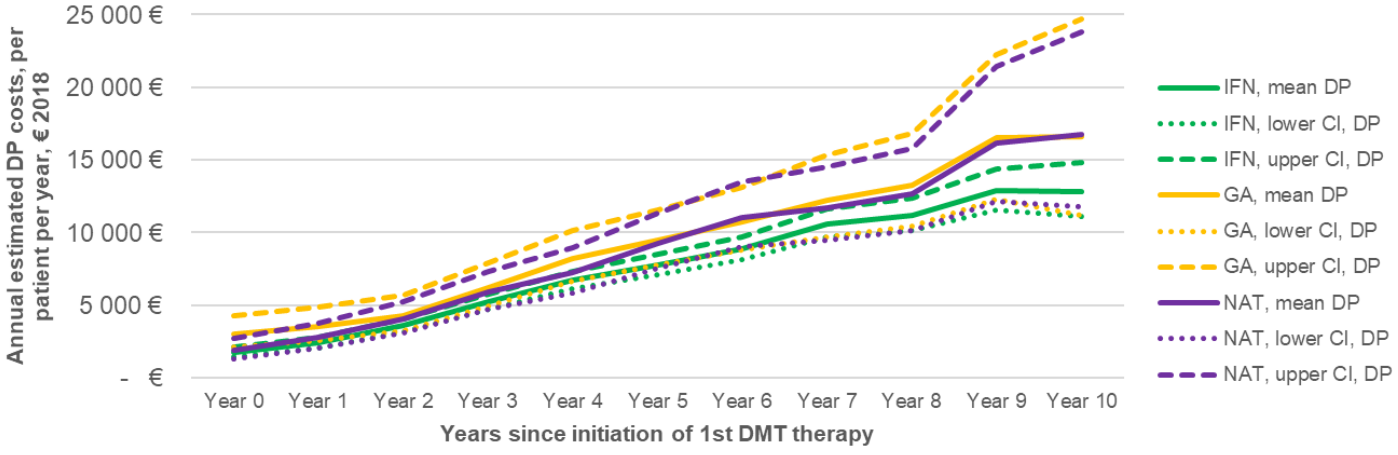

STROBE Statement—Checklist of items that should be included in reports of ***cohort studies***

	Item No	Recommendation	Page No
**Title and abstract**	1	(*a*) Indicate the study's design with a commonly used term in the title or the abstract	1
		(*b*) Provide in the abstract an informative and balanced summary of what was done and what was found	2
**Introduction**			
Background/rationale	2	Explain the scientific background and rationale for the investigation being reported	4
Objectives	3	State specific objectives, including any prespecified hypotheses	4
**Methods**			
Study design	4	Present key elements of study design early in the paper	5
Setting	5	Describe the setting, locations, and relevant dates, including periods of recruitment, exposure, follow-up, and data collection	5-6
Participants	6	(*a*) Give the eligibility criteria, and the sources and methods of selection of participants. Describe methods of follow-up	5-6
		(*b*) For matched studies, give matching criteria and number of exposed and unexposed	n.a.
Variables	7	Clearly define all outcomes, exposures, predictors, potential confounders, and effect modifiers. Give diagnostic criteria, if applicable	5-9
Data sources/ measurement	8*	For each variable of interest, give sources of data and details of methods of assessment (measurement). Describe comparability of assessment methods if there is more than one group	5-7
Bias	9	Describe any efforts to address potential sources of bias	5-7
Study size	10	Explain how the study size was arrived at	5
Quantitative variables	11	Explain how quantitative variables were handled in the analyses. If applicable, describe which groupings were chosen and why	8-9
Statistical methods	12	(*a*) Describe all statistical methods, including those used to control for confounding	8-9
		(*b*) Describe any methods used to examine subgroups and interactions	8-9
		(*c*) Explain how missing data were addressed	7-9
		(*d*) If applicable, explain how loss to follow-up was addressed	6-9
		(*e*) Describe any sensitivity analyses	n.a.
**Results**			
Participants	13*	(a) Report numbers of individuals at each stage of study—eg numbers potentially eligible, examined for eligibility, confirmed eligible, included in the study, completing follow-up, and analysed	10
		(b) Give reasons for non-participation at each stage	5-10
		(c) Consider use of a flow diagram	n.a.
Descriptive data	14*	(a) Give characteristics of study participants (eg demographic, clinical, social) and information on exposures and potential confounders	10
		(b) Indicate number of participants with missing data for each variable of interest	10
		(c) Summarise follow-up time (eg, average and total amount)	10
Outcome data	15*	Report numbers of outcome events or summary measures over time	10

For peer review only

Main results	16	(*a*) Give unadjusted estimates and, if applicable, confounder-adjusted estimates and their precision (eg, 95% confidence interval). Make clear which confounders were adjusted for and why they were included	10, 21-26
		(*b*) Report category boundaries when continuous variables were categorized	n.a.
		(*c*) If relevant, consider translating estimates of relative risk into absolute risk for a meaningful time period	n.a.
Other analyses	17	Report other analyses done—eg analyses of subgroups and interactions, and sensitivity analyses	10, 21-26
**Discussion**			
Key results	18	Summarise key results with reference to study objectives	11-15
Limitations	19	Discuss limitations of the study, taking into account sources of potential bias or imprecision. Discuss both direction and magnitude of any potential bias	13-15
Interpretation	20	Give a cautious overall interpretation of results considering objectives, limitations, multiplicity of analyses, results from similar studies, and other relevant evidence	11-15
Generalisability	21	Discuss the generalisability (external validity) of the study results	14-15
**Other information**			
Funding	22	Give the source of funding and the role of the funders for the present study and, if applicable, for the original study on which the present article is based	16-17

*Give information separately for exposed and unexposed groups.

Note: An Explanation and Elaboration article discusses each checklist item and gives methodological background and published examples of transparent reporting. The STROBE checklist is best used in conjunction with this article (freely available on the Web sites of PLoS Medicine at http://www.plosmedicine.org/, Annals of Internal Medicine at http://www.annals.org/, and Epidemiology at http://www.epidem.com/). Information on the STROBE Initiative is available at http://www.strobe-statement.org.

For peer review only

For peer review only - http://bmjopen.bmj.com/site/about/guidelines.xhtml
